# A four year survey reveals a coherent pattern between occurrence of fruit bodies and soil amoebae populations for nivicolous myxomycetes

**DOI:** 10.1038/s41598-018-30131-3

**Published:** 2018-08-03

**Authors:** Mathilde Borg Dahl, Oleg Shchepin, Christian Schunk, Annette Menzel, Yuri K. Novozhilov, Martin Schnittler

**Affiliations:** 1 Institute of Botany and Landscape Ecology, Ernst Moritz Arndt University Greifswald, Greifswald, Germany; 2 V.L. Komarov Botanical Institute of the Russian Academy of Sciences, St, Petersburg, Russia; 3 Ecoclimatology, Technical University of Munich, Freising, Germany; 4 Institute for Advanced Study, Technical University of Munich, Garching, Germany

## Abstract

Among soil-inhabiting protists, myxomycetes stand out by their macroscopic fructifications which have allowed studies on their ecology and distribution for more than two hundred years. One of the most distinct ecological guilds in myxomycetes are the nivicolous or “snowbank” myxomycete species, which produce fruit bodies at the edge of melting snowbanks in spring. Relationship between the occurrence of fructifications and myxamoebae remain unknown. In this study we used modern molecular techniques, by direct DNA amplification from soil extracts (NGS metabarcoding) to compare the distribution of soil-inhabiting myxamoebae found in 2016 with fructifications from the same sites collected over the course of four years (2013, 2015–17) along an elevational transect in the northern German Alps. A coherent community composition between fructification and soil myxamoebae, though with species-specific differences in relative abundance, was revealed. Although patterns varied among species, myxamoebae were found at both low and high elevations, whereas fruit bodies were mainly found at higher elevations, likely explained by the presence of a stable and long-lasting snow cover. In addition, a year to year comparison of fructification records support the hypothesis that the abundance of fructifications strongly depends on the onset of snowfall in the previous autumn and the soil temperature regime throughout the winter.

## Introduction

Soil protists are a diverse assemblage of basal Eukaryotic lineages^1,2^, which play a crucial role in decomposition where they through their predation regulate and stimulate activity of other microbes and consequently influence higher trophic levels and plant growth^3–8^. Nearly always belonging to the invisible world, these organisms are understudied, due to difficulties in cultivation and the paucity of morphological characters, which complicates diversity studies based on morphological species differentiation. Among soil-inhabiting protists, myxomycetes are an exception due to their macroscopic fructifications^9^ which have been studied for more than two hundred years^10^. Based solely on morphological characters of the fructifications, ca. 1000 species are described^11^ and a substantial body of data on ecology and distribution for these fructifications exists^12^. However, knowledge on abundance of myxamoebae and structure of their communities in natural environments is scarce. Advances in molecular techniques, such as direct DNA amplification from environmental extracts (ePCR) using next-generation sequencing technology (NGS), have made studies of myxamoebae possible. Myxamoebae are currently estimated to account for between 5 to almost 50% of all soil amoebae and be present in a wide variety of soils^13–15^. As for many microbial groups^16^, the true diversity of myxomycetes seems to be much greater than shown by morphological characters, and many described morphospecies seem to consist of several, reproductively isolated, cryptic biological species^17–21^.

Based on field collections of myxomycete fruit bodies, morphospecies are known to require specific habitats such as herbivore dung or decaying wood^22–24^. One of the most distinct ecological guilds in myxomycetes are the nivicolous or “snowbank” species. Their specific habitat along the edge of melting snowbanks was first described in 1908 by Meylan^25^. These myxamoebae thrive and multiply in snow-covered soil^26^, benefiting from the constant temperatures of 0 to 1 °C maintained by the snowpacks^27^. Diversity patterns of fructifications of the ca. 100 described nivicolous morphospecies indicate species-specific preferences for altitude and vegetation^27–32^. However, a quantitative comparison between the occurrence of fruit bodies and myxamoebae along a natural elevation gradient across various vegetation types has never been conducted.

Recent studies have shown that the myxamoebae are quite susceptible to low temperatures^33^. Results of a study from the northern Caucasus using data loggers^27^ suggested that the abundance of fructifications strongly depends on (i) the onset of snowfall in the previous autumn and (ii) the duration of contiguous snow cover throughout the winter.

This paper compares a quantitative survey of fruit bodies (from four years) from a transect of the German northern limestone Alps with a dataset of soil amoebal populations of nivicolous myxomycetes obtained by environmental PCR from the same transect (one year, publication under review^34^). We ask if 1) the occurrence of fructifications reflects that of myxamoebae in soil and 2) if year-to-year variations in fructification records can be explained by winter temperatures and the stability and duration of the snow cover.

## Results

### Alpha diversity and year-to-year comparison

**Figure 1:**
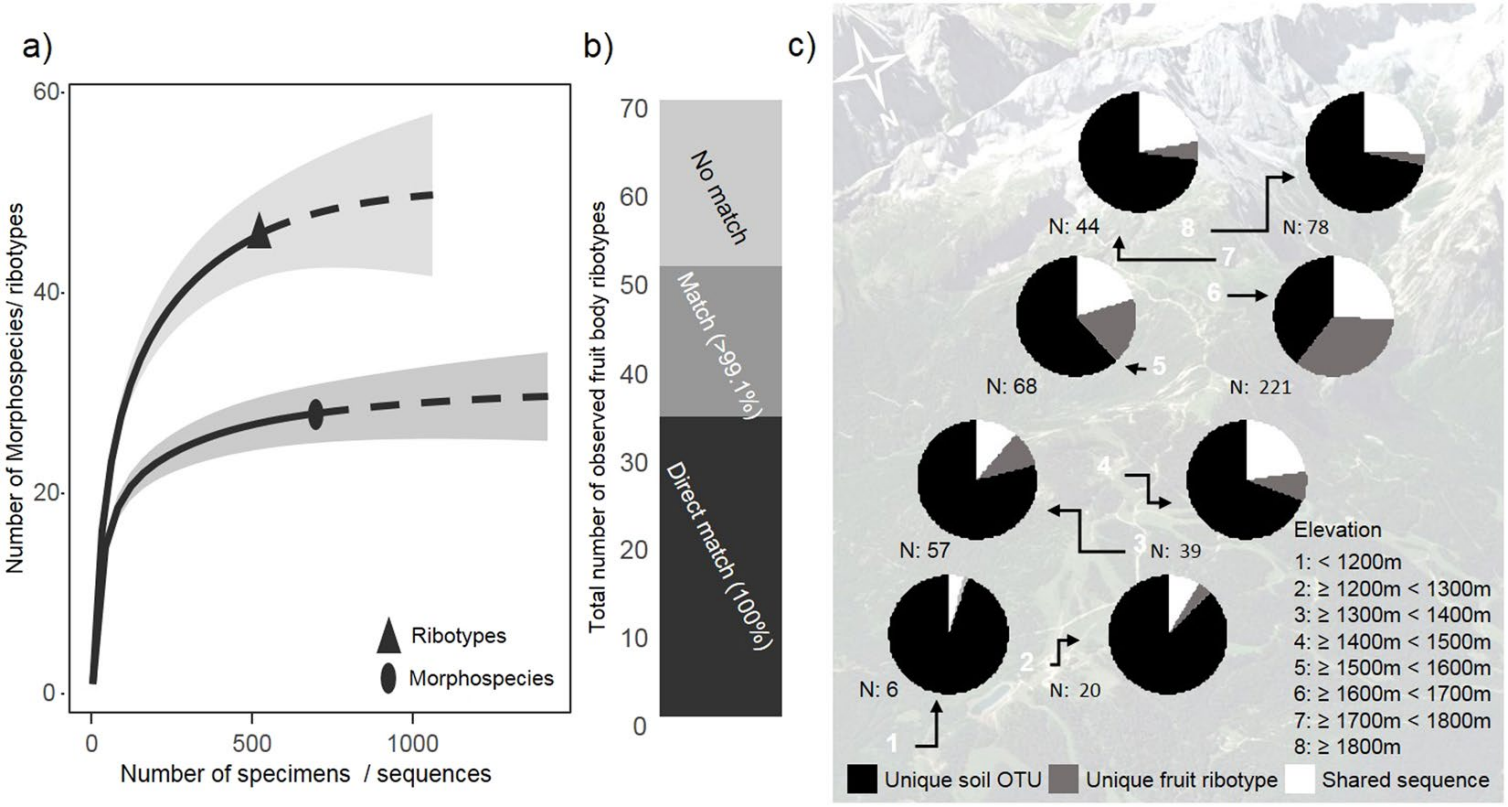
(**a**) Species accumulation curves for morphospecies collected as fruit bodies (circle) and their ribotype clusters (triangle); symbols mark the final point for the accumulation curves. Both curves are extrapolated to double collection size and the 95% confidence intervals are given (grey bands). (**b**) Numbers of unique ribotypes derived from fruit bodies (total 70) showing a direct match (black), >99.1% similarity (dark grey) or less (light grey) to a soil sequence. (**c**) Proportion of observed OTUs (ePCR from soil)/ribotypes (fruit bodies) which are either unique for a soil sample (black), unique for the collections from this elevational belt (grey) or shared between the two datasets (99.1% similarity match; white). Eight plots (white numbers) in 100 m elevational belts were established for soil samples (arrows link their exact position to pie diagrams) and all specimens collected from this belt were assigned (with N indicating the number of successfully sequenced specimens). Map data: Google, DigitalGlobe.

In total 732 colonies of fruit bodies were collected over four years of survey, of these 714 specimens could be successfully determined and assigned to 30 morphospecies, all known as nivicolous (Supplementary S1). Three specimens belonged to the bright-spored genus *Trichia* (*T. alpina* and *T. sordida*) and were thus excluded from the comparison with soil OTUs (Operational Taxonomic Unites), as the primers used to target myxamoebae do not amplify these. A total of 537 specimens (75.3% of all determinable records) were sequenced to allow a direct comparison of ribotypes used as species barcodes^20^ between fruit bodies (collections) and amoebae (recovered by ePCR of soil). All but four sequences clustered within the clade forming the respective morphospecies – at this level virtually no contradictions were found between the morphospecies determination and barcoding results (Supplementary S2). However, one morphospecies often included several ribotypes. The 533 clearly assignable sequences from 28 morphospecies represented 70 unique ribotypes. For 12 morphospecies only a single ribotype was found, but four of these were only represented by a single collection. Clustering of these sequences with the same similarity threshold as used to delimit OTUs from ePCR of soil (99.1%) resulted in 45 ribotype clusters. Taking into account deviations caused by rare mutations and sequencing errors, these 45 clusters were considered to represent truly unique ribotypes (Supplementary S1,S2). This resulted in an estimated diversity coverage of 92.4% for morphospecies and 90.9% for ribotypes (Fig. 1a).

Soil samples were successfully sequenced with high quality (Supplementary S3; Borg Dahl *et al*.^34^), and 208 OTUs of dark-spored myxomycetes were identified (on average 26 ± 9 OTUs per sample ranging from 9 to 43 OTUs). Of these 63 (representing 40.6% of all reads) could be assigned with 98% similarity to reference sequences from fruit bodies, representing a total of 33 morphospecies (Table 1; Supplementary S4 and Borg Dahl *et al*.^34^). Twenty-five of the 33 morphospecies belonged to nivicolous species (Table 1).

**Table 1:** Comparison of ribotypes and morphospecies found from fruit bodies and ePCR of soils.

Observed	Soil samples	Fruit bodies	Match in ePCR with similarity	
	ePCR	Collection	100%	99.1%
Myxomycete sequences	838,553	537		
Unique sequences (ribotypes)	279,747	70	34	53
Assigned to morphospecies	340,829 (40.6%)	533 (99.3%)		
Of these nivicolous species	261,188 (76.6%)	533 (100.0%)		
OTUs/Ribotype clusters	208	45	n.a.	n.a.
Annotated OTUs/Ribotype clusters	63	45	n.a.	n.a.
Morphospecies^a^	33	28 (+2)^b^	24	n.a.
Nivicolous morphospecies	25	28 (+2)^b^	24	n.a.
**Expected (Chao richness estimator** ^**c**^ **)**				
OTUs/Ribotype clusters	208.8	55.1		
Morphospecies	34.0	30.3		

^a^Morphospecies for soil samples were assigned from a data base of sequences from fruit bodies^20^, for collections according to morphological determination.

^b^Two morphospecies belong to the bright-spored clade of myxomycetes and cannot be compared with results from ePCR.

^c^Estimated species richness calculated as asymptotic diversity estimates^64,68^.

Of the 70 unique ribotypes obtained from fruit body-derived sequences 34 were identical to a sequence from soil (100% similarity, Fig. 1b) and additionally 19 had a match to a soil sequence within the threshold for OTU picking (99.1% similarity; assumed to represent the same taxon). Finally, 17 sequences (13 ribotype clusters, 6 from singleton collections) representing 79 specimens were recorded only from fruit bodies and had no >99.1% match to a soil sequence (Supplementary S5). On average, these ribotypes had a match to a soil OTU with 94.1% ± 4.8% similarity (ranging from 87.3% to 98.8% similarity). One of these was a single collection of *Lamproderma pulchellum*. However, also from the complex morphospecies *L. ovoideum* agg. (common in both the ePCR-based and the fruit body-based inventory) three ribotype clusters (42 sequences) from fruit bodies had no match among OTUs from soil with the chosen threshold. Specimens with ribotypes showing a lesser than 99.1% match were found in all the survey years with abundant fruiting (2013: 8 ribotypes, 2015: 7, 2016: 10). There was a strong correlation between the number of specimens sequenced from a site and the number of unique fruit body sequences as well as shared sequences with the soil OTUs (Pearson’s R = 0.94 and 0.98, respectively, Fig. 1c).

**Figure 2:**
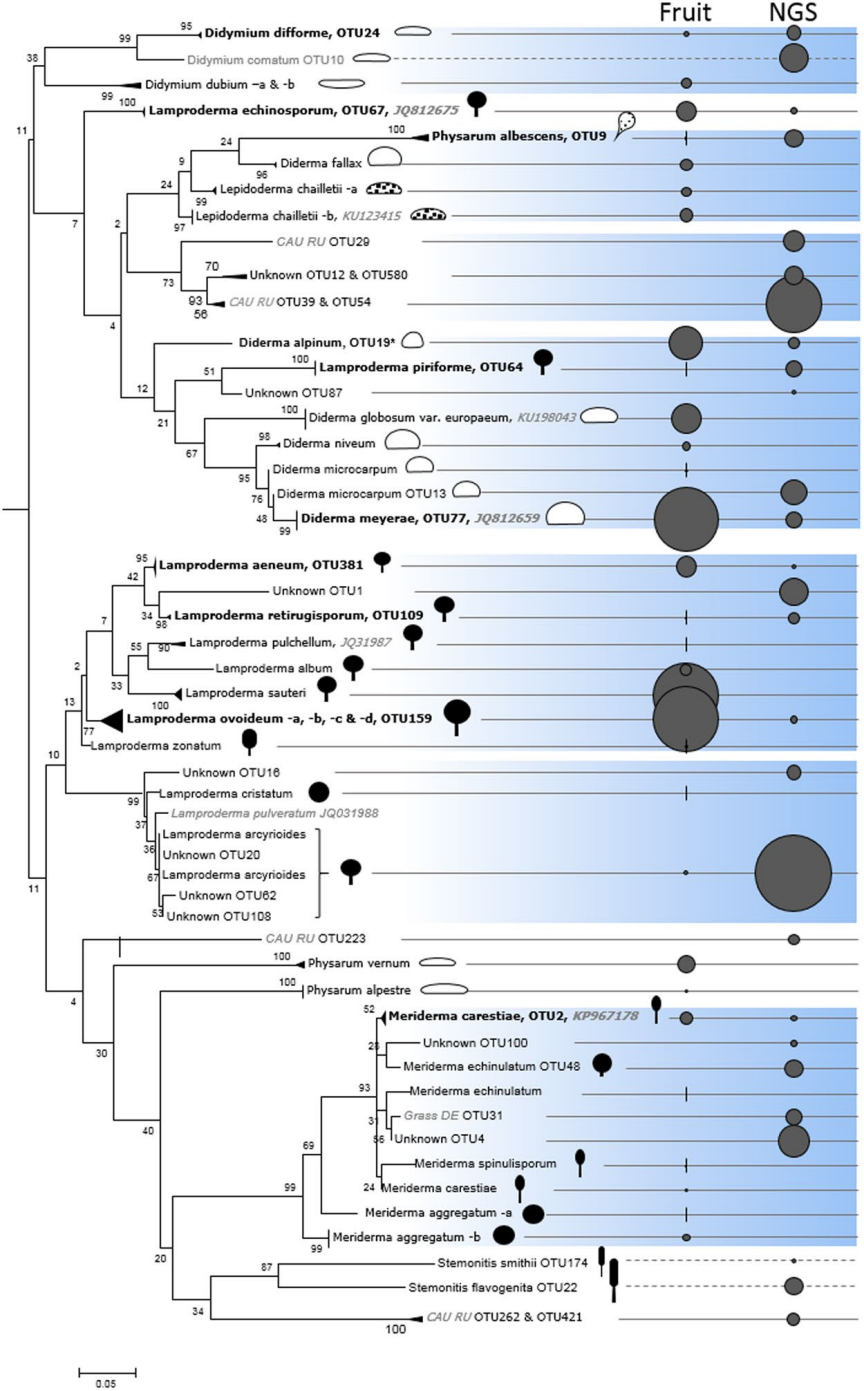
Maximum likelihood phylogenetic tree (500 bootstraps) of partial SSU 18S rDNA sequences originating from soil OTUs and from collected fruit bodies, plus reference sequences not obtained in this study (publicly available from NCBI and indicated by an accession number printed in grey Italics). All fruit body sequences are included together with soil OTUs with a relative abundance of >5% in any two samples (84 OTUs accounting for 64.8% of the reads). Suffixes -a to -d denote ribotype clusters and thus putative biospecies which cannot be told apart reliably by morphological characters. Exact matched between the two inventories are indicated in bold. The abundance of each sequence is scaled for each inventory (‘Fruit’ and ‘NGS’) and indicated by circles of proportional size on the right (for low abundances vertical lines are presented). For fruit bodies the largest relative abundance is met by ribotypes from *Lamproderma ovoideum* -a accounting for 15.6% of the 533 sequenced specimens, and for the soil the unknown OTU20 accounts for the largest relative abundance with 8.8% of the total 838,553 myxomycete DNA reads. Broken lines indicate non-nivicolous taxa detected by NGS which were not surveyed in the field. Schematic drawings indicate fruit body shapes drawn to relative size, color the degree of peridial calcification, from white = heavily calcified to black = not calcified. Soil OTUs with no match in our database were aligned against uncultured sequences available from 1) a study of soil from three lowland grasslands in Germany^36^ (denoted ‘Grass DE’) and 2) a study of soil from the northern Caucasus mountains, Russia^20^ (denoted ‘CAU RU’). Monophyletic branches of the same morphospecies are collapsed for improved graphic presentation. The scale bar represents the number of substitutions for a unit branch length.

Figure 2 presents a maximum likelihood phylogenetic tree including all fruit body sequences and soil OTUs; for the latter only those with a relative abundance exceeding 5% in any two samples were considered (84 OTUs accounting for 64.8% of the reads). Though the overall alpha diversity of the NGS inventory was larger than that of the fruit bodies (208 OTUs vs. 45 ribotypes clusters), slightly more nivicolous morphospecies were registered in the fruit body inventory in comparison to the NGS. One was the morphospecies *Lamproderma cristatum*, which were not represented in the reference database, since sequences from this morphospecies formed mixed clades with those of *L. spinulosporum* and both are currently excluded from the reference database. Second, *Meriderma carestiae* var. *retisporum* did not form a discrete taxon/clade in the sequence database, since the sequences originating from this morphologically determined variety were identical to sequences representing *M. carestiae*, indicating that the description of this morphological variety may not be justified. Finally, four morphospecies which was represented in the reference database were only recorded as fruit bodies: *Lamproderma pulchellum* (1 record, 0.2% of the 533 sequenced records)*, L. zonatum* (3 records, 0.6%), *Meriderma aggregatum* (10 records, 1.9%) and *Didymium dubium* (12 records, 2.3%). Conversely, *Diacheopsis metallica* known to be a nivicolous species, was only recorded as soil OTUs (with 1.6% of a total of 261,188 reads assigned to nivicolous taxa).

**Figure 3:**
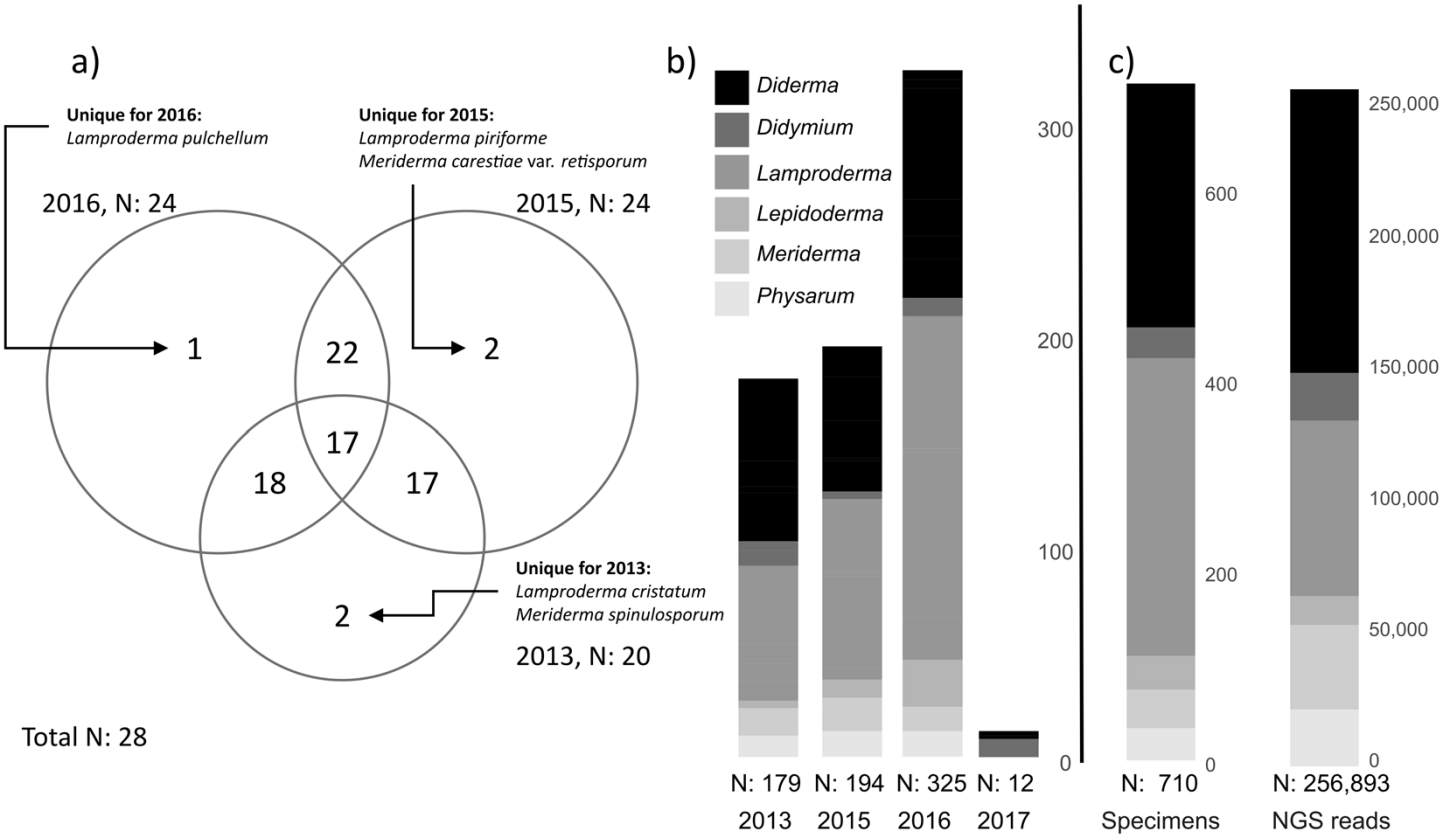
(**a**) Venn diagram showing the number of morphospecies shared between survey years (circle scaled to total number of morphospecies recorded), 2017 is not taken into account due to the low number of records. (**b**) Total number of fructification records (only nivicolous species) within each genus per year. (**c**) Total number of records/reads for the main genera (nivicolous species only) in the complete inventories from collected fruit bodies and ePCR from soil. The genera *Diacheopsis* and *Trichia* are excluded due to their low representation, together accounting for 0.4% (reads, *Diacheopsis* not found fruiting) and 1.8% (collection, *Trichia* as bright-spored species not detected in the ePCR) of the total records.

The following figures only consider nivicolous taxa, not OTUs assigned to other myxomycetes (i.e. with a summer/autumn peak in fructification, which were not recorded during the surveys). A between-year comparison of the collected specimens revealed similar distributions of abundance of genera and morphospecies, and a considerable overlap in registered morphospecies between years (Fig. 3a,b). The poor collection in 2017 was however an exception, with only 12 collected specimens. In contrast, 327 specimens were collected in 2016 (Fig. 3b). All morphospecies recorded more than 20 times were found in each of the years 2013, 2015 and 2016 with good fruiting.

In the fruit body inventory the genera *Lamproderma* and *Diderma* were most abundant accounting for 80% of the collected specimens (Fig. 3c). These genera were also the two most abundant among the annotated NGS reads (accounting for 64% of the reads). The genera *Meriderma, Physarum* and *Didymium* were proportionally more abundant in the NGS record than as collections in the fruit body record (13.2/5.7%, 8.9/4.8% and 7.4/4.5%). The most species-rich genus in both inventories was *Lamproderma* with 7 and 11 morphospecies observed in NGS reads and fruit body records, respectively.

### Elevational species turnover

**Figure 4:**
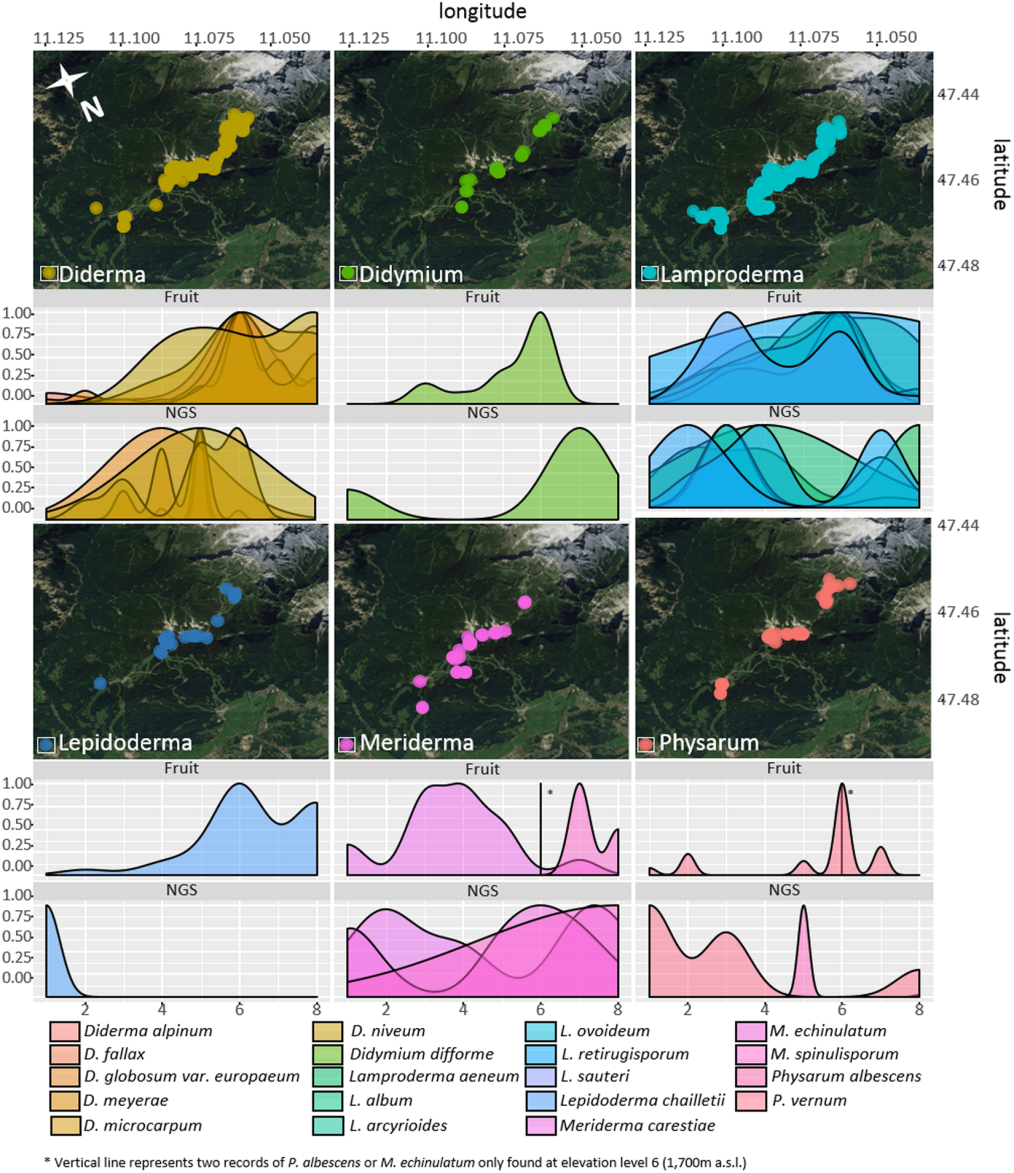
Elevational distribution of records from fructifications/OTUs from ePCR plotted for each genus. The map shows collections of fruit bodies. The two plots below each map show elevational distribution for fruit body records and OTUs plotted along the elevational gradient (Numbers 1 to 8 corresponding to 1,200 to 1,900 m a.s.l. in steps of 100 m). Only nivicolous taxa occurring in both surveys were included. Abundance was scaled to the highest number of records per taxon. OTU records were square root transformed for improved graphic presentation. Map data: Google, DigitalGlobe.

When plotting the records from fruit bodies on a map, it was evident that all major genera of nivicolous myxomycetes were found across the full length of the transect (Fig. 4). However, a peak in records from fruit bodies was observed for most species (except *Meriderma carestiae* and *Lamproderma sauteri*) at the upper-middle part of the transect (level 6; 1,500–1,700 m a.s.l.), whereas no clear peak in abundance across genera was seen for the OTU reads (Fig. 4). For the latter, the average occurrence for all species was significantly (p < 0.001) shifted towards the lower half of the transect (from an average elevation of 1,570 ± 90 m for fruit bodies to 1,440 ± 150 m for soil OTUs). An exception was *Didymium* (though only represented by the morphospecies *D. difforme* with two distinct ribotypes) for which corresponding OTUs were mainly found at the upper half of the transect.

### Snow and temperature regimes

**Figure 5:**
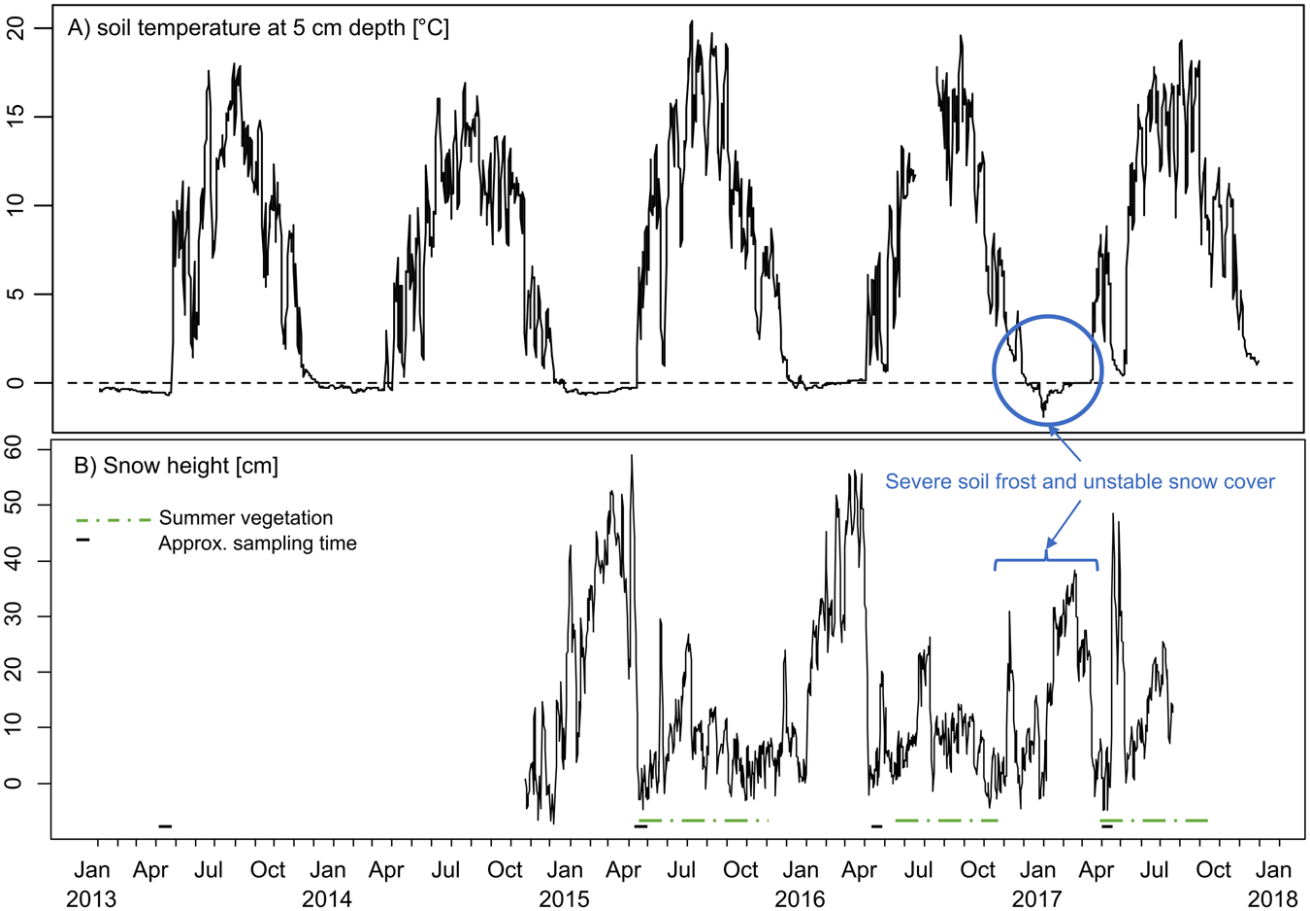
Measurements from January 2013 to October 2017 of (**a**) soil temperature at 5 cm depth and (**b**) snow height (during summer the sensor records the reflection of the surrounding vegetation, delimited by a green dotted line). Short solid lines indicate the time of the four myxomycete surveys. Blue signatures point to a severe soil freezing during winter 2016/17 caused by an instable snow cover. Data were obtained from the TUM climate station ‘Kreuzalm’ (47°27′05′′N, 11°04′20′′E). The snow height sensor was installed in autumn 2014.

Comparisons between the average daily soil temperature at 5 cm depth from 2013 to 2017 and the snow height at the Kreuzalm climate station showed a clear correlation between the presence of a snow cover and the degree of freezing observed in the soil (Fig. 5). This was most clearly visible for the winter 2016/17, when an unstable snow cover did not prevent severe soil freezing (blue circle in Fig. 5).

The estimated number of days where temperatures were considered suitable for amoebal growth (for definition see methods) and the number of days considered unsuitable are summarised for each site as well as for the ‘Kreuzalm’ climate station in Table 2 (see Supplementary S6 for site-specific temperature plots from 2015/16 and 2016/17). In the winter 2015/16 the number of days suitable for amoebal growth was correlated with elevation (Pearson’s R = 0.71, p < 0.005), while no significant correlation was found for unsuitable days (Pearson’s R = 0.49, p = 0.07). On the contrary, in winter 2016/17 the number of days with soil frost (unsuitable for growth) was correlated with elevation (Pearson’s R = 0.68, p < 0.005), while no correlation was seen for the number of suitable growth days (Pearson’s R = 0.32, p = 0.22).

**Table 2:** Calculations for the number of days suitable/non-suitable for amoebal growth for each study site along the elevation gradient in winter 2015/16 and winter 2016/17 based on temperature data from loggers placed in the upper layer of the soil.

Site	Elevation^a^	Winter	Suitable Days^b^	Unsuitable Days^c^
1cl	1195 ± 11	15/16, 16/17	107, 119	0, 2
1op	1202 ± 2	15/16, 16/17	115, 102	5, 33
2cl	1303 ± 14	15/16, 16/17	98, 100	2, 10
2op	1292 ± 3	15/16, 16/17	140, 112	2, 41
3cl	1296 ± 18	15/16, 16/17	129, 131	0, 4
3op	1296 ± 7	15/16, 16/17	123, 86	12, 53
4cl	1400 ± 19	15/16, 16/17	122, 137	0, 22
4op	1368 ± 13	15/16, 16/17	138, 123	4, 32
5cl	1498 ± 9	15/16, 16/17	−^d^, 100	−, 50
5op	1469 ± 22	15/16, 16/17	−, 109	−, 40
Kreuzalm	1578	12/13–16/17	113, 118, 90, 146, 95	55, 18, 59, 0, 23
6cl	1649 ± 25	15/16, 16/17	150, 113	0, 47
6op	1608 ± 24	15/16, 16/17	170, 127	20, 59
7cl	1707 ± 5	15/16, 16/17	150, 115	36, 70
7op	1599 ± 3	15/16, 16/17	209, 168	1, 29
8cl	1903 ± 13	15/16, 16/17	171, 126	6, 56
8op	1883 ± 8	15/16, 16/17	128, 104	21, 62

^a^Mean ± SD for three sites samples as replicates.

^b^Defined as days with daily average temp. between −0.5 and 2 °C and daily fluctuations <3 °C.

^c^Defined as days with maximum temp. <−0.5 °C.

^d^Data loggers for site 5 were lost in winter 2015/16. The same calculations were carried out with data from the ‘Kreuzalm’ climate station site for the years 2013 to 2017, corresponding to the plot in Fig. 5A.

## Discussion

Myxomycetes are assumed to be effective predators of other microbes and thus key players in soil ecosystems and certainly abundant^13^, still very few studies have focused on identifying myxamoebae by ePCR (see Borg Dahl *et al*.^20^). In contrast, a significant body of literature exists on the distribution and ecology of the fructifications^24^. As such, our view on myxomycete ecology resembles that of a gardener who is trying to figure out the structure of an orchard but sees only the apples, not the trees they belong to. Here we report for the first time a study relating results from NGS-assisted metabarcoding of soil amoebal populations to barcoding of the myxomycete fructifications. We find a similar abundance pattern between recorded fruit bodies and soil myxamoebae population of nivicolous myxomycetes, though also a high diversity of unannotated soil OTUs, indicating that fruit body records may not reflect the full soil community. Furthermore we find quantitative fructification records to correlate with patterns of winter soil temperature and snow regime.

### The fruits reflect the trees – Fruit body records vs. NGS records

The recorded diversity of the soil myxamoebae in this study (208 OTUs from 39 samples) are in the range reported by the three available studies targeting soil-inhabiting amoebae of dark-spored myxomycetes. An RNA-based study employed cloning to assess myxamoebal diversity in alpine soil samples from three different mountain sites (France, Scotland and Japan; 74 OTUs from nine samples)^36^. Two DNA-based studies employed Illumina metabarcoding (three German lowland regions, 338 OTUs from 150 samples^37^; alpine soils from the northern Caucasus, 63 OTUs from seven samples^20^).

All 28 dark-spored species collected as fruit bodies were nivicolous and are commonly reported from mountain areas^28,32^ and the most frequently observed species in this study were in accordance with an independent survey from the German Alps reported in 2009^38^. Likewise, the genera *Diderma* and *Lamproderma* were also the most frequently recorded genera in another four-year survey from the northern Caucasus^27^; and in accordance with our findings the species *Diderma meyerae*, *D. globosum* var*. europaeum*, *D. alpinum, Lamproderma ovoideum*, *L. sauteri, Meriderma carestiae* and *Physarum vernum* were found to be the most frequently recorded species from their genera^30^. The same species were recorded with high frequency in a survey from the Khibine Mountains, Kola peninsula^39^. In addition, these authors found *Physarum albescens* to be the most dominant species. This is in accordance with our NGS records (where *P. albescens* turned out to be the most abundant species of *Physarum*), but fruit bodies of this morphospecies were collected only twice.

In our study, most OTUs were recorded only from soil and not represented in our sequences from fruit bodies. Of these, several abundant OTUs (equalling 9.5% of all OTUs) matched a non-nivicolous morphospecies, e.g. *Didymium comatum* and *Stemonitis flavogenita*. Both species were also recorded among soil OTUs from the northern Caucasus at the time of nivicolous fruiting^20^, and fruit bodies are known to occur in autumn (Novozhilov, unpubl. observation). Likewise, nivicolous species were recently detected with NGS metabarcoding in three grassland sites in Germany^36^, although virtually no lowland records of nivicolous species are known from Germany (the lowest altitude for nivicolous records are known from the Black Forest, >800 m a.s.l. and the Harz Mts, >1050 m a.s.l., Schnittler, unpublished; and the Thuringian forest, >800 m a.s.l.^40^). These observations indicate that a collector sees only the “tip of the iceberg”; species may not fruit at a given year although they are present as soil amoebae. We assume that many species are able to survive as amoebae in soils where conditions are not suitable for fruiting.

The community composition of both inventories (collections from fruit bodies and annotated OTU reads, counting nivicolous species only) was found to be quite consistent, although relative abundances between taxa differed considerably. Differences may be caused by variation in numbers of copies of the 18S rDNA gene between taxa^41,42^, which can be related to the size of the amoebae^43^ and/or amplification biases introduced during PCR. For collectors, taxa with large fruit bodies preferring open areas are easier to spot, and were often overrepresented as specimens compared to OTU reads. Among species with white (calcareous) fructifications, prominent examples were *Diderma alpinum, D. meyeri*, *D. globosum* var. *europaeum* or *Physarum vernum*. However, *Diderma microcarpum* and *Physarum albescens* (both comparably easy to detect) seemed underrepresented as specimens (8 and 2 records) in comparison to OTU reads. In addition to the survey from the Khibine Mountains mentioned above, *P. albescens* was extremely abundant in a recent survey of the Rocky Mountains, Denver, Colorado (Borg Dahl, unpublished data). Both regions (Khibine and Rocky Mountains) are cooler and dryer than the northern Alps (cold continental vs. temperate oceanic climate according to the Köppen classification^44^), suggesting that conditions suitable for fruiting were not present at the time where our sites were surveyed.

Thirteen of the 45 ribotype clusters observed from fruit body records were not found among the OTU reads. We expect ribotypes represented as fruit bodies to have reached a high amoebal population size in the soil prior to fruit body formation^45^ and thus it was surprising that not all ribotypes obtained from specimens collected in 2016 were discovered among the OTU reads. A ready explanation is the different area covered by the fruit body-based surveys (>30 ha) in comparison to the plots for collecting soil samples (ca. 48 m^2^), especially when considering the patchy distribution of myxamoebae^34^. Thus fruit bodies of rare species may have been collected in habitats not represented by the soil samples. In addition, the majority (57.0%) of the ribotypes only recorded from fruit bodies originated from other survey years than 2016 when the soil samples were collected, suggesting that the community composition might fluctuate between years and may be influenced by the ability of myxomycetes to undergo long distance dispersal^46,47^.

### Elevational species distribution

For some taxa the elevational distribution patterns observed from specimens and OTU reads were largely in accordance with each other, examples are *D. difforme, M. carestiae* and *M. spinulisporum*. However, for the majority of species their elevational distribution was shifted towards higher elevations in the fruit body inventory compared to the OTU inventory. This holds true for all members of the genus *Diderma* as well as for some species of *Lamproderma (L. album* and *L. arcyrioides*) and *Physarum vernum*. Spring snowmelt happens gradually from the valleys towards the summits and cold spells may often re-cover the summits in snow. Within our surveys we found most often optimum conditions between 1,500 and 1,700 m; at lower elevations the snowmelt was advanced and the snow sometimes nearly completely gone, whereas higher elevations had still a contiguous snow cover. Therefore it cannot be ruled out that the shifted distribution observed between the occurrences of taxa in the NGS metabarcoding data and the fruit body records reflects a bias introduced by the survey timing, although the lower parts of the transect were always carefully examined for remains of fruit bodies, which are quite durable especially in the genera *Lamproderma* (with a flexible columella) and *Diderma* (with a calcareous hypothallus^35^).

### Snow regime dictates fructification success

This study mounts further evidence for the hypothesis that soil freezing has severe consequences for the development of myxomycete fructifications in the following spring, which is in accordance with observations from the northern Caucasus^27^. Furthermore soil freezing was found among the main explanatory parameters for bacterial community composition in a recent study from the Swiss Alps^48^. In comparison to previous years, the winter 2016/17 brought severe soil frost along with a more unstable snow cover (only 90 days of snow cover recorded from Kreuzalm climate station); and the following spring survey resulted in only 12 collected specimens. In the previous winter (2015/16) no soil frost was observed and a long lasting snow cover was registered at ‘Kreuzalm’ climate station (146 days; the longest within our survey period) which was followed by the largest collection made within the investigation period (325 specimens). Going further back to the winter 2014/15, the growing conditions seemed to be similarly ‘bad’ as for the winter 2016/17 with 59 days of permanent soil frost and only 90 days of estimated snow cover. The subsequent survey resulted in 194 collected specimens, which was much less than in spring 2016 but substantially more than in 2017. This indicates that the severity of the soil freezing is of equal importance for the fructification success. The lowest soil temperature registered at the Kreuzalm climate station in winter 2016/17 was −2.8 °C on the 2^nd^ January 2017 in a week where minimum daily soil temperatures were on average −1.8 ± 0.5 °C accompanied by average minimum surface air temperatures −11.6 ± 7.6 °C. In comparison, the lowest registered soil temperature in the winter 2014/15 was −0.7 °C which was registered for a long continuous period, and the daily minimum soil temperatures ranged between −0.3 °C and −0.7 °C throughout the entire winter. In accordance with culture attempts^33^ we assume that frost events severely reduce numbers of myxamoebae, caused by either direct cell die off from a rapid drop in temperature (presumably the case in winter 2016/17) or by lowered growth rates as a result of consistently low temperatures (presumably the case in winter 2014/15). Both cases would result in a lower amoebal density in the soil, which would subsequently lower the success rate for the individual amoebae to encounter a compatible mating type and form a plasmodium^44^. However, to our knowledge quantitative measures of the myxamoebal population dynamics in natural environments have never been attempted. A targeted analysis including direct cell counts from soil extracts and/or quantitative molecular techniques such as qPCR may be a next step to elucidate the mechanisms of how temperature affects the fruiting success in different myxomycete species.

## Conclusion

Our analysis of four years of fructification records revealed an overall consistent pattern in observed taxa between years. In addition, fructifications were largely found to reflect the soil community of myxamoebae. Together with the already long known evidence for species-specific habitat requirements, the finding that fruit body-based surveys reflect relatively well the soil amoebal community represents a unique possibility to include myxomycetes as a microbial representative in classical monitoring programmes and/or citizen science initiatives. Thus, myxomycetes may indicate specific habitat features in the same way as plants, lichens, insects, amphibians etc. To fully profit from myxomycetes as an indicator group, more studies need to target the interplay and associations between myxomycetes and other members of the microbial community. However, the relatively high proportion of OTU reads that clearly belong to myxomycetes but have never been found in any fruit body-based survey^34^ indicates that there is more diversity in the soil than we see as fruit bodies.

From our sequential climate monitoring and fruit body surveys we found strong evidence that soil temperature regime during winter is crucial for the fruiting success of myxomycetes. Similar effects can undoubtedly be expected to occur for other microbial groups. Therefore, changing snow and temperature regimes (as expected from the current climate changes) might have far reaching consequences for the local soil microbial community.

## Materials and Methods

### Study area and specimen collection

The study area was previously described in Borg Dahl *et al*.^34^. In brief: the area is an east exposed transect below the Alpspitz summit near Garmisch-Partenkirchen in the German limestone Alps reaching from the Bayernhaus (47°27′55′′N, 11°12′40′′E, ca. 1,200 m a.s.l.) to the Osterfelder Kopf (47°26′20′′N, 11°03′00′′E, ca. 2,050 m a.s.l.). The lowermost parts of the transect support mixed forests with beech (*Fagus sylvatica* L.), fir (*Abies alba* Mill.) and spruce (*Picea abies* (L.) H. Karst.); with increasing elevation the two former species gradually drop out, and from 1,400 m onwards spruce dominates. The timberline is reached at ca. 1,700 m, and forests give place to shrub thickets composed of *Pinus mugo* Turra and *Alnus alnobetula* (Ehrh) K. Koch (=*A. viridis* (Chaix) DC.). Open grasslands occur at all elevations, either as small natural wet meadows or as semi-natural pastures in the forest belt, where trees have been cut. In places with wet soil and/or strongly affected by cattle grazing, broadleaved forbs (*Rumex alpinus* L., *Senecio alpinus* (L.) Scop.) dominate. One open (op) canopy site and one closed (cl) were chosen at every 100 m elevation (to cover the full range of potential habitats), resulting in 16 sites. At these locations soil samples were collected (June 1^st^, 2016) in three biological replicates approximately 10 meters apart from each other, resulting in 48 samples. Ca. 20 g of soil from the upper most soil layer (excluding litter) was collected within 1 m^2^ and pooled for one sample (see Borg Dahl *et al*.^34^ for further details). Environmental parameters such as soil pH, slope exposition and inclination were recorded for each site.

The entire transect was effectively surveyed for nivicolous myxomycetes during the time of snow melt in spring 2013 (May 9–12), 2015 (Apr 30-May 8), 2016 (Apr 21-May 3 and June 1–4) and 2017 (May 29-June 3) by two investigators. From all observed fructifications of myxomycetes a part of a colony was collected, and boxed immediately in the field to avoid cross-contamination by spores^30^. Myxomycete colonies found further than 20 cm apart from each other were collected separately. For each collected colony the aspect and inclination of the slope as well as GPS coordinates were recorded using GPS status & Toolbox (MobiWIA) application for smartphone (see Supplementary S1 for a collection list).

According to Schnittler *et al*.^27^ the number of days suitable for amoebal growth can be estimated as days with a daily average soil temperature between −0.5 and 2 °C as well as with fluctuations not exceeding three degrees, indicating the presence of an insulating layer of snow, which should provide optimum conditions for amoebal growth of nivicolous myxomycetes and undersnow microbial communities^26^. Likewise days with soil frost, considered unsuitable for growth and potentially lethal for the soil myxamoebae, were defined as days when temperature did not rise above −0.5 °C^33^. In May 2015 Hobo Pro v2 U23 data loggers were installed at the collection sites in a bottom-up perforated plastic cup and placed in level with the soil surface, logging temperature every 30 minutes from October to June each year. In addition, soil temperatures and snow cover data from 2013 to 2017 were obtained from the climate station ‘Kreuzalm’ (47°27′05′′N, 11°04′20′′E) belonging to the section of Ecoclimatology, Technical University of Munich (TUM) situated in close proximity of the 5^th^ survey plot at the middle of the transect (1,578 m.a.s.l.). The station logs soil temperature at 5 cm depth every 10 min, in autumn 2014 a sensor monitoring snow height was installed.

### Morphological determinations

Specimens were determined according to Neubert *et al*.^49,50^ and Poulain *et al*.^47^, applying the morphological species concept of the latter authors for the genera *Diderma* and *Meriderma*. Microscopic characters were assessed by mounting material in Hoyers Medium and using a Leica DM2500 microscope. All material was deposited in the personal collection of M. Schnittler (Greifswald, Germany) to be stored at the Botanical State Collection, Munich (M); duplicates of many specimens were additionally deposited in mycological herbarium of the V.L. Komarov Botanical Institute, St. Petersburg, Russia (LE). Nomenclature follows Lado (2005–2018)^11^, with exception of some tentatively described species (e.g. *Meriderma aggregatum* ad. int.^47^).

### DNA extraction from fruit bodies

DNA from myxomycete fruit bodies was extracted following previously published protocols^20^. In brief, 3–6 fully mature sporocarps (fruit bodies) were mechanically homogenized with a MP Biomedical Fastprep 24 (Santa Ana, California, USA). DNA was extracted with the OMEGA Plant Kit (Norcross, Georgia, USA) according to manufacturer’s protocol. A partial SSU sequence (~350 bp) was amplified using the primers S31R^51^ and S3bF^52^ which are specific for dark-spored myxomycetes (the ca. 100 described nivicolous species nearly all belong to the dark-spored myxomycetes, one of two basal clades in the group^53,54^). Amplicons were Sanger sequenced at an ABI 3010 platform. Sequences are available from the European Nucleotide Archive (ENA), accessions: LT669954–LT670816 and MG819747–MG820028 (see Supplementary S1).

### DNA extraction from soil samples

For this study we used the community composition (table of Operational Taxonomic Units, OTU) of myxamoebae recently obtained from soil samples (reported in Borg Dahl *et al*.^34^ and available from Supplementary S4) and compared with quantitative fruitification records from the same area. The OTU table contained raw DNA read counts (ranging from 5,623 to 55,791 reads per sample) which in numbers were independent from measured environmental parameters (incl. elevation). Likewise the OTU richness was not correlated with the number of DNA reads, but decreased with elevation^34^.

For DNA extraction from soil 0.25 g fresh soil was lysed and DNA extracted using the epMotion robotic platform (Eppendorf) according to the manufacturer’s protocol. PCR amplifications were done in two steps with the primer pairs: S1/SU19R^55^ and S31R/S3b^51,56^. Paired-end sequencing was performed on an Illumina MiSeq platform (Illumina Inc., for quality evaluation). The newly established^20^ 99.1% similarity threshold for OTU-picking was used to create abundance tables (for OTU evaluation against a mock community see Supplementary S3) and the OTUs were annotated using a custom reference database^20^ (counting 172 morphospecies, 1060 sequences, 410 unique sequences). The 99.1% threshold takes into account the existence of sub-clades within described morphospecies which may constitute putative biospecies^17,57^. Since we must assume that most of these cryptic species are not yet formally described and many may lack attributable morphological characters, OTUs were assigned to morphospecies using a slightly lower similarity threshold (≥98.0%) if accompanied by a confidence score >90% as provided by the ‘utax’-command^58^. OTUs with match scores between 95% and 98% were assigned to the myxomycete genus represented by the ‘best match’ sequence.

### Data analysis

All sequences from fruit bodies were aligned with the online tool MAFFT^59^ applying the FFT-NS-I algorithm (iterative refinement method), excluding from further analyses four specimens where morphological determination and ribotype obviously deviated (most likely caused by cross-contamination). Sequences from the remaining 533 specimens were numbered for unique ribotypes, and pairwise genetic distances according to the ‘greedy clustering’^60^ model were calculated (Supplementary S1). Applying the 99.1% threshold found by Borg Dahl *et al*.^20^, ribotypes were clustered, and the clusters were labelled with the name of the respective morphospecies plus a suffix -a to -e for morphospecies including more than one cluster. For all unique ribotypes a phylogenetic tree was built with the Maximum-Likelihood (ML) algorithm implemented in MEGA6^61^ with 500 bootstraps (Supplementary S2) as well as a combined tree of the most dominant sequences of the fruit ribotypes and soil OTUs. For comparison with the distribution of myxamoebae (OTUs), fruit body records were assigned to eight elevational belts of 100 m width, spanning the range between 1,100 and 1,900 m (35 specimens collected at lower or higher elevations were assigned to the first and last belt, respectively).

Basic statistical analyses were performed in R v. 3.3.3^62^ using mainly the packages ‘vegan’^63^ and ‘iNEXT’^64^. This included species/ribotype accumulation curves according to Chao and Jost (2012)^65^. Graphics were compiled using ‘ggplot2’^66^ and ‘ggmap’^67^. Analysis scripts are provided in Supplementary S7.

### Data availability

Fruit body sequences are available from the European Nucleotide Archive (ENA), accessions: LT669954–LT670816 and MG819747–MG820028.

Illumina raw-files from the soil samples are available from NCBI (BioProject ID: PRJNA418896).

## Electronic supplementary material

Supplementary data S1, S2, S3, S5, S6, S7

Supplementary data S4
