# Supplementary material for: A four year survey reveals a coherent pattern between occurrence of fruit bodies and soil amoebae populations for nivicolous myxomycetes
Source: Sci Rep. 2018 Aug 3;8:11662. doi: 10.1038/s41598-018-30131-3 (PMC6076320; doi:10.1038/s41598-018-30131-3)
Supplement: Supplementary file 1 — Supplementary data S1, S2, S3, S5, S6, S7 [file 41598_2018_30131_MOESM1_ESM.pdf]

Supplementary data for: A four year survey reveals a coherent pattern between occurrence of fruit bodies and soil amoebae populations for nivicolous myxomycetes. M. Borg Dahl, O. Shchepin, C. Schunk, A. Menzel, Y. K. Novozhilov and M. Schnittler

- S1 – List of specimens collected and their morphological determination, including elevation and coordinates of the location (format dd.ddddd°). For sequences the collection number of specimen, the unique ribotype (number), ribotype cluster (number), the taxonomical assignment of the sequences (abbreviation of genus and species plus a suffix for the respective ribotype cluster) and the GenBank entry number is given.
- S2 – Maximum Likelihood phylogenetic tree of the 70 unique ribotypes found within 533 sequenced specimens. Labels include a collection number of a reference specimen, the code of the ribotype cluster (a, b, c etc.) and the number of specimens per unique ribotype.
- S3 – Overview of soil sample quality including OTUs accumulation curves, Illumina quality statistics and evaluation of 99.1% similarity threshold against a mock community.
- S5 – Table showing matches between ribotypes from the sequenced specimens and OTUs recovered from ePCR of 39 soil samples.
- S6 – Site-specific temperature plots from winter 2015/16 and 2016/17.
- S7 – Analysis script (R and cmd command prompt) including ribotype calculations and soil match scores as well as diversity estimates.

Supplementary data S1 for: A four year survey reveals a coherent pattern between occurrence of fruit bodies and soil amoebae populations for nivicolous myxomycetes. M. Borg Dahl, O. Shchepin, C. Schunk, A. Menzel, Y. K. Novozhilov and M. Schnittler

S1 Fruit inventory (specimens collored in red are not included in the analysis)

- for nomenclature see Poulain et al. 2011

Determination				Assigment		Meta-data				
Col. Nr.	Sp. Abbreviation	Genus	Species	Ribo number	Ribotype cluster (99.1% similarity) nr.	Collection year	Elevation	Loc	Lat	NCBI
28503	-indet.-			no sequence		2015	1295	11.10269	47.46498	
26320	-indet.-			no sequence		2013	1582	11.07128	47.45203	
26345	-indet.-			no sequence		2013	1061	11.09587	47.47091	
26410	-indet.-			no sequence		2013	1629	11.066	47.44996	
26438	-indet.-			no sequence		2013	1604	11.07	47.45238	
28392	-indet.-			no sequence		2015	1493	11.08295	47.45669	
28424	-indet.-			no sequence		2015	1646	11.07401	47.4526	
28455	-indet.-			no sequence		2015	1484	11.08193	47.45679	
28485	-indet.-			no sequence		2015	1631	11.07514	47.45258	
28496	-indet.-			no sequence		2015	1620	11.07083	47.45213	
28504	-indet.-			no sequence		2015	1298	11.09631	47.465	
28513	-indet.-			no sequence		2015	1298	11.09631	47.465	
28522	-indet.-			no sequence		2015	1288	11.09633	47.46492	
28946	-indet.-			no sequence		2016	1364	11.08417	47.4621	
26327	DDYdif	Didymium	Didymium difforme	65	1	2013	1582	11.07135	47.45203	LT670104
26332	DDYdif	Didymium	Didymium difforme	65	1	2013	1582	11.07141	47.45203	LT670108
28352	DDYdif	Didymium	Didymium difforme	65	1	2015	1430	11.08154	47.45749	LT670221
28962	DDYdif	Didymium	Didymium difforme	65	1	2016	1379	11.08297	47.46147	MG819882
29029	DDYdif	Didymium	Didymium difforme	65	1	2016	1636	11.08014	47.45456	MG820023
29044	DDYdif	Didymium	Didymium difforme	65	1	2016	1550	11.0815	47.45481	MG819883
26339	DDYdif	Didymium	Didymium difforme	no sequence		2013	1605	11.07083	47.45269	
26400	DDYdif	Didymium	Didymium difforme	no sequence		2013	1605	11.07082	47.45269	
26402	DDYdif	Didymium	Didymium difforme	no sequence		2013	1605	11.07083	47.45269	
26403	DDYdif	Didymium	Didymium difforme	no sequence		2013	1605	11.07084	47.45269	
26423	DDYdif	Didymium	Didymium difforme	no sequence		2013	1626	11.0632	47.44844	
28481	DDYdif	Didymium	Didymium difforme	no sequence		2015	1624	11.07121	47.45159	
28482	DDYdif	Didymium	Didymium difforme	no sequence		2015	1624	11.07122	47.45159	
28494	DDYdif	Didymium	Didymium difforme	no sequence		2015	1624	11.07125	47.45159	
28966	DDYdif	Didymium	Didymium difforme	no sequence		2016	1379	11.08297	47.46147	
26325	DDYdub	Didymium	Didymium dubium	no sequence		2013	1582	11.07133	47.45203	
26418	DDYdub	Didymium	Didymium dubium	no sequence		2013	1626	11.06313	47.44844	
29084	DDYdub	Didymium	Didymium dubium	no sequence		2016	1633	11.06225	47.44611	
29088	DDYdub	Didymium	Didymium dubium	no sequence		2016	1683	11.06281	47.44297	
29256	DDYdub	Didymium	Didymium dubium	no sequence		2016	1903	11.05713	47.44229	
29707	DDYdub-a	Didymium	Didymium dubium	25	3	2017	1830	11.05544	47.44089	MG819944
29710	DDYdub-a	Didymium	Didymium dubium	25	3	2017	2001	11.05283	47.439	MG819804
26331	DDYdub-a	Didymium	Didymium dubium	45	3	2013	1582	11.0714	47.45203	LT670107
26426	DDYdub-a	Didymium	Didymium dubium	45	3	2013	1680	11.06246	47.44752	LT670169
29252	DDYdub-a	Didymium	Didymium dubium	45	3	2016	1893	11.05727	47.44208	MG819985
29711	DDYdub-a	Didymium	Didymium dubium	45	3	2017	1500	11.08106	47.45739	MG819845
29701	DDYdub-b	Didymium	Didymium dubium	64	2	2017	1839	11.05528	47.44078	MG819879
29702	DDYdub-b	Didymium	Didymium dubium	64	2	2017	1839	11.05528	47.44078	MG820020
29703	DDYdub-b	Didymium	Didymium dubium	64	2	2017	1830	11.05544	47.44089	MG819880
29704	DDYdub-b	Didymium	Didymium dubium	64	2	2017	1830	11.05544	47.44089	MG820021
29705	DDYdub-b	Didymium	Didymium dubium	64	2	2017	1830	11.05544	47.44089	MG819881
29706	DDYdub-b	Didymium	Didymium dubium	64	2	2017	1830	11.05544	47.44089	MG820022
296521	DID	Diderma	Diderma sp.	no sequence		2013	1688	11.08086	47.4531	
26281	DIDalp	Diderma	Diderma alpinum	49	4	2013	1221	11.09678	47.46714	LT670079
26282	DIDalp	Diderma	Diderma alpinum	49	4	2013	1221	11.0968	47.46714	MG819987
26289	DIDalp	Diderma	Diderma alpinum	49	4	2013	1657	11.07145	47.4523	LT670081
26317	DIDalp	Diderma	Diderma alpinum	49	4	2013	1701	11.07583	47.45269	LT670097
26348	DIDalp	Diderma	Diderma alpinum	49	4	2013	1657	11.07121	47.4523	LT670119
26353	DIDalp	Diderma	Diderma alpinum	49	4	2013	1657	11.07129	47.4523	LT670123
26364	DIDalp	Diderma	Diderma alpinum	49	4	2013	1688	11.08081	47.4531	LT670131
26387	DIDalp	Diderma	Diderma alpinum	49	4	2013	1604	11.06994	47.45238	LT670148
26388	DIDalp	Diderma	Diderma alpinum	49	4	2013	1604	11.06995	47.45238	LT670149
26425	DIDalp	Diderma	Diderma alpinum	49	4	2013	1626	11.06322	47.44844	LT670168
26427	DIDalp	Diderma	Diderma alpinum	49	4	2013	1680	11.06247	47.44752	LT670170
26444	DIDalp	Diderma	Diderma alpinum	49	4	2013	1626	11.06326	47.44844	LT670178
28403	DIDalp	Diderma	Diderma alpinum	49	4	2015	1622	11.08002	47.45491	LT670265
28413	DIDalp	Diderma	Diderma alpinum	49	4	2015	1635	11.08026	47.45459	LT670271
28444	DIDalp	Diderma	Diderma alpinum	49	4	2015	1488	11.08202	47.45636	LT670294
28454	DIDalp	Diderma	Diderma alpinum	49	4	2015	1374	11.08267	47.46185	LT670303
28459	DIDalp	Diderma	Diderma alpinum	49	4	2015	1881	11.05739	47.44311	LT670306
28468	DIDalp	Diderma	Diderma alpinum	49	4	2015	1902	11.05734	47.44222	LT670314
28498	DIDalp	Diderma	Diderma alpinum	49	4	2015	1203	11.10582	47.46267	LT670337
28507	DIDalp	Diderma	Diderma alpinum	49	4	2015	1298	11.09631	47.465	LT670343
28531	DIDalp	Diderma	Diderma alpinum	49	4	2015	1662	11.08065	47.45312	LT670357
28543	DIDalp	Diderma	Diderma alpinum	49	4	2015	1588	11.07969	47.45473	LT670367
28545	DIDalp	Diderma	Diderma alpinum	49	4	2015	1588	11.07969	47.45473	LT670369
29002	DIDalp	Diderma	Diderma alpinum	49	4	2016	1568	11.08308	47.45553	MG819847
29011	DIDalp	Diderma	Diderma alpinum	49	4	2016	1692	11.07972	47.45233	MG819988
29018	DIDalp	Diderma	Diderma alpinum	49	4	2016	1553	11.07944	47.45494	MG819848
29027	DIDalp	Diderma	Diderma alpinum	49	4	2016	1636	11.08006	47.45503	MG819989
29068	DIDalp	Diderma	Diderma alpinum	49	4	2016	1612	11.06594	47.45039	MG819849
29070	DIDalp	Diderma	Diderma alpinum	49	4	2016	1612	11.06594	47.45039	MG819990
29085	DIDalp	Diderma	Diderma alpinum	49	4	2016	1653	11.06197	47.44581	MG819850
29099	DIDalp	Diderma	Diderma alpinum	49	4	2016	1609	11.06278	47.44608	MG819991
29101	DIDalp	Diderma	Diderma alpinum	49	4	2016	1609	11.06278	47.44608	MG819851
29110	DIDalp	Diderma	Diderma alpinum	49	4	2016	1995	11.06258	47.44247	MG819992
29114	DIDalp	Diderma	Diderma alpinum	49	4	2016	1995	11.06258	47.44247	MG819852
29119	DIDalp	Diderma	Diderma alpinum	49	4	2016	1699	11.06206	47.44211	MG819993
29127	DIDalp	Diderma	Diderma alpinum	49	4	2016	1749	11.06125	47.44236	MG819853
29136	DIDalp	Diderma	Diderma alpinum	49	4	2016	1726	11.06072	27.44253	MG819994
29141	DIDalp	Diderma	Diderma alpinum	49	4	2016	1682	11.07708	47.45219	MG819854
29159	DIDalp	Diderma	Diderma alpinum	49	4	2016	1668	11.06156	47.44436	MG819995
29171	DIDalp	Diderma	Diderma alpinum	49	4	2016	1698	11.06136	47.44397	MG819855
29186	DIDalp	Diderma	Diderma alpinum	49	4	2016	1795	11.05756	47.44036	MG819996
29234	DIDalp	Diderma	Diderma alpinum	49	4	2016	1885	11.05718	47.44228	MG819856
26285	DIDalp	Diderma	Diderma alpinum	no sequence		2013	1221	11.09684	47.46714	
26291	DIDalp	Diderma	Diderma alpinum	no sequence		2013	1657	11.07147	47.4523	
26311	DIDalp	Diderma	Diderma alpinum	no sequence		2013	1699	11.08036	47.45268	
26349	DIDalp	Diderma	Diderma alpinum	no sequence		2013	1657	11.07122	47.4523	
26355	DIDalp	Diderma	Diderma alpinum	no sequence		2013	1657	11.07131	47.4523	
26405	DIDalp	Diderma	Diderma alpinum	no sequence		2013	1604	11.06995	47.45238	
26419	DIDalp	Diderma	Diderma alpinum	no sequence		2013	1626	11.06316	47.44844	
26440	DIDalp	Diderma	Diderma alpinum	no sequence		2013	1604	11.06997	47.45238	
26441	DIDalp	Diderma	Diderma alpinum	no sequence		2013	1604	11.06998	47.45238	
28429	DIDalp	Diderma	Diderma alpinum	no sequence		2015	1625	11.08052	47.45229	
28443	DIDalp	Diderma	Diderma alpinum	no sequence		2015	1501	11.08237	47.4564	
28486	DIDalp	Diderma	Diderma alpinum	no sequence		2015	1870	11.05719	47.4431	
296494	DIDalp	Diderma	Diderma alpinum	no sequence		2013	1657	11.07123	47.4523	
296554	DIDalp	Diderma	Diderma alpinum	no sequence		2013	1657	11.07144	47.4523	
29244	DIDfal	Diderma	Diderma fallax	no sequence		2016	1893	11.0712	47.4523	
26389	DIDfal-a	Diderma	Diderma fallax	24	6	2013	1604	11.06996	47.45238	LT670150
26390	DIDfal-a	Diderma	Diderma fallax	24	6	2013	1604	11.06998	47.45238	MG819800
28458	DIDfal-a	Diderma	Diderma fallax	24	6	2015	1870	11.05725	47.44268	LT670305
28478	DIDfal-a	Diderma	Diderma fallax	24	6	2015	1581	11.07075	47.45191	LT670324
29134	DIDfal-a	Diderma	Diderma fallax	24	6	2016	1726	11.06072	47.44225	MG819941
29178	DIDfal-a	Diderma	Diderma fallax	24	6	2016	1789	11.06	47.43914	MG819801

29239	DIDfal-a	Diderma	Diderma fallax	24	6	2016	1893	11.07119	47.4523	MG819942
29242	DIDfal-a	Diderma	Diderma fallax	24	6	2016	1893	11.0712	47.4523	MG819802
29243	DIDfal-a	Diderma	Diderma fallax	24	6	2016	1893	11.0712	47.4523	MG819943
29262	DIDfal-a	Diderma	Diderma fallax	24	6	2016	1903	11.05713	47.44229	MG819803
296545	DIDfal-a	Diderma	Diderma fallax	24	6	2013	1604	11.06997	47.45238	KU198041
29108	DIDfal-b	Diderma	Diderma fallax	43	5	2016	1680	11.06286	47.44319	MG819983
29162	DIDfal-b	Diderma	Diderma fallax	43	5	2016	1668	11.06156	47.44436	MG819843
29165	DIDfal-b	Diderma	Diderma fallax	43	5	2016	1691	11.06175	47.44433	MG819984
29174	DIDfal-b	Diderma	Diderma fallax	43	5	2016	1698	11.06136	47.44397	MG819844
26283	DIDgloeur	Diderma	Diderma globosum var. europaeum	no sequence		2013	1221	11.09682	47.46714	
26286	DIDgloeur	Diderma	Diderma globosum var. europaeum	no sequence		2013	1277	11.09676	47.46714	
26413	DIDgloeur	Diderma	Diderma globosum var. europaeum	no sequence		2013	1626	11.0631	47.44844	
26420	DIDgloeur	Diderma	Diderma globosum var. europaeum	no sequence		2013	1626	11.06317	47.44844	
26446	DIDgloeur	Diderma	Diderma globosum var. europaeum	no sequence		2013	1626	11.06328	47.44844	
28357	DIDgloeur	Diderma	Diderma globosum var. europaeum	no sequence		2015	1662	11.06341	47.44819	
28378	DIDgloeur	Diderma	Diderma globosum var. europaeum	no sequence		2015	1411	11.0856	47.46005	
28391	DIDgloeur	Diderma	Diderma globosum var. europaeum	no sequence		2015	1358	11.08595	47.46189	
28526	DIDgloeur	Diderma	Diderma globosum var. europaeum	no sequence		2015	1708	11.07828	47.45241	
296510	DIDgloeur	Diderma	Diderma globosum var. europaeum	no sequence		2013	1657	11.07139	47.4523	
26406	DIDgloeur-a	Diderma	Diderma globosum var. europaeum	8	7	2013	1629	11.06596	47.44996	LT670158
28493	DIDgloeur-a	Diderma	Diderma globosum var. europaeum	8	7	2015	1870	11.05725	47.44285	LT670334
26414	DIDgloeur-a	Diderma	Diderma globosum var. europaeum	26	7	2013	1626	11.06311	47.44844	LT670163
26417	DIDgloeur-a	Diderma	Diderma globosum var. europaeum	26	7	2013	1626	11.06315	47.44844	LT670166
26442	DIDgloeur-a	Diderma	Diderma globosum var. europaeum	26	7	2013	1626	11.06324	47.44844	LT670176
28434	DIDgloeur-a	Diderma	Diderma globosum var. europaeum	26	7	2015	1728	11.07888	47.45244	LT670288
28461	DIDgloeur-a	Diderma	Diderma globosum var. europaeum	26	7	2015	1895	11.05754	47.44228	LT670308
28467	DIDgloeur-a	Diderma	Diderma globosum var. europaeum	26	7	2015	1901	11.05733	47.44222	LT670313
28472	DIDgloeur-a	Diderma	Diderma globosum var. europaeum	26	7	2015	1703	11.06102	47.44711	LT670318
28473	DIDgloeur-a	Diderma	Diderma globosum var. europaeum	26	7	2015	1706	11.06147	47.44724	LT670319
28488	DIDgloeur-a	Diderma	Diderma globosum var. europaeum	26	7	2015	1870	11.05719	47.44298	LT670330
28519	DIDgloeur-a	Diderma	Diderma globosum var. europaeum	26	7	2015	1298	11.09631	47.465	LT670352
28540	DIDgloeur-a	Diderma	Diderma globosum var. europaeum	26	7	2015	1588	11.07969	47.45473	LT670364
28541	DIDgloeur-a	Diderma	Diderma globosum var. europaeum	26	7	2015	1588	11.07969	47.45473	LT670365
29028	DIDgloeur-a	Diderma	Diderma globosum var. europaeum	26	7	2016	1636	11.08014	47.45456	MG819945
29049	DIDgloeur-a	Diderma	Diderma globosum var. europaeum	26	7	2016	1523	11.07417	47.45442	MG819805
29082	DIDgloeur-a	Diderma	Diderma globosum var. europaeum	26	7	2016	1633	11.06225	47.44611	MG819946
29094	DIDgloeur-a	Diderma	Diderma globosum var. europaeum	26	7	2016	1662	11.06169	47.44467	MG819806
29098	DIDgloeur-a	Diderma	Diderma globosum var. europaeum	26	7	2016	1609	11.06278	47.44608	MG819947
29126	DIDgloeur-a	Diderma	Diderma globosum var. europaeum	26	7	2016	1702	11.06169	47.44211	MG819807
29132	DIDgloeur-a	Diderma	Diderma globosum var. europaeum	26	7	2016	1749	11.06125	47.44236	MG819948
29133	DIDgloeur-a	Diderma	Diderma globosum var. europaeum	26	7	2016	1726	11.06072	47.44225	MG819808
29137	DIDgloeur-a	Diderma	Diderma globosum var. europaeum	26	7	2016	1716	11.06131	27.44689	MG819949
29170	DIDgloeur-a	Diderma	Diderma globosum var. europaeum	26	7	2016	1698	11.06136	47.44397	MG819809
29187	DIDgloeur-a	Diderma	Diderma globosum var. europaeum	26	7	2016	1795	11.05756	47.44036	MG819950
29700	DIDgloeur-a	Diderma	Diderma globosum var. europaeum	26	7	2017	1846	11.05675	47.44117	MG819810
26358	DIDgloeur-a	Diderma	Diderma globosum var. europaeum	27	7	2013	1657	11.07136	47.4523	LT670126
29087	DIDgloeur-a	Diderma	Diderma globosum var. europaeum	27	7	2016	1650	11.062	47.44539	MG819951
26445	DIDgloeur-a	Diderma	Diderma globosum var. europaeum	66	7	2013	1626	11.06327	47.44844	MG820024
28410	DIDgloeur-a	Diderma	Diderma globosum var. europaeum	66	7	2015	1600	11.07986	47.45462	LT670270
28471	DIDgloeur-a	Diderma	Diderma globosum var. europaeum	66	7	2015	1905	11.05737	47.44222	LT670317
28509	DIDgloeur-a	Diderma	Diderma globosum var. europaeum	66	7	2015	1298	11.09631	47.465	LT670345
29030	DIDgloeur-a	Diderma	Diderma globosum var. europaeum	66	7	2016	1636	11.08014	47.45456	MG819884
29100	DIDgloeur-a	Diderma	Diderma globosum var. europaeum	66	7	2016	1609	11.06278	47.44608	MG820025
29184	DIDgloeur-a	Diderma	Diderma globosum var. europaeum	66	7	2016	1797	11.05783	47.44042	MG819885
29107	DIDgloeur-b	Diderma	Diderma globosum var. europaeum	67	8	2016	1674	11.06211	47.44492	MG820026
29161	DIDgloeur-b	Diderma	Diderma globosum var. europaeum	67	8	2016	1668	11.06156	47.44436	MG819886
26292	DIDmey	Diderma	Diderma meyerae	29	9	2013	1657	11.07148	47.4523	LT670082
26293	DIDmey	Diderma	Diderma meyerae	29	9	2013	1657	11.07149	47.4523	LT670083
26294	DIDmey	Diderma	Diderma meyerae	29	9	2013	1657	11.0715	47.4523	LT670084
26298	DIDmey	Diderma	Diderma meyerae	29	9	2013	1657	11.07154	47.4523	LT670086
26301	DIDmey	Diderma	Diderma meyerae	29	9	2013	1688	11.08096	47.4531	LT670088
26305	DIDmey	Diderma	Diderma meyerae	29	9	2013	1688	11.081	47.4531	LT670091
26308	DIDmey	Diderma	Diderma meyerae	29	9	2013	1699	11.08032	47.45268	LT670092
26312	DIDmey	Diderma	Diderma meyerae	29	9	2013	1684	11.07862	47.45309	LT670094
26316	DIDmey	Diderma	Diderma meyerae	29	9	2013	1684	11.07867	47.45309	LT670096
26318	DIDmey	Diderma	Diderma meyerae	29	9	2013	1701	11.07584	47.45269	LT670098
26323	DIDmey	Diderma	Diderma meyerae	29	9	2013	1582	11.07131	47.45203	LT670101
26347	DIDmey	Diderma	Diderma meyerae	29	9	2013	1657	11.0712	47.4523	LT670118
26351	DIDmey	Diderma	Diderma meyerae	29	9	2013	1657	11.07125	47.4523	LT670121
26352	DIDmey	Diderma	Diderma meyerae	29	9	2013	1657	11.07128	47.4523	LT670122
26354	DIDmey	Diderma	Diderma meyerae	29	9	2013	1657	11.0713	47.4523	LT670124
26359	DIDmey	Diderma	Diderma meyerae	29	9	2013	1657	11.07137	47.4523	LT670127
26361	DIDmey	Diderma	Diderma meyerae	29	9	2013	1657	11.0714	47.4523	LT670128
26362	DIDmey	Diderma	Diderma meyerae	29	9	2013	1657	11.07141	47.4523	LT670129
26363	DIDmey	Diderma	Diderma meyerae	29	9	2013	1688	11.0808	47.4531	LT670130
26367	DIDmey	Diderma	Diderma meyerae	29	9	2013	1688	11.08084	47.4531	LT670134
26370	DIDmey	Diderma	Diderma meyerae	29	9	2013	1688	11.08089	47.4531	LT670135
26373	DIDmey	Diderma	Diderma meyerae	29	9	2013	1688	11.08092	47.4531	LT670137
26377	DIDmey	Diderma	Diderma meyerae	29	9	2013	1701	11.0758	47.45269	LT670140
26378	DIDmey	Diderma	Diderma meyerae	29	9	2013	1701	11.07581	47.45269	LT670141
26379	DIDmey	Diderma	Diderma meyerae	29	9	2013	1701	11.07582	47.45269	LT670142
26424	DIDmey	Diderma	Diderma meyerae	29	9	2013	1626	11.06321	47.44844	LT670167
28396	DIDmey	Diderma	Diderma meyerae	29	9	2015	1588	11.07969	47.45473	LT670258
28400	DIDmey	Diderma	Diderma meyerae	29	9	2015	1622	11.07999	47.45491	LT670262
28423	DIDmey	Diderma	Diderma meyerae	29	9	2015	1646	11.07401	47.4526	LT670281
28431	DIDmey	Diderma	Diderma meyerae	29	9	2015	1629	11.08054	47.4528	LT670285
28433	DIDmey	Diderma	Diderma meyerae	29	9	2015	1728	11.07888	47.45244	LT670287
28462	DIDmey	Diderma	Diderma meyerae	29	9	2015	1896	11.05755	47.44228	LT670309
28466	DIDmey	Diderma	Diderma meyerae	29	9	2015	1900	11.05732	47.44222	LT670312
28483	DIDmey	Diderma	Diderma meyerae	29	9	2015	1624	11.07123	47.45159	LT670327
28533	DIDmey	Diderma	Diderma meyerae	29	9	2015	1665	11.08067	47.45314	LT670359
28534	DIDmey	Diderma	Diderma meyerae	29	9	2015	1665	11.08079	47.45317	LT670360
28538	DIDmey	Diderma	Diderma meyerae	29	9	2015	1684	11.08104	47.45321	LT670362
28539	DIDmey	Diderma	Diderma meyerae	29	9	2015	1665	11.08094	47.45309	LT670363
28551	DIDmey	Diderma	Diderma meyerae	29	9	2015	1616	11.08017	47.45477	LT670371
29010	DIDmey	Diderma	Diderma meyerae	29	9	2016	1692	11.07994	47.45244	MG819952
29012	DIDmey	Diderma	Diderma meyerae	29	9	2016	1694	11.07972	47.45233	MG819812
29014	DIDmey	Diderma	Diderma meyerae	29	9	2016	1694	11.07972	47.45233	MG819953
29019	DIDmey	Diderma	Diderma meyerae	29	9	2016	1553	11.07944	47.45494	MG819813
29021	DIDmey	Diderma	Diderma meyerae	29	9	2016	1565	11.07964	47.45478	MG819954
29022	DIDmey	Diderma	Diderma meyerae	29	9	2016	1636	11.08006	47.45503	MG819814
29036	DIDmey	Diderma	Diderma meyerae	29	9	2016	1605	11.08017	47.45406	MG819955
29054	DIDmey	Diderma	Diderma meyerae	29	9	2016	1625	11.06686	47.45289	MG819815
29058	DIDmey	Diderma	Diderma meyerae	29	9	2016	1625	11.06686	47.45289	MG819956
29061	DIDmey	Diderma	Diderma meyerae	29	9	2016	1613	11.06667	47.45289	MG819816
29066	DIDmey	Diderma	Diderma meyerae	29	9	2016	1650	11.06617	47.451	MG819957
29117	DIDmey	Diderma	Diderma meyerae	29	9	2016	1699	11.06206	47.44211	MG819817
29120	DIDmey	Diderma	Diderma meyerae	29	9	2016	1697	11.06181	47.44228	MG819958
29122	DIDmey	Diderma	Diderma meyerae	29	9	2016	1702	11.06169	47.44211	MG819818
29139	DIDmey	Diderma	Diderma meyerae	29	9	2016	1640	11.07425	47.45256	MG819959
29140	DIDmey	Diderma	Diderma meyerae	29	9	2016	1682	11.07708	47.45219	MG819819
29142	DIDmey	Diderma	Diderma meyerae	29	9	2016	1675	11.08039	47.45317	MG819960
29146	DIDmey	Diderma	Diderma meyerae	29	9	2016	1528	11.07425	47.45433	MG819820
29156	DIDmey	Diderma	Diderma meyerae	29	9	2016	1580	11.07092	47.45178	MG819961
29212	DIDmey	Diderma	Diderma meyerae	29	9	2016	1885	11.0712	47.4523	MG819821
29213	DIDmey	Diderma	Diderma meyerae	29	9	2016	1885	11.0712	47.4523	MG819962
29217	DIDmey	Diderma	Diderma meyerae	29	9	2016	1885	11.0712	47.4523	MG819822

29218	DIDmey	Diderma	Diderma meyerae		29	9	2016	1885	11.0712	47.4523	MG819963
29219	DIDmey	Diderma	Diderma meyerae		29	9	2016	1885	11.0712	47.4523	MG819823
29221	DIDmey	Diderma	Diderma meyerae		29	9	2016	1885	11.0712	47.4523	MG819964
29222	DIDmey	Diderma	Diderma meyerae		29	9	2016	1885	11.0712	47.4523	MG819824
29223	DIDmey	Diderma	Diderma meyerae		29	9	2016	1885	11.0712	47.4523	MG819965
29226	DIDmey	Diderma	Diderma meyerae		29	9	2016	1885	11.0712	47.4523	MG819825
29227	DIDmey	Diderma	Diderma meyerae		29	9	2016	1885	11.0712	47.4523	MG819966
29229	DIDmey	Diderma	Diderma meyerae		29	9	2016	1885	11.0712	47.4523	MG819826
29230	DIDmey	Diderma	Diderma meyerae		29	9	2016	1885	11.0712	47.4523	MG819967
29249	DIDmey	Diderma	Diderma meyerae		29	9	2016	1893	11.05741	47.44159	MG819827
29251	DIDmey	Diderma	Diderma meyerae		29	9	2016	1893	11.05741	47.44159	MG819968
29253	DIDmey	Diderma	Diderma meyerae		29	9	2016	1903	11.05713	47.44229	MG819828
29258	DIDmey	Diderma	Diderma meyerae		29	9	2016	1903	11.05713	47.44229	MG819969
29259	DIDmey	Diderma	Diderma meyerae		29	9	2016	1903	11.05713	47.44229	MG819829
29260	DIDmey	Diderma	Diderma meyerae		29	9	2016	1903	11.05713	47.44229	MG819970
29263	DIDmey	Diderma	Diderma meyerae		29	9	2016	1903	11.05721	47.44254	MG819830
29708	DIDmey	Diderma	Diderma meyerae		29	9	2017	1866	11.05572	47.44036	MG819971
29709	DIDmey	Diderma	Diderma meyerae		29	9	2017	1902	11.05547	47.43981	MG819831
29046	DIDmey	Diderma	Diderma meyerae		47	9	2016	1504	11.07419	47.45436	MG819986
29257	DIDmey	Diderma	Diderma meyerae		48	9	2016	1903	11.05713	47.44229	MG819846
26290	DIDmey	Diderma	Diderma meyerae	no sequence			2013	1657	11.07146	47.4523	
26295	DIDmey	Diderma	Diderma meyerae	no sequence			2013	1657	11.07151	47.4523	
26356	DIDmey	Diderma	Diderma meyerae	no sequence			2013	1657	11.07132	47.4523	
26360	DIDmey	Diderma	Diderma meyerae	no sequence			2013	1657	11.07138	47.4523	
26368	DIDmey	Diderma	Diderma meyerae	no sequence			2013	1688	11.08085	47.4531	
26369	DIDmey	Diderma	Diderma meyerae	no sequence			2013	1688	11.08088	47.4531	
26372	DIDmey	Diderma	Diderma meyerae	no sequence			2013	1688	11.08091	47.4531	
26437	DIDmey	Diderma	Diderma meyerae	no sequence			2013	1893	11.05756	47.4424	
28364	DIDmey	Diderma	Diderma meyerae	no sequence			2015	1609	11.06556	47.44993	
28407	DIDmey	Diderma	Diderma meyerae	no sequence			2015	1622	11.08006	47.45491	
28465	DIDmey	Diderma	Diderma meyerae	no sequence			2015	1899	11.05731	47.44222	
28517	DIDmey	Diderma	Diderma meyerae	no sequence			2015	1298	11.09631	47.465	
28523	DIDmey	Diderma	Diderma meyerae	no sequence			2015	1288	11.09633	47.46492	
28527	DIDmey	Diderma	Diderma meyerae	no sequence			2015	1708	11.07828	47.45241	
28549	DIDmey	Diderma	Diderma meyerae	no sequence			2015	1588	11.07969	47.45473	
29026	DIDmey	Diderma	Diderma meyerae	no sequence			2016	1636	11.08006	47.45503	
29031	DIDmey	Diderma	Diderma meyerae	no sequence			2016	1626	11.08031	47.45433	
29063	DIDmey	Diderma	Diderma meyerae	no sequence			2016	1652	11.06639	47.45119	
29138	DIDmey	Diderma	Diderma meyerae	no sequence			2016	1716	11.06131	27.44689	
29179	DIDmey	Diderma	Diderma meyerae	no sequence			2016	1745	11.06006	47.4405	
29214	DIDmey	Diderma	Diderma meyerae	no sequence			2016	1885	11.0712	47.4523	
29215	DIDmey	Diderma	Diderma meyerae	no sequence			2016	1885	11.0712	47.4523	
29216	DIDmey	Diderma	Diderma meyerae	no sequence			2016	1885	11.0712	47.4523	
29220	DIDmey	Diderma	Diderma meyerae	no sequence			2016	1885	11.0712	47.4523	
29235	DIDmey	Diderma	Diderma meyerae	no sequence			2016	1885	11.05718	47.44228	
29237	DIDmey	Diderma	Diderma meyerae	no sequence			2016	1885	11.05743	47.443	
29254	DIDmey	Diderma	Diderma meyerae	no sequence			2016	1903	11.05713	47.44229	
29255	DIDmey	Diderma	Diderma meyerae	no sequence			2016	1903	11.05713	47.44229	
296497	DIDmey	Diderma	Diderma meyerae	no sequence			2013	1657	11.07126	47.4523	
296498	DIDmey	Diderma	Diderma meyerae	no sequence			2013	1657	11.07127	47.4523	
296513	DIDmey	Diderma	Diderma meyerae	no sequence			2013	1657	11.07142	47.4523	
296514	DIDmey	Diderma	Diderma meyerae	no sequence			2013	1657	11.07143	47.4523	
296547	DIDmey	Diderma	Diderma meyerae	no sequence			2013	1604	11.06999	47.45238	
28399	DIDmey	Diderma	Diderma meyerae	omitted			2015	1622	11.07998	47.45491	LT670261
28367	DIDmic	Diderma	Diderma microcarpum		68	10	2015	1602	11.06643	47.451	LT670234
28460	DIDmic	Diderma	Diderma microcarpum		68	10	2015	1891	11.05752	47.44286	LT670307
29102	DIDmic	Diderma	Diderma microcarpum		68	10	2016	1603	11.06289	47.44572	MG820027
28457	DIDmic	Diderma	Diderma microcarpum	no sequence			2015	1876	11.05733	47.44327	
28492	DIDmic	Diderma	Diderma microcarpum	no sequence			2015	1870	11.05725	47.44288	
28954	DIDmic	Diderma	Diderma microcarpum	no sequence			2016	1486	11.0772	47.45592	
29062	DIDmic	Diderma	Diderma microcarpum	no sequence			2016	1641	11.06619	47.45125	
29116	DIDmic	Diderma	Diderma microcarpum	no sequence			2016	1698	11.06203	47.44225	
28395	DIDniv	Diderma	Diderma niveum		28	11	2015	1473	11.08306	47.45638	LT670257
28420	DIDniv	Diderma	Diderma niveum		28	11	2015	1639	11.07387	47.4528	LT670278
28437	DIDniv	Diderma	Diderma niveum		28	11	2015	1639	11.07706	47.45313	LT670291
28463	DIDniv	Diderma	Diderma niveum		28	11	2015	1897	11.05756	47.44228	LT670310
28464	DIDniv	Diderma	Diderma niveum		28	11	2015	1895	11.05768	47.44234	LT670311
28469	DIDniv	Diderma	Diderma niveum		28	11	2015	1903	11.05735	47.44222	LT670315
28477	DIDniv	Diderma	Diderma niveum		28	11	2015	1598	11.06951	47.45243	LT670323
28491	DIDniv	Diderma	Diderma niveum		28	11	2015	1870	11.05728	47.44269	LT670333
29048	DIDniv	Diderma	Diderma niveum		28	11	2016	1556	11.07436	47.45442	MG819811
28470	DIDniv	Diderma	Diderma niveum		46	11	2015	1904	11.05736	47.44222	LT670316
28438	DIDniv	Diderma	Diderma niveum	no sequence			2015	1639	11.07706	47.45313	
28943	DIDniv	Diderma	Diderma niveum	no sequence			2016	1450	11.08248	47.457	
28944	DIDniv	Diderma	Diderma niveum	no sequence			2016	1450	11.08248	47.457	
29261	DIDniv	Diderma	Diderma niveum	no sequence			2016	1903	11.05713	47.44229	
29047	LAM	Lamproderma	Lamproderma sp.	no sequence			2016	1504	11.07419	47.45436	
29060	LAM	Lamproderma	Lamproderma sp.	no sequence			2016	1613	11.06667	47.45289	
29086	LAMaen	Lamproderma	Lamproderma aeneum	no sequence			2016	1653	11.06197	47.44581	
29203	LAMaen	Lamproderma	Lamproderma aeneum	no sequence			2016	1482	11.08567	47.45825	
29204	LAMaen	Lamproderma	Lamproderma aeneum	no sequence			2016	1482	11.08567	47.45825	
29205	LAMaen	Lamproderma	Lamproderma aeneum	no sequence			2016	1482	11.08567	47.45825	
29206	LAMaen	Lamproderma	Lamproderma aeneum	no sequence			2016	1482	11.08567	47.45825	
29207	LAMaen	Lamproderma	Lamproderma aeneum	no sequence			2016	1482	11.08567	47.45825	
29208	LAMaen	Lamproderma	Lamproderma aeneum	no sequence			2016	1482	11.08567	47.45825	
see 26282	LAMaen	Lamproderma	Lamproderma aeneum	no sequence			2013	1221	11.09679	47.46714	
26322	LAMaen-a	Lamproderma	Lamproderma aeneum		1	13	2013	1582	11.0713	47.45203	LT670100
26329	LAMaen-a	Lamproderma	Lamproderma aeneum		1	13	2013	1582	11.07138	47.45203	LT670105
26334	LAMaen-a	Lamproderma	Lamproderma aeneum		1	13	2013	1582	11.07143	47.45203	LT670110
26366	LAMaen-a	Lamproderma	Lamproderma aeneum		1	13	2013	1688	11.08083	47.4531	LT670133
28973	LAMaen-a	Lamproderma	Lamproderma aeneum		1	13	2016	1365	11.08428	47.46214	MG819890
26407	LAMaen-b	Lamproderma	Lamproderma aeneum		16	12	2013	1629	11.06597	47.44996	LT670159
28964	LAMaen-b	Lamproderma	Lamproderma aeneum		16	12	2016	1379	11.08297	47.46147	MG819910
28971	LAMaen-b	Lamproderma	Lamproderma aeneum		16	12	2016	1365	11.08428	47.46214	MG819770
26415	LAMaen-c	Lamproderma	Lamproderma aeneum		70	14	2013	1626	11.06312	47.44844	LT670164
28968	LAMaen-c	Lamproderma	Lamproderma aeneum		70	14	2016	1378	11.08486	47.46142	MG819888
28976	LAMaen-c	Lamproderma	Lamproderma aeneum		70	14	2016	1367	11.08283	47.46161	MG819748
29057	LAMaen-c	Lamproderma	Lamproderma aeneum		70	14	2016	1625	11.06686	47.45289	MG819889
29195	LAMaen-c	Lamproderma	Lamproderma aeneum		70	14	2016	1476	11.08486	47.45881	MG819749
26304	LAMaen-d	Lamproderma	Lamproderma aeneum		39	15	2013	1688	11.08099	47.4531	LT670090
28510	LAMaen-d	Lamproderma	Lamproderma aeneum		39	15	2015	1298	11.09631	47.465	LT670346
26315	LAMaen-e	Lamproderma	Lamproderma aeneum		56	16	2013	1684	11.07866	47.45309	LT670095
28436	LAMaen-e	Lamproderma	Lamproderma aeneum		56	16	2015	1725	11.07828	47.45229	LT670290
28445	LAMaen-e	Lamproderma	Lamproderma aeneum		56	16	2015	1508	11.08184	47.45667	LT670295
28490	LAMaen-e	Lamproderma	Lamproderma aeneum		56	16	2015	1870	11.05721	47.44326	LT670332
28524	LAMaen-e	Lamproderma	Lamproderma aeneum		56	16	2015	1300	11.09667	47.46442	LT670355
29007	LAMaen-e	Lamproderma	Lamproderma aeneum		56	16	2016	1692	11.07994	47.45244	MG819860
29035	LAMaen-e	Lamproderma	Lamproderma aeneum		56	16	2016	1605	11.08017	47.45406	MG820001
29072	LAMaen-e	Lamproderma	Lamproderma aeneum		56	16	2016	1612	11.06594	47.45039	MG819861
29076	LAMaen-e	Lamproderma	Lamproderma aeneum		56	16	2016	1611	11.06556	47.44983	MG820002
29090	LAMaen-e	Lamproderma	Lamproderma aeneum		56	16	2016	1683	11.06281	47.44297	MG819862
29145	LAMaen-e	Lamproderma	Lamproderma aeneum		56	16	2016	1528	11.07425	47.45433	MG820003
26333	LAMalb	Lamproderma	Lamproderma album		37	17	2013	1582	11.07142	47.45203	LT670109
26381	LAMalb	Lamproderma	Lamproderma album		37	17	2013	1582	11.07121	47.45203	LT670143
26433	LAMalb	Lamproderma	Lamproderma album		37	17	2013	1893	11.05752	47.4424	LT670172
26448	LAMalb	Lamproderma	Lamproderma album		37	17	2013	1740	11.0607	47.44525	LT670179
26450	LAMalb	Lamproderma	Lamproderma album		37	17	2013	1740	11.06072	47.44525	LT670180

28447	LAMalb	Lamproderma	Lamproderma album		37	17	2015	1375	11.08419	47.46212	LT670297
28516	LAMalb	Lamproderma	Lamproderma album		37	17	2015	1298	11.09631	47.465	LT670350
28990	LAMalb	Lamproderma	Lamproderma album		37	17	2016	1328	11.09936	47.46328	MG819835
29050	LAMalb	Lamproderma	Lamproderma album		37	17	2016	1523	11.07417	47.45442	MG819976
29051	LAMalb	Lamproderma	Lamproderma album		37	17	2016	1523	11.07417	47.45442	MG819836
29118	LAMalb	Lamproderma	Lamproderma album		37	17	2016	1699	11.06206	47.44211	MG819977
29155	LAMalb	Lamproderma	Lamproderma album		37	17	2016	1580	11.07092	47.45178	MG819837
29175	LAMalb	Lamproderma	Lamproderma album		37	17	2016	1720	11.062	47.44436	MG819978
29210	LAMalb	Lamproderma	Lamproderma album		37	17	2016	1482	11.08567	47.45825	MG819838
26430	LAMalb	Lamproderma	Lamproderma album	no sequence			2013	1893	11.05749	47.4424	
26431	LAMalb	Lamproderma	Lamproderma album	no sequence			2013	1893	11.0575	47.4424	
26449	LAMalb	Lamproderma	Lamproderma album	no sequence			2013	1740	11.06071	47.44525	
28427	LAMalb	Lamproderma	Lamproderma album	no sequence			2015	1679	11.08027	47.4522	
28535	LAMalb	Lamproderma	Lamproderma album	no sequence			2015	1663	11.08076	47.45321	
26376	LAMarc	Lamproderma	Lamproderma arcyrioides		23	18	2013	1688	11.08095	47.4531	LT670139
28376	LAMarc	Lamproderma	Lamproderma arcyrioides		23	18	2015	1419	11.0859	47.46038	LT670243
29149	LAMarc	Lamproderma	Lamproderma arcyrioides		23	18	2016	1365	11.08108	47.46156	MG819940
28369	LAMarc	Lamproderma	Lamproderma arcyrioides		61	18	2015	1644	11.06607	47.45171	LT670236
29173	LAMarc	Lamproderma	Lamproderma arcyrioides		61	18	2016	1698	11.06136	47.44397	MG820011
28428	LAMarc	Lamproderma	Lamproderma arcyrioides	no sequence			2015	1679	11.08027	47.4522	
26409	LAMcrs	Lamproderma	Lamproderma cristatum		42	19	2013	1629	11.06599	47.44996	LT670160
26288	LAMesp	Lamproderma	Lamproderma echinosporum		15	20	2013	1277	11.09678	47.46714	LT670080
26300	LAMesp	Lamproderma	Lamproderma echinosporum		15	20	2013	1657	11.07156	47.4523	LT670087
26340	LAMesp	Lamproderma	Lamproderma echinosporum		15	20	2013	1684	11.07865	47.45309	LT670114
28361	LAMesp	Lamproderma	Lamproderma echinosporum		15	20	2015	1611	11.06572	47.44989	LT670229
28375	LAMesp	Lamproderma	Lamproderma echinosporum		15	20	2015	1419	11.08589	47.46038	LT670242
28406	LAMesp	Lamproderma	Lamproderma echinosporum		15	20	2015	1622	11.08005	47.45491	LT670268
28499	LAMesp	Lamproderma	Lamproderma echinosporum		15	20	2015	1278	11.10273	47.46424	LT670338
28532	LAMesp	Lamproderma	Lamproderma echinosporum		15	20	2015	1665	11.08067	47.45314	LT670358
29052	LAMesp	Lamproderma	Lamproderma echinosporum		15	20	2016	1523	11.07269	47.45325	MG819761
29053	LAMesp	Lamproderma	Lamproderma echinosporum		15	20	2016	1618	11.06783	47.45303	MG819902
29056	LAMesp	Lamproderma	Lamproderma echinosporum		15	20	2016	1625	11.06686	47.45289	MG819762
29073	LAMesp	Lamproderma	Lamproderma echinosporum		15	20	2016	1629	11.066	47.45019	MG819903
29093	LAMesp	Lamproderma	Lamproderma echinosporum		15	20	2016	1662	11.06169	47.44467	MG819763
29103	LAMesp	Lamproderma	Lamproderma echinosporum		15	20	2016	1603	11.06289	47.44572	MG819904
29104	LAMesp	Lamproderma	Lamproderma echinosporum		15	20	2016	1596	11.063	47.44506	MG819764
29109	LAMesp	Lamproderma	Lamproderma echinosporum		15	20	2016	1995	11.06258	47.44247	MG819905
29115	LAMesp	Lamproderma	Lamproderma echinosporum		15	20	2016	1698	11.06203	47.44225	MG819765
29123	LAMesp	Lamproderma	Lamproderma echinosporum		15	20	2016	1702	11.06169	47.44211	MG819906
29125	LAMesp	Lamproderma	Lamproderma echinosporum		15	20	2016	1702	11.06169	47.44211	MG819766
29128	LAMesp	Lamproderma	Lamproderma echinosporum		15	20	2016	1749	11.06125	47.44236	MG819907
29129	LAMesp	Lamproderma	Lamproderma echinosporum		15	20	2016	1749	11.06125	47.44236	MG819767
29130	LAMesp	Lamproderma	Lamproderma echinosporum		15	20	2016	1749	11.06125	47.44236	MG819908
29144	LAMesp	Lamproderma	Lamproderma echinosporum		15	20	2016	1528	11.07425	47.45433	MG819768
29176	LAMesp	Lamproderma	Lamproderma echinosporum		15	20	2016	1720	11.062	47.44436	MG819909
29238	LAMesp	Lamproderma	Lamproderma echinosporum		15	20	2016	1885	11.05718	47.44228	MG819769
26306	LAMesp	Lamproderma	Lamproderma echinosporum	no sequence			2013	1688	11.08101	47.4531	
26336	LAMesp	Lamproderma	Lamproderma echinosporum	no sequence			2013	1613	11.0702	47.45142	
26421	LAMesp	Lamproderma	Lamproderma echinosporum	no sequence			2013	1626	11.06318	47.44844	
26447	LAMesp	Lamproderma	Lamproderma echinosporum	no sequence			2013	1626	11.06329	47.44844	
28412	LAMesp	Lamproderma	Lamproderma echinosporum	no sequence			2015	1600	11.07988	47.45462	
26309	LAMovo	Lamproderma	Lamproderma ovoideum	no sequence			2013	1699	11.08033	47.45268	
26313	LAMovo	Lamproderma	Lamproderma ovoideum	no sequence			2013	1684	11.07863	47.45309	
26314	LAMovo	Lamproderma	Lamproderma ovoideum	no sequence			2013	1684	11.07864	47.45309	
26380	LAMovo	Lamproderma	Lamproderma ovoideum	no sequence			2013	1582	11.0712	47.45203	
26385	LAMovo	Lamproderma	Lamproderma ovoideum	no sequence			2013	1582	11.07126	47.45203	
26432	LAMovo	Lamproderma	Lamproderma ovoideum	no sequence			2013	1893	11.05751	47.4424	
26435	LAMovo	Lamproderma	Lamproderma ovoideum	no sequence			2013	1893	11.05754	47.4424	
28371	LAMovo	Lamproderma	Lamproderma ovoideum	no sequence			2015	1386	11.08599	47.46084	
28393	LAMovo	Lamproderma	Lamproderma ovoideum	no sequence			2015	1503	11.08291	47.4567	
28419	LAMovo	Lamproderma	Lamproderma ovoideum	no sequence			2015	1639	11.07386	47.4528	
28935	LAMovo	Lamproderma	Lamproderma ovoideum	no sequence			2016	1277	11.10289	47.46422	
28941	LAMovo	Lamproderma	Lamproderma ovoideum	no sequence			2016	1348	11.0852	47.46175	
28942	LAMovo	Lamproderma	Lamproderma ovoideum	no sequence			2016	1466	11.08247	47.457	
29069	LAMovo	Lamproderma	Lamproderma ovoideum	no sequence			2016	1612	11.06594	47.45039	
29081	LAMovo	Lamproderma	Lamproderma ovoideum	no sequence			2016	1611	11.06433	47.44869	
29105	LAMovo	Lamproderma	Lamproderma ovoideum	no sequence			2016	1596	11.063	47.44506	
29191	LAMovo	Lamproderma	Lamproderma ovoideum	no sequence			2016	1476	11.08486	47.45881	
29196	LAMovo	Lamproderma	Lamproderma ovoideum	no sequence			2016	1476	11.08486	47.45881	
29197	LAMovo	Lamproderma	Lamproderma ovoideum	no sequence			2016	1482	11.08567	47.45825	
29199	LAMovo	Lamproderma	Lamproderma ovoideum	no sequence			2016	1482	11.08567	47.45825	
29200	LAMovo	Lamproderma	Lamproderma ovoideum	no sequence			2016	1482	11.08567	47.45825	
29201	LAMovo	Lamproderma	Lamproderma ovoideum	no sequence			2016	1482	11.08567	47.45825	
29264	LAMovo	Lamproderma	Lamproderma ovoideum	no sequence			2016	1903	11.05713	47.44229	
296522	LAMovo	Lamproderma	Lamproderma ovoideum	no sequence			2013	1688	11.08087	47.4531	
296553	LAMovo	Lamproderma	Lamproderma ovoideum	no sequence			2013	1605	11.07089	47.45269	
296608	LAMovo	Lamproderma	Lamproderma ovoideum	no sequence			2013	1629	11.06603	47.44996	
296618	LAMovo	Lamproderma	Lamproderma ovoideum	no sequence			2013	1604	11.06999	47.45238	
see28542	LAMovo	Lamproderma	Lamproderma ovoideum	no sequence			2015	1588	11.07969	47.45473	
26297	LAMovo_a	Lamproderma	Lamproderma ovoideum		20	23	2013	1657	11.07153	47.4523	LT670085
26365	LAMovo_a	Lamproderma	Lamproderma ovoideum		20	23	2013	1688	11.08082	47.4531	LT670132
26393	LAMovo_a	Lamproderma	Lamproderma ovoideum		20	23	2013	1605	11.07086	47.45269	LT670153
26416	LAMovo_a	Lamproderma	Lamproderma ovoideum		20	23	2013	1626	11.06314	47.44844	LT670165
28359	LAMovo_a	Lamproderma	Lamproderma ovoideum		20	23	2015	1640	11.0656	47.44946	LT670227
28366	LAMovo_a	Lamproderma	Lamproderma ovoideum		20	23	2015	1602	11.06642	47.451	LT670233
28368	LAMovo_a	Lamproderma	Lamproderma ovoideum		20	23	2015	1611	11.0661	47.45125	LT670235
28373	LAMovo_a	Lamproderma	Lamproderma ovoideum		20	23	2015	1394	11.08609	47.4605	LT670240
28379	LAMovo_a	Lamproderma	Lamproderma ovoideum		20	23	2015	1427	11.08558	47.46001	LT670245
28383	LAMovo_a	Lamproderma	Lamproderma ovoideum		20	23	2015	1366	11.08416	47.46208	LT670249
28386	LAMovo_a	Lamproderma	Lamproderma ovoideum		20	23	2015	1345	11.08387	47.46215	LT670252
28409	LAMovo_a	Lamproderma	Lamproderma ovoideum		20	23	2015	1600	11.07985	47.45462	MG819747
28416	LAMovo_a	Lamproderma	Lamproderma ovoideum		20	23	2015	1677	11.07861	47.45442	LT670274
28422	LAMovo_a	Lamproderma	Lamproderma ovoideum		20	23	2015	1646	11.07401	47.4526	LT670280
28449	LAMovo_a	Lamproderma	Lamproderma ovoideum		20	23	2015	1375	11.08421	47.46212	LT670299
28451	LAMovo_a	Lamproderma	Lamproderma ovoideum		20	23	2015	1375	11.08423	47.46212	LT670301
28497	LAMovo_a	Lamproderma	Lamproderma ovoideum		20	23	2015	1203	11.10582	47.46267	LT670336
28501	LAMovo_a	Lamproderma	Lamproderma ovoideum		20	23	2015	1278	11.10273	47.46424	LT670340
28502	LAMovo_a	Lamproderma	Lamproderma ovoideum		20	23	2015	1278	11.10273	47.46424	LT670341
28544	LAMovo_a	Lamproderma	Lamproderma ovoideum		20	23	2015	1588	11.07969	47.45473	LT670368
28951	LAMovo_a	Lamproderma	Lamproderma ovoideum		20	23	2016	1455	11.07754	47.45607	MG819774
28952	LAMovo_a	Lamproderma	Lamproderma ovoideum		20	23	2016	1455	11.07754	47.45607	MG819915
28961	LAMovo_a	Lamproderma	Lamproderma ovoideum		20	23	2016	1379	11.08297	47.46147	MG819775
28970	LAMovo_a	Lamproderma	Lamproderma ovoideum		20	23	2016	1365	11.08428	47.46214	MG819916
28975	LAMovo_a	Lamproderma	Lamproderma ovoideum		20	23	2016	1365	11.08428	47.46214	MG819776
29001	LAMovo_a	Lamproderma	Lamproderma ovoideum		20	23	2016	1568	11.08308	47.45553	MG819917
29003	LAMovo_a	Lamproderma	Lamproderma ovoideum		20	23	2016	1568	11.08308	47.45553	MG819777
29020	LAMovo_a	Lamproderma	Lamproderma ovoideum		20	23	2016	1553	11.07944	47.45494	MG819918
29040	LAMovo_a	Lamproderma	Lamproderma ovoideum		20	23	2016	1588	11.08011	47.45389	MG819778
29064	LAMovo_a	Lamproderma	Lamproderma ovoideum		20	23	2016	1652	11.06639	47.45119	MG819919
29067	LAMovo_a	Lamproderma	Lamproderma ovoideum		20	23	2016	1650	11.06617	47.451	MG819779
29092	LAMovo_a	Lamproderma	Lamproderma ovoideum		20	23	2016	1662	11.06169	47.44467	MG819920
29095	LAMovo_a	Lamproderma	Lamproderma ovoideum		20	23	2016	1662	11.06169	47.44467	MG819780
29167	LAMovo_a	Lamproderma	Lamproderma ovoideum		20	23	2016	1691	11.06175	47.44433	MG819921
29169	LAMovo_a	Lamproderma	Lamproderma ovoideum		20	23	2016	1691	11.06175	47.44433	MG819781
26383	LAMovo-b	Lamproderma	Lamproderma ovoideum		21	24	2013	1582	11.07123	47.45203	LT670145
26386	LAMovo-b	Lamproderma	Lamproderma ovoideum		21	24	2013	1582	11.07127	47.45203	LT670147
26439	LAMovo-b	Lamproderma	Lamproderma ovoideum		21	24	2013	1604	11.06996	47.45238	LT670175

28358	LAMovo-b	Lamproderma	Lamproderma ovoideum		21	24	2015	1640	11.06559	47.44946	LT670226
28363	LAMovo-b	Lamproderma	Lamproderma ovoideum		21	24	2015	1609	11.06555	47.44993	LT670231
28511	LAMovo-b	Lamproderma	Lamproderma ovoideum		21	24	2015	1298	11.09631	47.465	LT670347
28947	LAMovo-b	Lamproderma	Lamproderma ovoideum		21	24	2016	1455	11.07754	47.45607	MG819922
28953	LAMovo-b	Lamproderma	Lamproderma ovoideum		21	24	2016	1455	11.07754	47.45607	MG819782
28969	LAMovo-b	Lamproderma	Lamproderma ovoideum		21	24	2016	1378	11.08486	47.46142	MG819923
29032	LAMovo-b	Lamproderma	Lamproderma ovoideum		21	24	2016	1626	11.08031	47.45433	MG819783
29045	LAMovo-b	Lamproderma	Lamproderma ovoideum		21	24	2016	1504	11.07419	47.45436	MG819924
29112	LAMovo-b	Lamproderma	Lamproderma ovoideum		21	24	2016	1995	11.06258	47.44247	MG819784
29193	LAMovo-b	Lamproderma	Lamproderma ovoideum		21	24	2016	1476	11.08486	47.45881	MG819925
26338	LAMovo-c	Lamproderma	Lamproderma ovoideum		2	25	2013	1605	11.07082	47.45269	LT670113
26391	LAMovo-c	Lamproderma	Lamproderma ovoideum		2	25	2013	1605	11.07084	47.45269	LT670151
26392	LAMovo-c	Lamproderma	Lamproderma ovoideum		2	25	2013	1605	11.07085	47.45269	LT670152
26394	LAMovo-c	Lamproderma	Lamproderma ovoideum		2	25	2013	1605	11.07087	47.45269	LT670154
26395	LAMovo-c	Lamproderma	Lamproderma ovoideum		2	25	2013	1605	11.07088	47.45269	LT670155
26326	LAMovo-c	Lamproderma	Lamproderma ovoideum		17	25	2013	1582	11.07134	47.45203	LT670103
29188	LAMovo-d	Lamproderma	Lamproderma ovoideum		3	21	2016	1795	11.05756	47.44036	MG819750
29000	LAMovo-d	Lamproderma	Lamproderma ovoideum		4	21	2016	1522	11.08292	47.45628	MG819891
26342	LAMovo-d	Lamproderma	Lamproderma ovoideum		18	21	2013	1582	11.07137	47.45203	LT670116
28405	LAMovo-d	Lamproderma	Lamproderma ovoideum		18	21	2015	1622	11.08004	47.45491	LT670267
28487	LAMovo-d	Lamproderma	Lamproderma ovoideum		18	21	2015	1870	11.05717	47.44295	LT670329
28489	LAMovo-d	Lamproderma	Lamproderma ovoideum		18	21	2015	1870	11.05721	47.44294	LT670331
28949	LAMovo-d	Lamproderma	Lamproderma ovoideum		18	21	2016	1460	11.07661	47.45539	MG819911
28955	LAMovo-d	Lamproderma	Lamproderma ovoideum		18	21	2016	1486	11.0772	47.45592	MG819771
28985	LAMovo-d	Lamproderma	Lamproderma ovoideum		18	21	2016	1323	11.09692	47.46325	MG819912
28988	LAMovo-d	Lamproderma	Lamproderma ovoideum		18	21	2016	1312	11.09825	47.46319	MG819772
28996	LAMovo-d	Lamproderma	Lamproderma ovoideum		18	21	2016	1498	11.08353	47.45658	MG819913
28997	LAMovo-d	Lamproderma	Lamproderma ovoideum		18	21	2016	1498	11.08353	47.45658	MG819773
28414	LAMovo-d	Lamproderma	Lamproderma ovoideum		19	21	2015	1635	11.08027	47.45459	LT670272
29202	LAMovo-d	Lamproderma	Lamproderma ovoideum		19	21	2016	1482	11.08567	47.45825	MG819914
26357	LAMovo-d	Lamproderma	Lamproderma ovoideum		40	21	2013	1657	11.07135	47.4523	LT670125
26371	LAMovo-d	Lamproderma	Lamproderma ovoideum		40	21	2013	1688	11.0809	47.4531	LT670136
26412	LAMovo-d	Lamproderma	Lamproderma ovoideum		40	21	2013	1629	11.06602	47.44996	LT670162
28355	LAMovo-d	Lamproderma	Lamproderma ovoideum		40	21	2015	1453	11.08363	47.45831	LT670224
28370	LAMovo-d	Lamproderma	Lamproderma ovoideum		40	21	2015	1644	11.06608	47.45171	LT670237
28446	LAMovo-d	Lamproderma	Lamproderma ovoideum		40	21	2015	1508	11.08185	47.45667	LT670296
28520	LAMovo-d	Lamproderma	Lamproderma ovoideum		40	21	2015	1298	11.09631	47.465	LT670353
28987	LAMovo-d	Lamproderma	Lamproderma ovoideum		40	21	2016	1312	11.09825	47.46319	MG819839
28992	LAMovo-d	Lamproderma	Lamproderma ovoideum		40	21	2016	1475	11.08367	47.4565	MG819980
28998	LAMovo-d	Lamproderma	Lamproderma ovoideum		40	21	2016	1519	11.08344	47.45639	MG819840
28999	LAMovo-d	Lamproderma	Lamproderma ovoideum		40	21	2016	1519	11.08344	47.45639	MG819981
29106	LAMovo-d	Lamproderma	Lamproderma ovoideum		40	21	2016	1596	11.063	47.44506	MG819841
29113	LAMovo-d	Lamproderma	Lamproderma ovoideum		40	21	2016	1995	11.06258	47.44247	MG819982
29147	LAMovo-d	Lamproderma	Lamproderma ovoideum		40	21	2016	1528	11.07425	47.45433	MG819842
28989	LAMovo-e	Lamproderma	Lamproderma ovoideum		57	22	2016	1312	11.09825	47.46319	MG819863
28440	LAMpir	Lamproderma	Lamproderma piriforme		9	26	2015	1484	11.08193	47.45679	LT670292
29089	LAMpul	Lamproderma	Lamproderma pulchellum		60	27	2016	1683	11.06281	47.44297	MG819870
28432	LAMrrs	Lamproderma	Lamproderma retirugisporum		38	28	2015	1629	11.08054	47.4528	LT670286
29065	LAMrrs	Lamproderma	Lamproderma retirugisporum		38	28	2016	1650	11.06617	47.451	MG819979
26299	LAMrrs	Lamproderma	Lamproderma retirugisporum	no sequence			2013	1657	11.07155	47.4523	
26287	LAMsau	Lamproderma	Lamproderma sauteri	no sequence			2013	1277	11.09677	47.46714	
26328	LAMsau	Lamproderma	Lamproderma sauteri	no sequence			2013	1582	11.07136	47.45203	
26408	LAMsau	Lamproderma	Lamproderma sauteri	no sequence			2013	1629	11.06598	47.44996	
26434	LAMsau	Lamproderma	Lamproderma sauteri	no sequence			2013	1893	11.05753	47.4424	
28389	LAMsau	Lamproderma	Lamproderma sauteri	no sequence			2015	1369	11.08271	47.46198	
28411	LAMsau	Lamproderma	Lamproderma sauteri	no sequence			2015	1600	11.07987	47.45462	
28439	LAMsau	Lamproderma	Lamproderma sauteri	no sequence			2015	1298	11.10282	47.46416	
28505	LAMsau	Lamproderma	Lamproderma sauteri	no sequence			2015	1298	11.09631	47.465	
28936	LAMsau	Lamproderma	Lamproderma sauteri	no sequence			2016	1277	11.1029	47.46422	
28937	LAMsau	Lamproderma	Lamproderma sauteri	no sequence			2016	1372	11.08417	47.4621	
28938	LAMsau	Lamproderma	Lamproderma sauteri	no sequence			2016	1375	11.08418	47.4621	
28939	LAMsau	Lamproderma	Lamproderma sauteri	no sequence			2016	1375	11.08419	47.4621	
28940	LAMsau	Lamproderma	Lamproderma sauteri	no sequence			2016	1348	11.08519	47.46175	
28945	LAMsau	Lamproderma	Lamproderma sauteri	no sequence			2016	1364	11.08417	47.4621	
28957	LAMsau	Lamproderma	Lamproderma sauteri	no sequence			2016	1379	11.08297	47.46147	
28965	LAMsau	Lamproderma	Lamproderma sauteri	no sequence			2016	1379	11.08297	47.46147	
28977	LAMsau	Lamproderma	Lamproderma sauteri	no sequence			2016	1358	11.08089	47.46167	
29041	LAMsau	Lamproderma	Lamproderma sauteri	no sequence			2016	1550	11.0815	47.45481	
29080	LAMsau	Lamproderma	Lamproderma sauteri	no sequence			2016	1590	11.06419	47.44925	
29211	LAMsau	Lamproderma	Lamproderma sauteri	no sequence			2016	1482	11.08567	47.45825	
see 28981	LAMsau	Lamproderma	Lamproderma sauteri	no sequence			2016	1345	11.08158	47.46206	
26436	LAMsau-a	Lamproderma	Lamproderma sauteri		59	32	2013	1893	11.05755	47.4424	LT670174
26411	LAMsau-b	Lamproderma	Lamproderma sauteri		6	29	2013	1629	11.06601	47.44996	LT670161
26443	LAMsau-b	Lamproderma	Lamproderma sauteri		6	29	2013	1626	11.06325	47.44844	LT670177
28365	LAMsau-b	Lamproderma	Lamproderma sauteri		6	29	2015	1602	11.06641	47.451	LT670232
28387	LAMsau-b	Lamproderma	Lamproderma sauteri		6	29	2015	1385	11.08367	47.46201	LT670253
28518	LAMsau-b	Lamproderma	Lamproderma sauteri		6	29	2015	1298	11.09631	47.465	LT670351
28978	LAMsau-b	Lamproderma	Lamproderma sauteri		6	29	2016	1358	11.08089	47.46167	MG819752
28982	LAMsau-b	Lamproderma	Lamproderma sauteri		6	29	2016	1345	11.08158	47.46206	MG819893
29055	LAMsau-b	Lamproderma	Lamproderma sauteri		6	29	2016	1625	11.06686	47.45289	MG819753
29077	LAMsau-b	Lamproderma	Lamproderma sauteri		6	29	2016	1611	11.06556	47.44983	MG819894
29151	LAMsau-b	Lamproderma	Lamproderma sauteri		6	29	2016	1365	11.08108	47.46156	MG819754
29153	LAMsau-b	Lamproderma	Lamproderma sauteri		6	29	2016	1365	11.08108	47.46156	MG819895
29154	LAMsau-b	Lamproderma	Lamproderma sauteri		6	29	2016	1365	11.08108	47.46156	MG819755
26324	LAMsau-b	Lamproderma	Lamproderma sauteri		22	29	2013	1582	11.07132	47.45203	LT670102
28351	LAMsau-b	Lamproderma	Lamproderma sauteri		22	29	2015	1430	11.08153	47.45749	LT670220
28353	LAMsau-b	Lamproderma	Lamproderma sauteri		22	29	2015	1430	11.08155	47.45749	LT670222
28372	LAMsau-b	Lamproderma	Lamproderma sauteri		22	29	2015	1386	11.086	47.46084	LT670239
28374	LAMsau-b	Lamproderma	Lamproderma sauteri		22	29	2015	1412	11.08597	47.46033	LT670241
28377	LAMsau-b	Lamproderma	Lamproderma sauteri		22	29	2015	1419	11.08591	47.46038	LT670244
28380	LAMsau-b	Lamproderma	Lamproderma sauteri		22	29	2015	1441	11.08553	47.45921	LT670246
28382	LAMsau-b	Lamproderma	Lamproderma sauteri		22	29	2015	1366	11.08415	47.46208	LT670248
28384	LAMsau-b	Lamproderma	Lamproderma sauteri		22	29	2015	1366	11.08417	47.46208	LT670250
28385	LAMsau-b	Lamproderma	Lamproderma sauteri		22	29	2015	1338	11.08349	47.46208	LT670251
28388	LAMsau-b	Lamproderma	Lamproderma sauteri		22	29	2015	1385	11.08368	47.46201	LT670254
28390	LAMsau-b	Lamproderma	Lamproderma sauteri		22	29	2015	1368	11.08508	47.46193	LT670255
28430	LAMsau-b	Lamproderma	Lamproderma sauteri		22	29	2015	1629	11.08054	47.4528	LT670284
28453	LAMsau-b	Lamproderma	Lamproderma sauteri		22	29	2015	1374	11.08266	47.46185	LT670302
28506	LAMsau-b	Lamproderma	Lamproderma sauteri		22	29	2015	1298	11.09631	47.465	LT670342
28508	LAMsau-b	Lamproderma	Lamproderma sauteri		22	29	2015	1298	11.09631	47.465	LT670344
28521	LAMsau-b	Lamproderma	Lamproderma sauteri		22	29	2015	1288	11.09633	47.46492	LT670354
28546	LAMsau-b	Lamproderma	Lamproderma sauteri		22	29	2015	1588	11.07969	47.45473	LT670370
28956	LAMsau-b	Lamproderma	Lamproderma sauteri		22	29	2016	1379	11.08297	47.46147	MG819785
28958	LAMsau-b	Lamproderma	Lamproderma sauteri		22	29	2016	1379	11.08297	47.46147	MG819926
28959	LAMsau-b	Lamproderma	Lamproderma sauteri		22	29	2016	1379	11.08297	47.46147	MG819786
28960	LAMsau-b	Lamproderma	Lamproderma sauteri		22	29	2016	1379	11.08297	47.46147	MG819927
28963	LAMsau-b	Lamproderma	Lamproderma sauteri		22	29	2016	1379	11.08297	47.46147	MG819787
28967	LAMsau-b	Lamproderma	Lamproderma sauteri		22	29	2016	1379	11.08297	47.46147	MG819928
28972	LAMsau-b	Lamproderma	Lamproderma sauteri		22	29	2016	1365	11.08428	47.46214	MG819788
28974	LAMsau-b	Lamproderma	Lamproderma sauteri		22	29	2016	1365	11.08428	47.46214	MG819929
28980	LAMsau-b	Lamproderma	Lamproderma sauteri		22	29	2016	1345	11.08158	47.46206	MG819789
28983	LAMsau-b	Lamproderma	Lamproderma sauteri		22	29	2016	1345	11.08158	47.46206	MG819930
28984	LAMsau-b	Lamproderma	Lamproderma sauteri		22	29	2016	1345	11.08158	47.46206	MG819790
28986	LAMsau-b	Lamproderma	Lamproderma sauteri		22	29	2016	1323	11.09692	47.46325	MG819931
28991	LAMsau-b	Lamproderma	Lamproderma sauteri		22	29	2016	1282	11.10225	47.46428	MG819791
29004	LAMsau-b	Lamproderma	Lamproderma sauteri		22	29	2016	1571	11.08328	47.45558	MG819932
29006	LAMsau-b	Lamproderma	Lamproderma sauteri		22	29	2016	1692	11.07994	47.45244	MG819792

29008	LAMsau-b	Lamproderma	Lamproderma sauteri		22	29	2016	1692	11.07994	47.45244	MG819933
29009	LAMsau-b	Lamproderma	Lamproderma sauteri		22	29	2016	1692	11.07994	47.45244	MG819793
29015	LAMsau-b	Lamproderma	Lamproderma sauteri		22	29	2016	1694	11.07972	47.45233	MG819934
29016	LAMsau-b	Lamproderma	Lamproderma sauteri		22	29	2016	1694	11.07972	47.45233	MG819794
29017	LAMsau-b	Lamproderma	Lamproderma sauteri		22	29	2016	1694	11.07972	47.45233	MG819935
29039	LAMsau-b	Lamproderma	Lamproderma sauteri		22	29	2016	1605	11.08017	47.45406	MG819795
29042	LAMsau-b	Lamproderma	Lamproderma sauteri		22	29	2016	1550	11.0815	47.45481	MG819936
29075	LAMsau-b	Lamproderma	Lamproderma sauteri		22	29	2016	1611	11.06556	47.44983	MG819796
29083	LAMsau-b	Lamproderma	Lamproderma sauteri		22	29	2016	1633	11.06225	47.44611	MG819937
29111	LAMsau-b	Lamproderma	Lamproderma sauteri		22	29	2016	1995	11.06258	47.44247	MG819797
29148	LAMsau-b	Lamproderma	Lamproderma sauteri		22	29	2016	1365	11.08108	47.46156	MG819938
29150	LAMsau-b	Lamproderma	Lamproderma sauteri		22	29	2016	1365	11.08108	47.46156	MG819798
29152	LAMsau-b	Lamproderma	Lamproderma sauteri		22	29	2016	1365	11.08108	47.46156	MG819939
29194	LAMsau-b	Lamproderma	Lamproderma sauteri		22	29	2016	1476	11.08486	47.45881	MG819799
28402	LAMsau-c	Lamproderma	Lamproderma sauteri		58	30	2015	1622	11.08001	47.45491	LT670264
28408	LAMsau-c	Lamproderma	Lamproderma sauteri		58	30	2015	1600	11.07984	47.45462	LT670269
28948	LAMsau-c	Lamproderma	Lamproderma sauteri		58	30	2016	1460	11.07661	47.45539	MG820004
28950	LAMsau-c	Lamproderma	Lamproderma sauteri		58	30	2016	1460	11.07661	47.45539	MG819864
28993	LAMsau-c	Lamproderma	Lamproderma sauteri		58	30	2016	1475	11.08367	47.4565	MG820005
28995	LAMsau-c	Lamproderma	Lamproderma sauteri		58	30	2016	1475	11.08367	47.4565	MG819865
29005	LAMsau-c	Lamproderma	Lamproderma sauteri		58	30	2016	1677	11.08044	47.4525	MG820006
29023	LAMsau-c	Lamproderma	Lamproderma sauteri		58	30	2016	1636	11.08006	47.45503	MG819866
29024	LAMsau-c	Lamproderma	Lamproderma sauteri		58	30	2016	1636	11.08006	47.45503	MG820007
29121	LAMsau-c	Lamproderma	Lamproderma sauteri		58	30	2016	1697	11.06181	47.44228	MG819867
29131	LAMsau-c	Lamproderma	Lamproderma sauteri		58	30	2016	1749	11.06125	47.44236	MG820008
29157	LAMsau-c	Lamproderma	Lamproderma sauteri		58	30	2016	1592	11.06847	47.45175	MG819868
29160	LAMsau-c	Lamproderma	Lamproderma sauteri		58	30	2016	1668	11.06156	47.44436	MG820009
29164	LAMsau-c	Lamproderma	Lamproderma sauteri		58	30	2016	1691	11.06175	47.44433	MG819869
29168	LAMsau-c	Lamproderma	Lamproderma sauteri		58	30	2016	1691	11.06175	47.44433	MG820010
26350	LAMsau-d	Lamproderma	Lamproderma sauteri		41	31	2013	1657	11.07124	47.4523	LT670120
28356	LAMsau-d	Lamproderma	Lamproderma sauteri		41	31	2015	1453	11.08364	47.45831	LT670225
28360	LAMsau-d	Lamproderma	Lamproderma sauteri		41	31	2015	1640	11.06561	47.44946	LT670228
28415	LAMsau-d	Lamproderma	Lamproderma sauteri		41	31	2015	1635	11.08028	47.45459	LT670273
28456	LAMsau-d	Lamproderma	Lamproderma sauteri		41	31	2015	1729	11.06097	47.44727	LT670304
28480	LAMsau-d	Lamproderma	Lamproderma sauteri		41	31	2015	1617	11.0711	47.45198	LT670326
28484	LAMsau-d	Lamproderma	Lamproderma sauteri		41	31	2015	1624	11.07124	47.45159	LT670328
28495	LAMsau-d	Lamproderma	Lamproderma sauteri		41	31	2015	1620	11.07106	47.45201	LT670335
28394	LAMzon	Lamproderma	Lamproderma zonatum		5	33	2015	1499	11.08303	47.45666	LT670256
29078	LAMzon	Lamproderma	Lamproderma zonatum		5	33	2016	1610	11.06464	47.44944	MG819751
29079	LAMzon	Lamproderma	Lamproderma zonatum		5	33	2016	1610	11.06464	47.44944	MG819892
26375	LEPcha	Lepidoderma	Lepidoderma chailletii	no sequence			2013	1688	11.08094	47.4531	
26422	LEPcha	Lepidoderma	Lepidoderma chailletii	no sequence			2013	1626	11.06319	47.44844	
28441	LEPcha	Lepidoderma	Lepidoderma chailletii	no sequence			2015	1484	11.08193	47.45679	
28537	LEPcha	Lepidoderma	Lepidoderma chailletii	no sequence			2015	1684	11.08104	47.45321	
29071	LEPcha	Lepidoderma	Lepidoderma chailletii	no sequence			2016	1612	11.06594	47.45039	
296490	LEPcha	Lepidoderma	Lepidoderma chailletii	no sequence			2013	1477	11.08731	47.45804	
26302	LEPcha-a	Lepidoderma	Lepidoderma chailletii		63	35	2013	1688	11.08097	47.4531	LT670089
28536	LEPcha-a	Lepidoderma	Lepidoderma chailletii		63	35	2015	1684	11.08104	47.45321	LT670361
29013	LEPcha-a	Lepidoderma	Lepidoderma chailletii		63	35	2016	1694	11.07972	47.45233	MG820012
29033	LEPcha-a	Lepidoderma	Lepidoderma chailletii		63	35	2016	1605	11.08017	47.45406	MG819872
29034	LEPcha-a	Lepidoderma	Lepidoderma chailletii		63	35	2016	1605	11.08017	47.45406	MG820013
29037	LEPcha-a	Lepidoderma	Lepidoderma chailletii		63	35	2016	1605	11.08017	47.45406	MG819873
29038	LEPcha-a	Lepidoderma	Lepidoderma chailletii		63	35	2016	1605	11.08017	47.45406	MG820014
29181	LEPcha-a	Lepidoderma	Lepidoderma chailletii		63	35	2016	1745	11.06006	47.4405	MG819874
29224	LEPcha-a	Lepidoderma	Lepidoderma chailletii		63	35	2016	1885	11.0712	47.4523	MG820015
29225	LEPcha-a	Lepidoderma	Lepidoderma chailletii		63	35	2016	1885	11.0712	47.4523	MG819875
29228	LEPcha-a	Lepidoderma	Lepidoderma chailletii		63	35	2016	1885	11.0712	47.4523	MG820016
29236	LEPcha-a	Lepidoderma	Lepidoderma chailletii		63	35	2016	1885	11.05753	47.44269	MG819876
29240	LEPcha-a	Lepidoderma	Lepidoderma chailletii		63	35	2016	1893	11.0712	47.4523	MG820017
29245	LEPcha-a	Lepidoderma	Lepidoderma chailletii		63	35	2016	1893	11.0712	47.4523	MG819877
29246	LEPcha-a	Lepidoderma	Lepidoderma chailletii		63	35	2016	1893	11.0712	47.4523	MG820018
29247	LEPcha-a	Lepidoderma	Lepidoderma chailletii		63	35	2016	1893	11.0712	47.4523	MG819878
29250	LEPcha-a	Lepidoderma	Lepidoderma chailletii		63	35	2016	1893	11.05741	47.44159	MG820019
28362	LEPcha-b	Lepidoderma	Lepidoderma chailletii		7	34	2015	1611	11.06573	47.44989	LT670230
28418	LEPcha-b	Lepidoderma	Lepidoderma chailletii		7	34	2015	1677	11.07863	47.45442	LT670276
28425	LEPcha-b	Lepidoderma	Lepidoderma chailletii		7	34	2015	1646	11.07401	47.4526	LT670282
28474	LEPcha-b	Lepidoderma	Lepidoderma chailletii		7	34	2015	1596	11.06948	47.45243	LT670320
28500	LEPcha-b	Lepidoderma	Lepidoderma chailletii		7	34	2015	1278	11.10273	47.46424	LT670339
29074	LEPcha-b	Lepidoderma	Lepidoderma chailletii		7	34	2016	1629	11.066	47.45019	MG819896
29231	LEPcha-b	Lepidoderma	Lepidoderma chailletii		7	34	2016	1885	11.05718	47.44228	MG819756
29232	LEPcha-b	Lepidoderma	Lepidoderma chailletii		7	34	2016	1885	11.05718	47.44228	MG819897
29233	LEPcha-b	Lepidoderma	Lepidoderma chailletii		7	34	2016	1885	11.05718	47.44228	MG819757
29248	LEPcha-b	Lepidoderma	Lepidoderma chailletii		7	34	2016	1893	11.05741	47.44159	MG819898
28442	LEPcha-b	Lepidoderma	Lepidoderma chailletii		44	34	2015	1503	11.08256	47.45617	LT670293
29059	LEPcha-b	Lepidoderma	Lepidoderma chailletii		62	34	2016	1613	11.06667	47.45289	MG819871
29190	MER	Meriderma	Meriderma sp.	no sequence			2016	1740	11.06039	47.44072	
see 28981	MER	Meriderma	Meriderma sp.	no sequence			2016	1345	11.08158	47.46206	
26296	MERagg	Meriderma	Meriderma aggregatum	no sequence			2013	1657	11.07152	47.4523	
28542	MERagg	Meriderma	Meriderma aggregatum	no sequence			2015	1588	11.07969	47.45473	
28547	MERagg	Meriderma	Meriderma aggregatum	no sequence			2015	1588	11.07969	47.45473	
28548	MERagg	Meriderma	Meriderma aggregatum	no sequence			2015	1588	11.07969	47.45473	
28550	MERagg	Meriderma	Meriderma aggregatum	no sequence			2015	1588	11.07969	47.45473	
28552	MERagg	Meriderma	Meriderma aggregatum	no sequence			2015	1616	11.08017	47.45477	
296504	MERagg	Meriderma	Meriderma aggregatum	no sequence			2013	1657	11.07133	47.4523	
296505	MERagg	Meriderma	Meriderma aggregatum	no sequence			2013	1657	11.07134	47.4523	
296538	MERagg	Meriderma	Meriderma aggregatum	no sequence			2013	1582	11.07124	47.45203	
26335	MERagg-a	Meriderma	Meriderma aggregatum		12	37	2013	1582	11.07144	47.45203	LT670111
28401	MERagg-a	Meriderma	Meriderma aggregatum		13	37	2015	1622	11.08	47.45491	LT670263
26330	MERagg-a	Meriderma	Meriderma aggregatum		34	37	2013	1582	11.07139	47.45203	LT670106
26382	MERagg-a	Meriderma	Meriderma aggregatum		34	37	2013	1582	11.07122	47.45203	LT670144
26384	MERagg-a	Meriderma	Meriderma aggregatum		34	37	2013	1582	11.07125	47.45203	LT670146
28397	MERagg-a	Meriderma	Meriderma aggregatum		34	37	2015	1622	11.07996	47.45491	LT670259
28404	MERagg-a	Meriderma	Meriderma aggregatum		34	37	2015	1622	11.08003	47.45491	LT670266
29025	MERagg-a	Meriderma	Meriderma aggregatum		34	37	2016	1636	11.08006	47.45503	MG819973
28398	MERagg-a	Meriderma	Meriderma aggregatum		55	37	2015	1622	11.07997	47.45491	LT670260
29097	MERagg-b	Meriderma	Meriderma aggregatum		52	36	2016	1662	11.06169	47.44467	MG819998
26343	MERcar	Meriderma	Meriderma carestiae	no sequence			2013	1061	11.09585	47.47091	
28452	MERcar-a	Meriderma	Meriderma carestiae		11	38	2015	1374	11.08265	47.46185	MG819899
29043	MERcar-a	Meriderma	Meriderma carestiae		11	38	2016	1550	11.0815	47.45481	MG819759
26344	MERcar-a	Meriderma	Meriderma carestiae		32	38	2013	1061	11.09586	47.47091	LT670117
28354	MERcar-a	Meriderma	Meriderma carestiae		53	38	2015	1453	11.08362	47.45831	LT670223
28994	MERcar-a	Meriderma	Meriderma carestiae		53	38	2016	1475	11.08367	47.4565	MG819858
29135	MERcar-a	Meriderma	Meriderma carestiae		53	38	2016	1738	11.06094	27.44261	MG819999
29158	MERcar-a	Meriderma	Meriderma carestiae		53	38	2016	1592	11.06847	47.45175	MG819859
28381	MERcar-a	Meriderma	Meriderma carestiae		69	38	2015	1303	11.0967	47.46435	LT670247
29192	MERcar-a	Meriderma	Meriderma carestiae		69	38	2016	1476	11.08486	47.45881	MG819887
29198	MERcar-a	Meriderma	Meriderma carestiae		69	38	2016	1482	11.08567	47.45825	MG820028
29209	MERcar-a	Meriderma	Meriderma carestiae		69	38	2016	1482	11.08567	47.45825	MG825330
28979	MERcar-b	Meriderma	Meriderma carestiae		33	39	2016	1345	11.08158	47.46206	MG819972
28981	MERcar-b	Meriderma	Meriderma carestiae		33	39	2016	1345	11.08158	47.46206	MG819832
28525	MERcarret	Meriderma	Meriderma carestiae var. retisporum	no sequence			2015	1585	11.07119	47.4523	
28448	MERcarret-a	Meriderma	Meriderma carestiae var. retisporum		32	38	2015	1375	11.0842	47.46212	LT670298
28450	MERcarret-b	Meriderma	Meriderma carestiae var. retisporum		33	39	2015	1375	11.08422	47.46212	LT670300
29163	MERech	Meriderma	Meriderma echinulatum		54	40	2016	1668	11.06156	47.44436	MG820000
28530	MERech	Meriderma	Meriderma echinulatum	no sequence			2015	1662	11.08065	47.45312	
26319	MERspi	Meriderma	Meriderma spinulisporum		31	41	2013	1701	11.07585	47.45269	LT670099
26341	MERspi	Meriderma	Meriderma spinulisporum		31	41	2013	1701	11.07586	47.45269	LT670115

26429	MERspi	Meriderma	Meriderma spinulisporum	no sequence			2013	1893	11.05748	47.4424	
28421	PHYalb	Physarum	Physarum albescens		30	43	2015	1629	11.07443	47.45251	LT670279
29172	PHYalb	Physarum	Physarum albescens		51	42	2016	1698	11.06136	47.44397	MG819857
29185	PHYals	Physarum	Physarum alpestre		10	44	2016	1797	11.05783	47.44042	MG819758
28435	PHYals	Physarum	Physarum alpestre		50	44	2015	1725	11.07828	47.45229	LT670289
29166	PHYals	Physarum	Physarum alpestre		50	44	2016	1691	11.06175	47.44433	MG819997
26284	PHYals	Physarum	Physarum alpestre	no sequence			2013	1221	11.09683	47.46714	
26303	PHYals	Physarum	Physarum alpestre	no sequence			2013	1688	11.08098	47.4531	
26307	PHYals	Physarum	Physarum alpestre	no sequence			2013	1699	11.08031	47.45268	
26321	PHYals	Physarum	Physarum alpestre	no sequence			2013	1582	11.07129	47.45203	
29124	PHYals	Physarum	Physarum alpestre	no sequence			2016	1702	11.06169	47.44211	
29177	PHYals	Physarum	Physarum alpestre	no sequence			2016	1779	11.06067	47.43886	
26337	PHYver	Physarum	Physarum vernum		14	45	2013	1604	11.06993	47.45238	LT670112
26374	PHYver	Physarum	Physarum vernum		14	45	2013	1688	11.08093	47.4531	LT670138
26401	PHYver	Physarum	Physarum vernum		14	45	2013	1604	11.06993	47.45238	LT670156
26404	PHYver	Physarum	Physarum vernum		14	45	2013	1604	11.06994	47.45238	LT670157
28417	PHYver	Physarum	Physarum vernum		14	45	2015	1677	11.07862	47.45442	LT670275
28426	PHYver	Physarum	Physarum vernum		14	45	2015	1651	11.07472	47.45243	LT670283
28475	PHYver	Physarum	Physarum vernum		14	45	2015	1597	11.06949	47.45243	LT670321
28476	PHYver	Physarum	Physarum vernum		14	45	2015	1598	11.0695	47.45243	LT670322
29180	PHYver	Physarum	Physarum vernum		14	45	2016	1745	11.06006	47.4405	MG819900
29183	PHYver	Physarum	Physarum vernum		14	45	2016	1741	11.06003	47.4405	MG819760
29189	PHYver	Physarum	Physarum vernum		14	45	2016	1740	11.06039	47.44072	MG819901
26280	PHYver	Physarum	Physarum vernum		35	45	2013	1221	11.09677	47.46714	LT670078
26310	PHYver	Physarum	Physarum vernum		35	45	2013	1699	11.08034	47.45268	LT670093
28512	PHYver	Physarum	Physarum vernum		35	45	2015	1298	11.09631	47.465	LT670348
28514	PHYver	Physarum	Physarum vernum		35	45	2015	1298	11.09631	47.465	LT670349
28528	PHYver	Physarum	Physarum vernum		35	45	2015	1657	11.07119	47.4523	LT670356
29091	PHYver	Physarum	Physarum vernum		35	45	2016	1683	11.06281	47.44297	MG819833
29096	PHYver	Physarum	Physarum vernum		35	45	2016	1662	11.06169	47.44467	MG819974
29143	PHYver	Physarum	Physarum vernum		35	45	2016	1675	11.08039	47.45317	MG819834
29182	PHYver	Physarum	Physarum vernum		35	45	2016	1745	11.06006	47.4405	MG819975
28479	PHYver	Physarum	Physarum vernum		36	45	2015	1617	11.07109	47.45198	LT670325
28515	PHYver	Physarum	Physarum vernum	no sequence			2015	1298	11.09631	47.465	
28529	PHYver	Physarum	Physarum vernum	no sequence			2015	1657	11.08034	47.45329	
26346	TRIalp	Trichia	Trichia alpina	no sequence			2013	1221	11.09681	47.46714	
26428	TRIalp	Trichia	Trichia alpina	no sequence			2013	1893	11.05747	47.4424	
29241	TRIsor	Trichia	Trichia sordida	no sequence			2016	1893	11.0712	47.4523	

S2

Supplementart data for: A four year survey reveals a coherent pattern between occurrence of fruit bodies and soil amoebae populations for nivicolous myxomycetes. M. Borg Dahl, O. Shchepin, C. Schunk, A. Menzel, Y. K. Novozhilov and M. Schnittler

Maximum Likelihood phylogenetic tree (500 bootstraps) of the 70 unique ribotypes fond within 533 sequenced specimens. Labels include a collection number to a reference specimen (Scxxxxx) of a reference specimen, species name, the code of the ribotype cluster (-a, -b, -c etc.) and the number of specimens per unique ribotype (seq #).

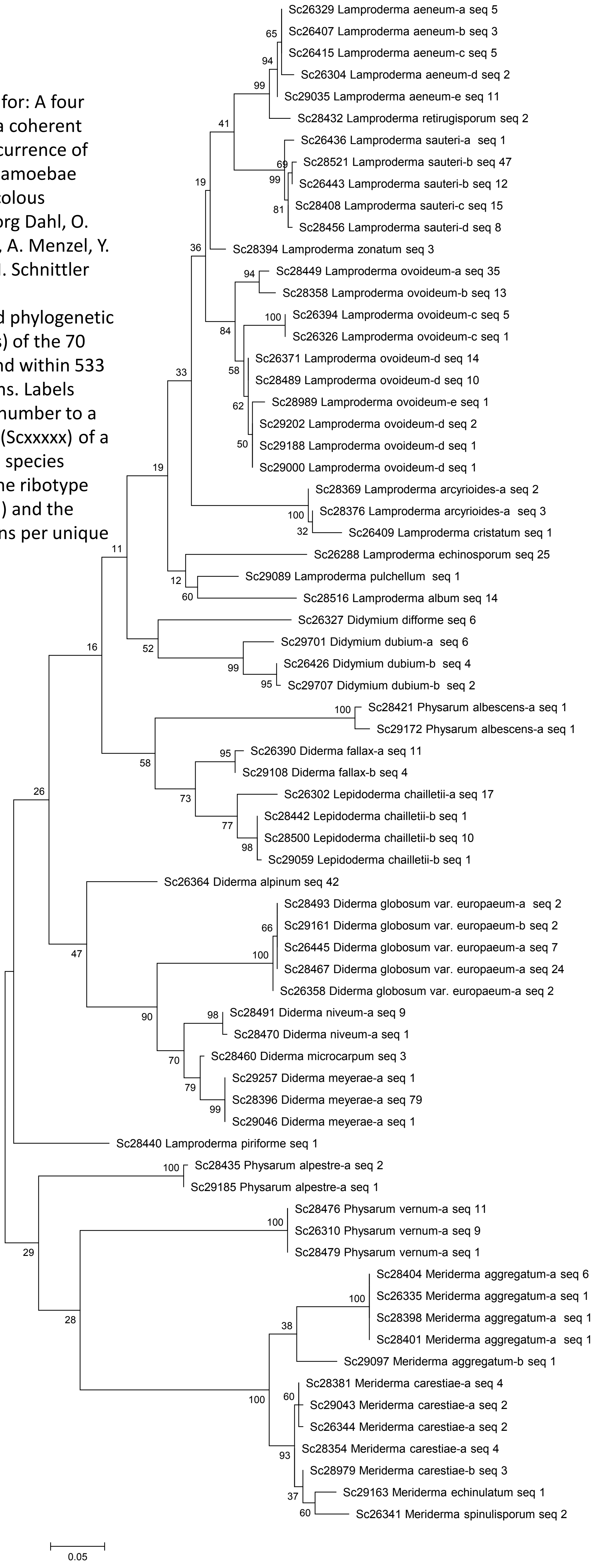

S3 – Supplementary data for: A four year survey reveals a coherent pattern between occurrence of fruit bodies and soil amoebae populations for nivicolous myxomycetes. M. Borg Dahl, O. Shchepin, C. Schunk, A. Menzel, Y. K. Novozhilov and M. Schnittler

OTUs accumulation curves

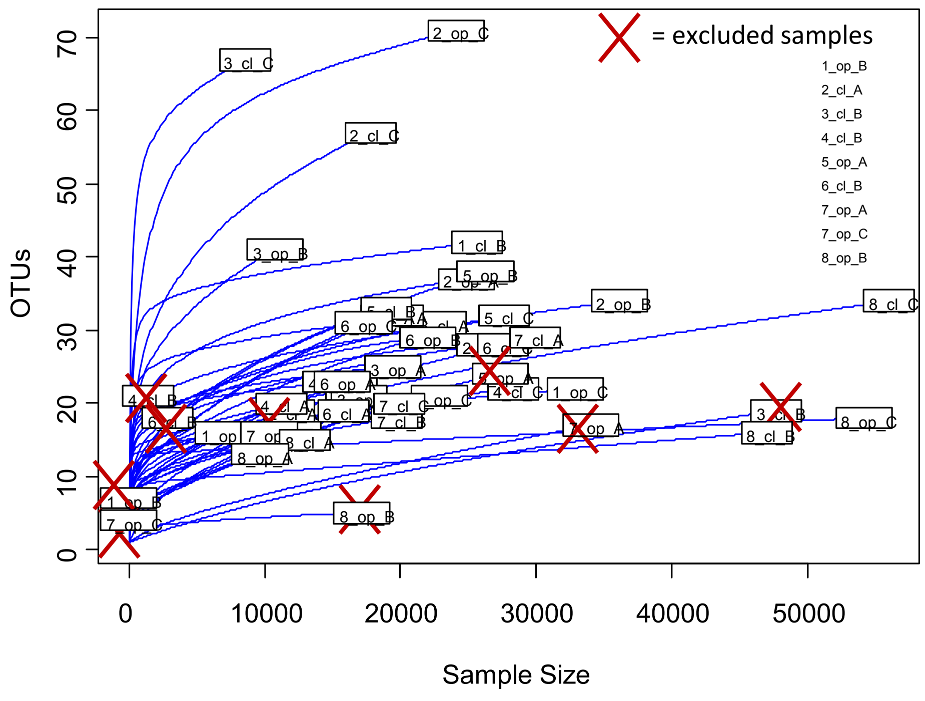

X Samples had either failed to amplify or not reach satuartion. See Borg Dahl et al. 2017 "**Fine scale niche differentiation among amoebal communities of dark-spored myxomycetes determined by landscape structures as well as biotic factors.**" (Submitted to *Environmental Microbiology* 30.12.2017)

Illumina sequencing quality - Q score distribution

ASCII	Q	Pe	N	Pct	AccPct
J	41	0.00008	1.8E+08	27.60%	27.60%
I	40	0.0001	138652	0.00%	27.60%
H	39	0.00013	2.01E+08	30.80%	58.40%
G	38	0.00016	1.54E+08	23.60%	82.00%
F	37	0.0002	49339953	7.60%	89.60%
E	36	0.00025	12275540	1.90%	91.50%
D	35	0.00032	4955128	0.80%	92.20%
C	34	0.0004	12107235	1.90%	94.10%
B	33	0.0005	12963024	2.00%	96.10%
A	32	0.00063	8132982	1.20%	97.30%
@	31	0.00079	1564297	0.20%	97.50%
?	30	0.001	2362195	0.40%	97.90%
>	29	0.00126	1253040	0.20%	98.10%
=	28	0.00158	120760	0.00%	98.10%
<	27	0.002	811099	0.10%	98.20%
;	26	0.00251	163940	0.00%	98.30%
:	25	0.00316	195812	0.00%	98.30%
…	…	…	…	…	…

Q score distribution (**Usearch manual)**
This section reports the number of bases found for each Q score.
Columns are: ASCII=symbol, Q=integer Phred score, N=number of bases,
Pct=number of bases with this Q score,
AccPct=number of bases with >= this Q score.

High Quality: >97% of all the Illumina base calls have 1/1800 (0.063%) chance of a miscall, Q-score=32), thus strict quality filtering (E_max= 0.25) could be applied in the OTU-picking pipeline.

Choosing the maximum expected error threshold (Usearch manual)
A natural choice is E_max = 1, because the most probable number of errors of a filtered read is zero.
Though of course, we expect some of them to have one or more errors
(this can't be entirely avoided, however stringent the filter, because Q scores are probabilities, not certainties).
If you want to filter even more stringently, you might choose something like E_max = 0.5 or E_max = 0.25.

S3 - Evaluation of Mock community

#OTU ID	taxa	Mock1	Mock2	Mock3	Average Mock	sd
OTU19	Diderma alpinum	1774	1560	1466	1600.0	158
OTU3	Diderma fallax	2525	2722	2512	2586.3	118
OTU155	Diderma globosum var. europaeum	2504	2359	2031	2298.0	242
OTU77	Diderma meyerae	1573	1496	1426	1498.3	74
OTU303	Didymium dubium	1258	1197	1056	1170.3	104
OTU2658	Lamproderma sp.	1807	1659	453	1306.3	743
OTU67	Lamproderma echinosporum	1812	1564	1489	1621.7	169
OTU175	Lamproderma sauteri	2388	2109	1977	2158.0	210
OTU238	Lepidoderma chailletii	1633	1440	1345	1472.7	147
OTU15	Lepidoderma chailletii	1569	1363	1285	1405.7	147
OTU2	Meriderma carestiae	1805	1399	1363	1522.3	245
OTU59	Meriderma spinulisporum	3929	3209	3125	3421.0	442
OTU9	Physarum albescens	1928	1812	1664	1801.3	132
OTU131	Physarum alpestre	2626	2594	2395	2538.3	125
OTU200	Physarum vernum	1916	1679	1598	1731.0	165
OTU4773	Didymium dubium	68	98	68		
OTU4521	unknown	59	58	28		
OTU69	Diacheopsis metallica	46	40	1027		
OTU3473	Physarum albescens	43	35	31		
OTU2512	Diderma alpinum	23	24	24		
OTU43	Diderma globosum	4	4			
OTU1645	Meriderma carestiae	1	4			
OTU25	Diderma niveum	1	1			
OTU54	CAU_RU	1				
OTU24	Didymium difforme	1				
OTU120	unknown	1				
OTU29	CAU_RU		1			
OTU35	CAU_RU		1			
OTU295	unknown			1		
OTU595	Lamproderma ovoideum			1		

Considered to be contamination, most likely introduced during mock preparation

Real Mock	col. No.	survey	abbreviation	species name
Sc28543_GAP15_DIDalp	Sc28543	GAP15	DIDalp	Diderma alpinum (Meylan) Meylan
Sc28478_GAP15_DIDfal	Sc28478	GAP15	DIDfal	Diderma fallax (Rostaf.) E. Sheld.
Sc26358_GAP13_DIDgloeur	Sc26358	GAP13	DIDgloeur	Diderma globosum var. europaeum Buyck
Sc28534_GAP15_DIDmey	Sc28534	GAP15	DIDmey	Diderma meyerae H. Singer, G. Moreno, Illana & A. Sánchez
Sc26426_GAP13_DDYdub	Sc26426	GAP13	DDYdub	Didymium dubium Rostaf.
Sc26409_GAP13_LAMcri*	Sc26409	GAP13	LAMcri	Lamproderma cribrarioides (Fr.) R.E. Fr.
Sc26288_GAP13_LAMesp	Sc26288	GAP13	LAMesp	Lamproderma echinosporum Meylan
Sc28508_GAP15_LAMsau	Sc28508	GAP15	LAMsau	Lamproderma sauteri Rostaf.
Sc28425_GAP15_LEPcha	Sc28425	GAP15	LEPcha_a	Lepidoderma chailletii a
Sc26302_GAP13_LEPcha	Sc26302	GAP13	LEPcha_b	Lepidoderma chailletii b
Sc26344_GAP13_MERcar	Sc26344	GAP13	MERcar	Meriderma carestiae (Ces. & de Not.) Mar. Mey. & Poulain
Sc26319_GAP13_MERspi	Sc26319	GAP13	MERspi	Meriderma spinulisporum ad int.
Sc28421_GAP15_PHYalb	Sc28421	GAP15	PHYalb	Physarum albescens Ellis ex T. Macbr.
Sc28435_GAP15_PHYals	Sc28435	GAP15	PHYals	Physarum alpestre Mitchell, Chapman & M.L. Farr
Sc26337_GAP13_PHYver	Sc26337	GAP13	PHYver	Physarum vernum Sommerf. ex Fr.

* LAMcri is not represented in the database as it forms an unclear cluster with LAMspi, why this mock species is annotated as Lamproderma sp. from the alignment agains the reference MSA.

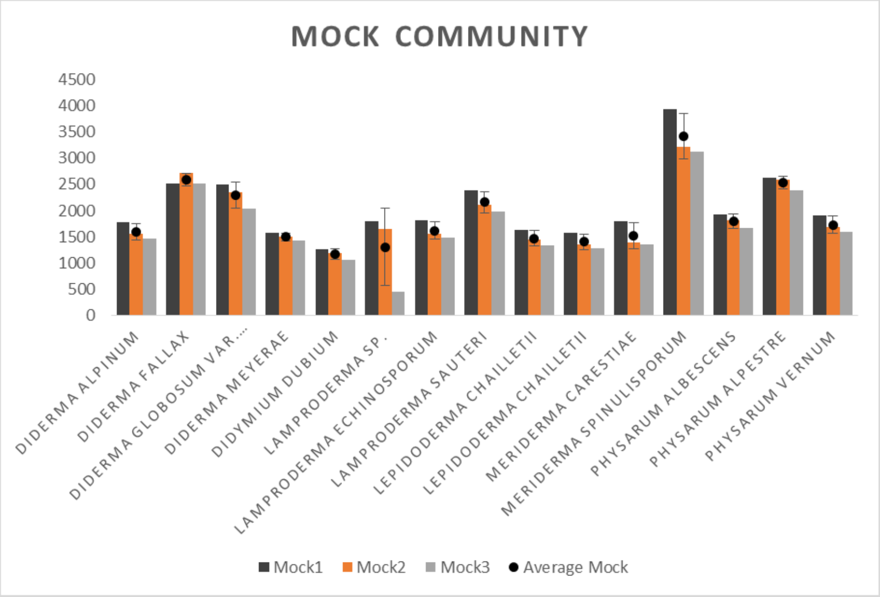

Supplementary S5 data for: A four year survey reveals a coherent pattern between distribution of fruit bodies and soil amoebae populations for nivicolous myxomycetes. M. Borg Dahl, O. Shchepin, C. Schunk, A. Menzel, Y. K. Novozhilov and M. Schnittler

Fruit sequences		
**sequence**	**Type**	**cluster nr**
GAP13_sc26415_LAMaen_SSU0398	Cluster rep	70
GAP16_sc29057_LAMaen_SSU1504	Cluster member	70
GAP16_sc29195_LAMaen_SSU1505	Cluster member	70
GAP16_sc28968_LAMaen_SSU1509	Cluster member	70
GAP16_sc28976_LAMaen_SSU1510	Cluster member	70
GAP13_sc26329_LAMaen_SSU0402	Cluster rep	1
GAP13_sc26334_LAMaen_SSU0401	Cluster member	1
GAP13_sc26322_LAMaen_SSU0400	Cluster member	1
GAP13_sc26366_LAMaen_SSU0399	Cluster member	1
GAP16_sc28973_LAMaen_SSU1508	Cluster member	1
GAP13_sc26394_LAMovo_SSU0447	Cluster rep	2
GAP13_sc26391_LAMovo_SSU0449	Cluster member	2
GAP13_sc26395_LAMovo_SSU0446	Cluster member	2
GAP13_sc26338_LAMovocuc_SSU0448	Cluster member	2
GAP13_sc26392_LAMovo_SSU0450	Cluster member	2
GAP16_sc29188_LAMovo_SSU1439	Cluster rep	3
GAP16_sc29000_LAMovo_SSU1444	Cluster rep	4
GAP15_sc28394_LAMzon_SSU0443	Cluster rep	5
GAP16_sc29078_LAMzon_SSU1447	Cluster member	5
GAP16_sc29079_LAMzon_SSU1448	Cluster member	5
GAP13_sc26443_LAMsau_SSU0438	Cluster rep	6
GAP15_sc28387_LAMsau_SSU0440	Cluster member	6
GAP15_sc28365_LAMsau_SSU0439	Cluster member	6
GAP13_sc26411_LAMsau_SSU0437	Cluster member	6
GAP16_sc28982_LAMsau_SSU1454	Cluster member	6
GAP16_sc29055_LAMsau_SSU1476	Cluster member	6
GAP16_sc29151_LAMsau_SSU1477	Cluster member	6
GAP16_sc29154_LAMsau_SSU1478	Cluster member	6
GAP16_sc29077_LAMsau_SSU1495	Cluster member	6
GAP16_sc29153_LAMsau_SSU1496	Cluster member	6
GAP16_sc28978_LAMsau_SSU1486	Cluster member	6
GAP15_sc28518_LAMsau_	Cluster member	6
GAP15_sc28500_LEPcha_SSU0027	Cluster rep	7
GAP15_sc28425_LEPcha_SSU0029	Cluster member	7
GAP15_sc28418_LEPcha_SSU0026	Cluster member	7
GAP15_sc28474_LEPcha_SSU0028	Cluster member	7
GAP15_sc28362_LEPcha_SSU0030	Cluster member	7
GAP16_sc29074_LEPcha_SSU1303	Cluster member	7
GAP16_sc29231_LEPcha_SSU1304	Cluster member	7
GAP16_sc29248_LEPcha_SSU1305	Cluster member	7
GAP16_sc29232_LEPcha_SSU1306	Cluster member	7
GAP16_sc29233_LEPcha_SSU1307	Cluster member	7
GAP15_sc28493_DIDgloeur_SSU0075	Cluster rep	8

Supplementary S5 data for: A four year survey reveals a coherent pattern between distribution of fruit bodies and soil amoebae populations for nivicolous myxomycetes. M. Borg Dahl, O. Shchepin, C. Schunk, A. Menzel, Y. K. Novozhilov and M. Schnittler

Fruit sequences		
**sequence**	**Type**	**cluster nr**
GAP13_sc26406_DIDgloeur_SSU0076	Cluster member	8
GAP15_sc28440_LAMpir_SSU0500	Cluster rep	9
GAP16_sc29185_PHYals_SSU1526	Cluster rep	10
GAP16_sc29043_MERcar_	Cluster rep	11
GAP15_sc28452_MERcar_	Cluster member	11
GAP13_sc26335_MERagg_SSU0338	Cluster rep	12
GAP15_sc28401_MERagg_	Cluster rep	13
GAP15_sc28476_PHYver_SSU0317	Cluster rep	14
GAP15_sc28417_PHYver_SSU0324	Cluster member	14
GAP13_sc26404_PHYver_SSU0326	Cluster member	14
GAP13_sc26337_PHYver_SSU0325	Cluster member	14
GAP13_sc26401_PHYver_SSU0327	Cluster member	14
GAP15_sc28475_PHYver_SSU0318	Cluster member	14
GAP15_sc28426_PHYver_SSU0320	Cluster member	14
GAP13_sc26374_PHYver_SSU0310	Cluster member	14
GAP16_sc29180_PHYver_SSU1617	Cluster member	14
GAP16_sc29183_PHYver_SSU1618	Cluster member	14
GAP16_sc29189_PHYver_SSU1619	Cluster member	14
GAP13_sc26288_LAMesp_SSU0002	Cluster rep	15
GAP15_sc28406_LAMesp_SSU0001	Cluster member	15
GAP13_sc26340_LAMesp_SSU0369	Cluster member	15
GAP15_sc28361_LAMesp_SSU0371	Cluster member	15
GAP15_sc28499_LAMesp_SSU0373	Cluster member	15
GAP13_sc26300_LAMesp_SSU0368	Cluster member	15
GAP15_sc28532_LAMesp_SSU0370	Cluster member	15
GAP15_sc28375_LAMesp_SSU0372	Cluster member	15
GAP16_sc29052_LAMesp_SSU1581	Cluster member	15
GAP16_sc29073_LAMesp_SSU1582	Cluster member	15
GAP16_sc29056_LAMesp_SSU1594	Cluster member	15
GAP16_sc29103_LAMesp_SSU1595	Cluster member	15
GAP16_sc29238_LAMesp_SSU1596	Cluster member	15
GAP16_sc29109_LAMesp_SSU1597	Cluster member	15
GAP16_sc29053_LAMesp_SSU1583	Cluster member	15
GAP16_sc29123_LAMesp_SSU1589	Cluster member	15
GAP16_sc29104_LAMesp_SSU1584	Cluster member	15
GAP16_sc29115_LAMesp_SSU1585	Cluster member	15
GAP16_sc29144_LAMesp_SSU1586	Cluster member	15
GAP16_sc29130_LAMesp_SSU1587	Cluster member	15
GAP16_sc29093_LAMesp_SSU1590	Cluster member	15
GAP16_sc29125_LAMesp_SSU1591	Cluster member	15
GAP16_sc29176_LAMesp_SSU1588	Cluster member	15
GAP16_sc29129_LAMesp_SSU1599	Cluster member	15
GAP16_sc29128_LAMesp_SSU1598	Cluster member	15

Supplementary S5 data for: A four year survey reveals a coherent pattern between distribution of fruit bodies and soil amoebae populations for nivicolous myxomycetes. M. Borg Dahl, O. Shchepin, C. Schunk, A. Menzel, Y. K. Novozhilov and M. Schnittler

Fruit sequences		
**sequence**	**Type**	**cluster nr**
GAP13_sc26407_LAMaen_SSU0405	Cluster rep	16
GAP16_sc28964_LAMaen_SSU1498	Cluster member	16
GAP16_sc28971_LAMaen_SSU1499	Cluster member	16
GAP13_sc26326_LAMovo_SSU0445	Cluster rep	17
GAP15_sc28489_LAMovo_SSU0460	Cluster rep	18
GAP15_sc28405_LAMovo_SSU0461	Cluster member	18
GAP16_sc28955_LAMovo_SSU1430	Cluster member	18
GAP16_sc28985_LAMovo_SSU1431	Cluster member	18
GAP16_sc28996_LAMovo_SSU1432	Cluster member	18
GAP16_sc28997_LAMovo_SSU1433	Cluster member	18
GAP16_sc28988_LAMovo_SSU1445	Cluster member	18
GAP16_sc28949_LAMovo_SSU1429	Cluster member	18
GAP13_sc26342_LAMovo_	Cluster member	18
GAP15_sc28487_LAMovo_	Cluster member	18
GAP16_sc29202_LAMovo_SSU1440	Cluster rep	19
GAP15_sc28414_LAMovo_	Cluster member	19
GAP15_sc28449_LAMovo_SSU0468	Cluster rep	20
GAP15_sc28416_LAMovo_SSU0470	Cluster member	20
GAP13_sc26297_LAMovo_SSU0472	Cluster member	20
GAP15_sc28422_LAMovo_SSU0474	Cluster member	20
GAP15_sc28379_LAMovo_SSU0476	Cluster member	20
GAP15_sc28368_LAMovo_SSU0478	Cluster member	20
GAP13_sc26393_LAMovo_SSU0480	Cluster member	20
GAP15_sc28497_LAMovo_SSU0469	Cluster member	20
GAP15_sc28383_LAMovo_SSU0471	Cluster member	20
GAP15_sc28502_LAMovo_SSU0473	Cluster member	20
GAP15_sc28373_LAMovo_SSU0475	Cluster member	20
GAP15_sc28544_LAMovo_SSU0477	Cluster member	20
GAP15_sc28366_LAMovo_SSU0479	Cluster member	20
GAP13_sc26416_LAMovo_SSU0483	Cluster member	20
GAP13_sc26365_LAMovo_SSU0486	Cluster member	20
GAP16_sc28951_LAMovo_SSU1414	Cluster member	20
GAP16_sc28961_LAMovo_SSU1415	Cluster member	20
GAP16_sc28975_LAMovo_SSU1416	Cluster member	20
GAP16_sc29003_LAMovo_SSU1417	Cluster member	20
GAP16_sc29169_LAMovo_SSU1418	Cluster member	20
GAP16_sc28970_LAMovo_SSU1420	Cluster member	20
GAP16_sc29092_LAMovo_SSU1419	Cluster member	20
GAP16_sc29001_LAMovo_SSU1421	Cluster member	20
GAP16_sc29020_LAMovo_SSU1422	Cluster member	20
GAP16_sc29067_LAMovo_SSU1423	Cluster member	20
GAP16_sc29064_LAMovo_SSU1424	Cluster member	20
GAP16_sc29095_LAMovo_SSU1425	Cluster member	20

Supplementary S5 data for: A four year survey reveals a coherent pattern between distribution of fruit bodies and soil amoebae populations for nivicolous myxomycetes. M. Borg Dahl, O. Shchepin, C. Schunk, A. Menzel, Y. K. Novozhilov and M. Schnittler

Fruit sequences		
**sequence**	**Type**	**cluster nr**
GAP15_sc28501_LAMovo_SSU0481	Cluster member	20
GAP16_sc29167_LAMovo_SSU1427	Cluster member	20
GAP16_sc28952_LAMovo_SSU1426	Cluster member	20
GAP16_sc29040_LAMovo_SSU1428	Cluster member	20
GAP15_sc28386_LAMovo_	Cluster member	20
GAP15_sc28409_LAMovo_	Cluster member	20
GAP15_sc28359_LAMovo_	Cluster member	20
GAP15_sc28451_LAMovo_	Cluster member	20
GAP15_sc28358_LAMovo_SSU0488	Cluster rep	21
GAP13_sc26439_LAMovo_SSU0491	Cluster member	21
GAP13_sc26383_LAMovo_SSU0493	Cluster member	21
GAP15_sc28363_LAMovo_SSU0489	Cluster member	21
GAP13_sc26386_LAMovo_SSU0492	Cluster member	21
GAP16_sc29193_LAMovo_SSU1374	Cluster member	21
GAP16_sc28969_LAMovo_SSU1408	Cluster member	21
GAP16_sc28947_LAMovo_SSU1407	Cluster member	21
GAP16_sc28953_LAMovo_SSU1410	Cluster member	21
GAP16_sc29045_LAMovo_SSU1412	Cluster member	21
GAP16_sc29112_LAMovo_SSU1413	Cluster member	21
GAP16_sc29032_LAMovo_SSU1411	Cluster member	21
GAP15_sc28511_LAMovo_	Cluster member	21
GAP15_sc28521_LAMsau_SSU0420	Cluster rep	22
GAP15_sc28351_LAMsau_SSU0424	Cluster member	22
GAP15_sc28374_LAMsau_SSU0426	Cluster member	22
GAP15_sc28388_LAMsau_SSU0428	Cluster member	22
GAP15_sc28453_LAMsau_SSU0430	Cluster member	22
GAP15_sc28382_LAMsau_SSU0432	Cluster member	22
GAP15_sc28390_LAMsau_SSU0434	Cluster member	22
GAP15_sc28546_LAMsau_SSU0436	Cluster member	22
GAP13_sc26324_LAMsau_SSU0422	Cluster member	22
GAP15_sc28353_LAMsau_SSU0425	Cluster member	22
GAP15_sc28384_LAMsau_SSU0427	Cluster member	22
GAP15_sc28430_LAMsau_SSU0429	Cluster member	22
GAP15_sc28380_LAMsau_SSU0431	Cluster member	22
GAP15_sc28385_LAMsau_SSU0433	Cluster member	22
GAP15_sc28506_LAMsau_SSU0435	Cluster member	22
GAP15_sc28508_LAMsau_SSU0418	Cluster member	22
GAP16_sc28956_LAMsau_SSU1452	Cluster member	22
GAP16_sc29009_LAMsau_SSU1453	Cluster member	22
GAP16_sc28960_LAMsau_SSU1455	Cluster member	22
GAP16_sc28980_LAMsau_SSU1456	Cluster member	22
GAP16_sc28986_LAMsau_SSU1457	Cluster member	22
GAP16_sc28991_LAMsau_SSU1458	Cluster member	22

Supplementary S5 data for: A four year survey reveals a coherent pattern between distribution of fruit bodies and soil amoebae populations for nivicolous myxomycetes. M. Borg Dahl, O. Shchepin, C. Schunk, A. Menzel, Y. K. Novozhilov and M. Schnittler

Fruit sequences		
**sequence**	**Type**	**cluster nr**
GAP16_sc28967_LAMsau_SSU1459	Cluster member	22
GAP16_sc28959_LAMsau_SSU1460	Cluster member	22
GAP16_sc28963_LAMsau_SSU1461	Cluster member	22
GAP16_sc28972_LAMsau_SSU1462	Cluster member	22
GAP16_sc28983_LAMsau_SSU1463	Cluster member	22
GAP16_sc29017_LAMsau_SSU1464	Cluster member	22
GAP16_sc29194_LAMsau_SSU1465	Cluster member	22
GAP16_sc28974_LAMsau_SSU1466	Cluster member	22
GAP16_sc28984_LAMsau_SSU1467	Cluster member	22
GAP16_sc29004_LAMsau_SSU1468	Cluster member	22
GAP16_sc29083_LAMsau_SSU1485	Cluster member	22
GAP16_sc29006_LAMsau_SSU1469	Cluster member	22
GAP16_sc29015_LAMsau_SSU1470	Cluster member	22
GAP16_sc29150_LAMsau_SSU1471	Cluster member	22
GAP16_sc29016_LAMsau_SSU1472	Cluster member	22
GAP16_sc29152_LAMsau_SSU1473	Cluster member	22
GAP16_sc29042_LAMsau_SSU1474	Cluster member	22
GAP16_sc29008_LAMsau_SSU1475	Cluster member	22
GAP16_sc29039_LAMsau_SSU1491	Cluster member	22
GAP16_sc29148_LAMsau_SSU1492	Cluster member	22
GAP16_sc29111_LAMsau_SSU1493	Cluster member	22
GAP16_sc29075_LAMsau_SSU1494	Cluster member	22
GAP16_sc28958_LAMsau_SSU1487	Cluster member	22
GAP15_sc28372_LAMsau_	Cluster member	22
GAP15_sc28377_LAMsau_	Cluster member	22
GAP15_sc28376_LAMarc_SSU0385	Cluster rep	23
GAP13_sc26376_LAMarc_SSU0384	Cluster member	23
GAP16_sc29149_LAMarc_SSU1521	Cluster member	23
GAP13_sc26390_DIDfal_SSU0015	Cluster rep	24
GAP15_sc28458_DIDfal_SSU0017	Cluster member	24
GAP13_LE296545_DIDfal_SSU0013	Cluster member	24
GAP13_sc26389_DIDfal_SSU0016	Cluster member	24
GAP15_sc28478_DIDfal_SSU0018	Cluster member	24
GAP16_sc29134_DIDfal_SSU1308	Cluster member	24
GAP16_sc29243_DIDfal_SSU1310	Cluster member	24
GAP16_sc29178_DIDfal_SSU1317	Cluster member	24
GAP16_sc29239_DIDfal_SSU1312	Cluster member	24
GAP16_sc29262_DIDfal_SSU1313	Cluster member	24
GAP16_sc29242_DIDfal_SSU1311	Cluster member	24
GAP17_sc29707_DDYdub_	Cluster rep	25
GAP17_sc29710_DDYdub_	Cluster member	25
GAP15_sc28467_DIDgloeur_SSU0053	Cluster rep	26
GAP13_sc26414_DIDgloeur_SSU0080	Cluster member	26

Supplementary S5 data for: A four year survey reveals a coherent pattern between distribution of fruit bodies and soil amoebae populations for nivicolous myxomycetes. M. Borg Dahl, O. Shchepin, C. Schunk, A. Menzel, Y. K. Novozhilov and M. Schnittler

Fruit sequences		
**sequence**	**Type**	**cluster nr**
GAP15_sc28540_DIDgloeur_SSU0082	Cluster member	26
GAP15_sc28461_DIDgloeur_SSU0084	Cluster member	26
GAP15_sc28541_DIDgloeur_SSU0086	Cluster member	26
GAP15_sc28472_DIDgloeur_SSU0088	Cluster member	26
GAP15_sc28519_DIDgloeur_SSU0091	Cluster member	26
GAP13_sc26417_DIDgloeur_SSU0081	Cluster member	26
GAP15_sc28473_DIDgloeur_SSU0083	Cluster member	26
GAP15_sc28488_DIDgloeur_SSU0085	Cluster member	26
GAP15_sc28434_DIDgloeur_SSU0087	Cluster member	26
GAP13_sc26442_DIDgloeur_SSU0089	Cluster member	26
GAP17_sc29700_DIDgloeur_	Cluster member	26
GAP16_sc29028_DIDgloeur_SSU1389	Cluster member	26
GAP16_sc29082_DIDgloeur_SSU1392	Cluster member	26
GAP16_sc29126_DIDgloeur_SSU1393	Cluster member	26
GAP16_sc29094_DIDgloeur_SSU1394	Cluster member	26
GAP16_sc29133_DIDgloeur_SSU1395	Cluster member	26
GAP16_sc29137_DIDgloeur_SSU1396	Cluster member	26
GAP16_sc29132_DIDgloeur_SSU1390	Cluster member	26
GAP16_sc29170_DIDgloeur_SSU1400	Cluster member	26
GAP16_sc29187_DIDgloeur_SSU1401	Cluster member	26
GAP16_sc29098_DIDgloeur_SSU1399	Cluster member	26
GAP16_sc29049_DIDgloeur_SSU1404	Cluster member	26
GAP13_sc26358_DIDgloeur_SSU0078	Cluster rep	27
GAP16_sc29087_DIDgloeur_SSU1405	Cluster member	27
GAP15_sc28491_DIDniv_SSU0142	Cluster rep	28
GAP15_sc28469_DIDniv_SSU0144	Cluster member	28
GAP15_sc28464_DIDniv_SSU0146	Cluster member	28
GAP15_sc28463_DIDniv_SSU0139	Cluster member	28
GAP15_sc28477_DIDniv_SSU0143	Cluster member	28
GAP15_sc28437_DIDniv_SSU0145	Cluster member	28
GAP15_sc28395_DIDniv_SSU0147	Cluster member	28
GAP16_sc29048_DIDniv_SSU1384	Cluster member	28
GAP15_sc28420_DIDniv_	Cluster member	28
GAP15_sc28396_DIDmey_SSU0152	Cluster rep	29
GAP13_sc26367_DIDmey_SSU0154	Cluster member	29
GAP13_sc26298_DIDmey_SSU0156	Cluster member	29
GAP13_sc26379_DIDmey_SSU0158	Cluster member	29
GAP15_sc28431_DIDmey_SSU0160	Cluster member	29
GAP15_sc28466_DIDmey_SSU0162	Cluster member	29
GAP13_sc26312_DIDmey_SSU0164	Cluster member	29
GAP13_sc26316_DIDmey_SSU0166	Cluster member	29
GAP13_sc26323_DIDmey_SSU0168	Cluster member	29
GAP13_sc26351_DIDmey_SSU0170	Cluster member	29

Supplementary S5 data for: A four year survey reveals a coherent pattern between distribution of fruit bodies and soil amoebae populations for nivicolous myxomycetes. M. Borg Dahl, O. Shchepin, C. Schunk, A. Menzel, Y. K. Novozhilov and M. Schnittler

Fruit sequences		
**sequence**	**Type**	**cluster nr**
GAP13_sc26359_DIDmey_SSU0172	Cluster member	29
GAP13_sc26362_DIDmey_SSU0174	Cluster member	29
GAP15_sc28551_DIDmey_SSU0176	Cluster member	29
GAP13_sc26378_DIDmey_SSU0178	Cluster member	29
GAP13_sc26305_DIDmey_SSU0180	Cluster member	29
GAP13_sc26352_DIDmey_SSU0182	Cluster member	29
GAP15_sc28539_DIDmey_SSU0184	Cluster member	29
GAP13_sc26373_DIDmey_SSU0188	Cluster member	29
GAP13_sc26370_DIDmey_SSU0190	Cluster member	29
GAP15_sc28533_DIDmey_SSU0153	Cluster member	29
GAP15_sc28538_DIDmey_SSU0155	Cluster member	29
GAP13_sc26424_DIDmey_SSU0157	Cluster member	29
GAP15_sc28423_DIDmey_SSU0159	Cluster member	29
GAP15_sc28462_DIDmey_SSU0161	Cluster member	29
GAP15_sc28483_DIDmey_SSU0163	Cluster member	29
GAP13_sc26308_DIDmey_SSU0165	Cluster member	29
GAP13_sc26318_DIDmey_SSU0167	Cluster member	29
GAP13_sc26347_DIDmey_SSU0169	Cluster member	29
GAP13_sc26354_DIDmey_SSU0171	Cluster member	29
GAP13_sc26361_DIDmey_SSU0173	Cluster member	29
GAP13_sc26363_DIDmey_SSU0175	Cluster member	29
GAP13_sc26301_DIDmey_SSU0177	Cluster member	29
GAP15_sc28400_DIDmey_SSU0179	Cluster member	29
GAP13_sc26292_DIDmey_SSU0181	Cluster member	29
GAP13_sc26294_DIDmey_SSU0183	Cluster member	29
GAP13_sc26377_DIDmey_SSU0189	Cluster member	29
GAP15_sc28534_DIDmey_SSU0218	Cluster member	29
GAP16_sc29054_DIDmey_SSU1339	Cluster member	29
GAP16_sc29061_DIDmey_SSU1340	Cluster member	29
GAP16_sc29218_DIDmey_SSU1341	Cluster member	29
GAP16_sc29221_DIDmey_SSU1342	Cluster member	29
GAP16_sc29222_DIDmey_SSU1343	Cluster member	29
GAP16_sc29223_DIDmey_SSU1344	Cluster member	29
GAP16_sc29212_DIDmey_SSU1356	Cluster member	29
GAP16_sc29230_DIDmey_SSU1357	Cluster member	29
GAP16_sc29253_DIDmey_SSU1358	Cluster member	29
GAP16_sc29021_DIDmey_SSU1349	Cluster member	29
GAP16_sc29022_DIDmey_SSU1350	Cluster member	29
GAP16_sc29117_DIDmey_SSU1351	Cluster member	29
GAP16_sc29122_DIDmey_SSU1352	Cluster member	29
GAP16_sc29140_DIDmey_SSU1353	Cluster member	29
GAP16_sc29217_DIDmey_SSU1354	Cluster member	29
GAP16_sc29142_DIDmey_SSU1355	Cluster member	29

Supplementary S5 data for: A four year survey reveals a coherent pattern between distribution of fruit bodies and soil amoebae populations for nivicolous myxomycetes. M. Borg Dahl, O. Shchepin, C. Schunk, A. Menzel, Y. K. Novozhilov and M. Schnittler

Fruit sequences		
**sequence**	**Type**	**cluster nr**
GAP16_sc29120_DIDmey_SSU1364	Cluster member	29
GAP16_sc29219_DIDmey_SSU1365	Cluster member	29
GAP16_sc29260_DIDmey_SSU1348	Cluster member	29
GAP16_sc29010_DIDmey_SSU1367	Cluster member	29
GAP16_sc29156_DIDmey_SSU1368	Cluster member	29
GAP16_sc29259_DIDmey_SSU1369	Cluster member	29
GAP16_sc29249_DIDmey_SSU1345	Cluster member	29
GAP16_sc29251_DIDmey_SSU1360	Cluster member	29
GAP16_sc29213_DIDmey_SSU1346	Cluster member	29
GAP16_sc29258_DIDmey_SSU1361	Cluster member	29
GAP16_sc29263_DIDmey_SSU1362	Cluster member	29
GAP16_sc29229_DIDmey_SSU1347	Cluster member	29
GAP16_sc29019_DIDmey_SSU1371	Cluster member	29
GAP16_sc29012_DIDmey_SSU1373	Cluster member	29
GAP16_sc29139_DIDmey_SSU1374	Cluster member	29
GAP16_sc29146_DIDmey_SSU1375	Cluster member	29
GAP16_sc29066_DIDmey_SSU1372	Cluster member	29
GAP16_sc29036_DIDmey_SSU1376	Cluster member	29
GAP16_sc29058_DIDmey_SSU1377	Cluster member	29
GAP16_sc29014_DIDmey_SSU1378	Cluster member	29
GAP16_sc29227_DIDmey_SSU1379	Cluster member	29
GAP17_sc29708_DIDmey_	Cluster member	29
GAP17_sc29709_DIDmey_	Cluster member	29
GAP15_sc28433_DIDmey_SSU0187	Cluster member	29
GAP13_sc26293_DIDmey_SSU0499	Cluster member	29
GAP16_sc29226_DIDmey_SSU1380	Cluster member	29
GAP15_sc28421_PHYalb_SSU0328	Cluster rep	30
GAP13_sc26341_MERspi_SSU0347	Cluster rep	31
GAP13_sc26319_MERspi_SSU0346	Cluster member	31
GAP13_sc26344_MERcar_SSU0353	Cluster rep	32
GAP15_sc28448_MERcar_SSU0354	Cluster member	32
GAP16_sc28979_MERcar_SSU1606	Cluster rep	33
GAP16_sc28981_MERcar_SSU1607	Cluster member	33
GAP15_sc28450_MERcar_	Cluster member	33
GAP15_sc28404_MERagg_SSU0341	Cluster rep	34
GAP13_sc26382_MERagg_SSU0345	Cluster member	34
GAP13_sc26330_MERagg_SSU0342	Cluster member	34
GAP16_sc29025_MERagg_SSU1612	Cluster member	34
GAP15_sc28397_MERagg_	Cluster member	34
GAP13_sc26384_MERagg_	Cluster member	34
GAP13_sc26310_PHYver_SSU0311	Cluster rep	35
GAP13_sc26280_PHYver_SSU0313	Cluster member	35
GAP15_sc28514_PHYver_SSU0315	Cluster member	35

Supplementary S5 data for: A four year survey reveals a coherent pattern between distribution of fruit bodies and soil amoebae populations for nivicolous myxomycetes. M. Borg Dahl, O. Shchepin, C. Schunk, A. Menzel, Y. K. Novozhilov and M. Schnittler

Fruit sequences		
**sequence**	**Type**	**cluster nr**
GAP15_sc28528_PHYver_SSU0314	Cluster member	35
GAP15_sc28512_PHYver_SSU0312	Cluster member	35
GAP16_sc29182_PHYver_SSU1403	Cluster member	35
GAP16_sc29091_PHYver_SSU1614	Cluster member	35
GAP16_sc29096_PHYver_SSU1615	Cluster member	35
GAP16_sc29143_PHYver_SSU1620	Cluster member	35
GAP15_sc28479_PHYver_SSU0316	Cluster rep	36
GAP15_sc28516_LAMalb_SSU0377	Cluster rep	37
GAP13_sc26448_LAMalb_SSU0379	Cluster member	37
GAP13_sc26333_LAMalb_SSU0381	Cluster member	37
GAP15_sc28447_LAMalb_SSU0382	Cluster member	37
GAP13_sc26433_LAMalb_SSU0375	Cluster member	37
GAP13_sc26450_LAMalb_SSU0378	Cluster member	37
GAP16_sc28990_LAMalb_SSU1514	Cluster member	37
GAP16_sc29155_LAMalb_SSU1515	Cluster member	37
GAP16_sc29175_LAMalb_SSU1516	Cluster member	37
GAP16_sc29210_LAMalb_SSU1517	Cluster member	37
GAP16_sc29050_LAMalb_SSU1518	Cluster member	37
GAP16_sc29118_LAMalb_SSU1519	Cluster member	37
GAP16_sc29051_LAMalb_SSU1520	Cluster member	37
GAP13_sc26381_LAMalb_	Cluster member	37
GAP15_sc28432_LAMrrs_SSU0387	Cluster rep	38
GAP16_sc29065_LAMrrs_SSU1326	Cluster member	38
GAP13_sc26304_LAMaen_SSU0396	Cluster rep	39
GAP15_sc28510_LAMaen_	Cluster member	39
GAP13_sc26371_LAMovo_SSU0453	Cluster rep	40
GAP13_sc26357_LAMovo_SSU0464	Cluster member	40
GAP15_sc28370_LAMovo_SSU0466	Cluster member	40
GAP15_sc28446_LAMovo_SSU0465	Cluster member	40
GAP15_sc28355_LAMovo_SSU0467	Cluster member	40
GAP13_sc26412_LAMovo_SSU0463	Cluster member	40
GAP15_sc28520_LAMovo_SSU0455	Cluster member	40
GAP16_sc28987_LAMovo_SSU1434	Cluster member	40
GAP16_sc28992_LAMovo_SSU1435	Cluster member	40
GAP16_sc28999_LAMovo_SSU1436	Cluster member	40
GAP16_sc28998_LAMovo_SSU1437	Cluster member	40
GAP16_sc29106_LAMovo_SSU1438	Cluster member	40
GAP16_sc29147_LAMovo_SSU1442	Cluster member	40
GAP16_sc29113_LAMovo_SSU1446	Cluster member	40
GAP15_sc28456_LAMsau_SSU0407	Cluster rep	41
GAP15_sc28480_LAMsau_SSU0409	Cluster member	41
GAP15_sc28356_LAMsau_SSU0411	Cluster member	41
GAP15_sc28360_LAMsau_SSU0413	Cluster member	41

Supplementary S5 data for: A four year survey reveals a coherent pattern between distribution of fruit bodies and soil amoebae populations for nivicolous myxomycetes. M. Borg Dahl, O. Shchepin, C. Schunk, A. Menzel, Y. K. Novozhilov and M. Schnittler

Fruit sequences		
**sequence**	**Type**	**cluster nr**
GAP15_sc28484_LAMsau_SSU0408	Cluster member	41
GAP15_sc28415_LAMsau_SSU0410	Cluster member	41
GAP13_sc26350_LAMsau_SSU0412	Cluster member	41
GAP15_sc28495_LAMsau_SSU0414	Cluster member	41
GAP13_sc26409_LAMcrs_	Cluster rep	42
GAP16_sc29108_DIDfal_SSU1309	Cluster rep	43
GAP16_sc29162_DIDfal_SSU1314	Cluster member	43
GAP16_sc29165_DIDfal_SSU1315	Cluster member	43
GAP16_sc29174_DIDfal_SSU1316	Cluster member	43
GAP15_sc28442_LEPcha_SSU0025	Cluster rep	44
GAP13_sc26426_DDYdub_SSU0294	Cluster rep	45
GAP13_sc26331_DDYdub_SSU0295	Cluster member	45
GAP17_sc29711_DDYdub_	Cluster member	45
GAP16_sc29252_DDYdub_SSU1406	Cluster member	45
GAP15_sc28470_DIDniv_SSU0096	Cluster rep	46
GAP16_sc29046_DIDmey_SSU1363	Cluster rep	47
GAP16_sc29257_DIDmey_SSU1366	Cluster rep	48
GAP13_sc26364_DIDalp_SSU0252	Cluster rep	49
GAP13_sc26425_DIDalp_SSU0259	Cluster member	49
GAP13_sc26289_DIDalp_SSU0268	Cluster member	49
GAP15_sc28545_DIDalp_SSU0272	Cluster member	49
GAP15_sc28468_DIDalp_SSU0274	Cluster member	49
GAP15_sc28413_DIDalp_SSU0276	Cluster member	49
GAP15_sc28403_DIDalp_SSU0278	Cluster member	49
GAP15_sc28444_DIDalp_SSU0280	Cluster member	49
GAP13_sc26444_DIDalp_SSU0282	Cluster member	49
GAP15_sc28531_DIDalp_SSU0284	Cluster member	49
GAP15_sc28459_DIDalp_SSU0255	Cluster member	49
GAP13_sc26281_DIDalp_SSU0260	Cluster member	49
GAP13_sc26317_DIDalp_SSU0267	Cluster member	49
GAP13_sc26388_DIDalp_SSU0269	Cluster member	49
GAP13_sc26427_DIDalp_SSU0271	Cluster member	49
GAP13_sc26348_DIDalp_SSU0273	Cluster member	49
GAP15_sc28507_DIDalp_SSU0275	Cluster member	49
GAP15_sc28454_DIDalp_SSU0277	Cluster member	49
GAP15_sc28498_DIDalp_SSU0279	Cluster member	49
GAP13_sc26387_DIDalp_SSU0281	Cluster member	49
GAP13_sc26353_DIDalp_SSU0283	Cluster member	49
GAP15_sc28543_DIDalp_SSU0285	Cluster member	49
GAP16_sc29002_DIDalp_SSU1318	Cluster member	49
GAP16_sc29171_DIDalp_SSU1319	Cluster member	49
GAP16_sc29159_DIDalp_SSU1320	Cluster member	49
GAP16_sc29011_DIDalp_SSU1321	Cluster member	49

Supplementary S5 data for: A four year survey reveals a coherent pattern between distribution of fruit bodies and soil amoebae populations for nivicolous myxomycetes. M. Borg Dahl, O. Shchepin, C. Schunk, A. Menzel, Y. K. Novozhilov and M. Schnittler

Fruit sequences		
**sequence**	**Type**	**cluster nr**
GAP16_sc29068_DIDalp_SSU1322	Cluster member	49
GAP16_sc29110_DIDalp_SSU1323	Cluster member	49
GAP16_sc29136_DIDalp_SSU1324	Cluster member	49
GAP16_sc29127_DIDalp_SSU1325	Cluster member	49
GAP16_sc29085_DIDalp_SSU1326	Cluster member	49
GAP16_sc29070_DIDalp_SSU1328	Cluster member	49
GAP16_sc29018_DIDalp_SSU1329	Cluster member	49
GAP16_sc29101_DIDalp_SSU1330	Cluster member	49
GAP16_sc29141_DIDalp_SSU1331	Cluster member	49
GAP16_sc29119_DIDalp_SSU1332	Cluster member	49
GAP16_sc29234_DIDalp_SSU1333	Cluster member	49
GAP16_sc29027_DIDalp_SSU1334	Cluster member	49
GAP16_sc29114_DIDalp_SSU1335	Cluster member	49
GAP16_sc29186_DIDalp_SSU1327	Cluster member	49
GAP16_sc29099_DIDalp_SSU1336	Cluster member	49
GAP13_sc26282_DIDalp_	Cluster member	49
GAP15_sc28435_PHYals_SSU0364	Cluster rep	50
GAP16_sc29166_PHYals_SSU1525	Cluster member	50
GAP16_sc29172_PHYalb_SSU1527	Cluster rep	51
GAP16_sc29097_MERagg_SSU1613	Cluster rep	52
GAP15_sc28354_MERcar_SSU0351	Cluster rep	53
GAP16_sc28994_MERcar_SSU1609	Cluster member	53
GAP16_sc29158_MERcar_SSU1604	Cluster member	53
GAP16_sc29135_MERcar_SSU1608	Cluster member	53
GAP16_sc29163_MERech_SSU1610	Cluster rep	54
GAP15_sc28398_MERagg_	Cluster rep	55
GAP16_sc29035_LAMaen_SSU1500	Cluster rep	56
GAP16_sc29072_LAMaen_SSU1501	Cluster member	56
GAP16_sc29076_LAMaen_SSU1502	Cluster member	56
GAP16_sc29145_LAMaen_SSU1503	Cluster member	56
GAP16_sc29007_LAMaen_SSU1507	Cluster member	56
GAP16_sc29090_LAMaen_SSU1506	Cluster member	56
GAP15_sc28524_LAMaen_SSU0392	Cluster member	56
GAP15_sc28436_LAMaen_SSU0394	Cluster member	56
GAP13_sc26315_LAMaen_SSU0390	Cluster member	56
GAP15_sc28445_LAMaen_SSU0395	Cluster member	56
GAP15_sc28490_LAMaen_	Cluster member	56
GAP16_sc28989_LAMovo_SSU1443	Cluster rep	57
GAP15_sc28408_LAMsau_SSU0417	Cluster rep	58
GAP15_sc28402_LAMsau_SSU0416	Cluster member	58
GAP16_sc28948_LAMsau_SSU1449	Cluster member	58
GAP16_sc29168_LAMsau_SSU1450	Cluster member	58
GAP16_sc28950_LAMsau_SSU1451	Cluster member	58

Supplementary S5 data for: A four year survey reveals a coherent pattern between distribution of fruit bodies and soil amoebae populations for nivicolous myxomycetes. M. Borg Dahl, O. Shchepin, C. Schunk, A. Menzel, Y. K. Novozhilov and M. Schnittler

Fruit sequences		
**sequence**	**Type**	**cluster nr**
GAP16_sc29157_LAMsau_SSU1479	Cluster member	58
GAP16_sc29164_LAMsau_SSU1480	Cluster member	58
GAP16_sc28993_LAMsau_SSU1481	Cluster member	58
GAP16_sc28995_LAMsau_SSU1482	Cluster member	58
GAP16_sc29005_LAMsau_SSU1483	Cluster member	58
GAP16_sc29024_LAMsau_SSU1484	Cluster member	58
GAP16_sc29023_LAMsau_SSU1488	Cluster member	58
GAP16_sc29121_LAMsau_SSU1489	Cluster member	58
GAP16_sc29131_LAMsau_SSU1490	Cluster member	58
GAP16_sc29160_LAMsau_SSU1497	Cluster member	58
GAP13_sc26436_LAMsau_SSU0406	Cluster rep	59
GAP16_sc29089_LAMpul_SSU1513	Cluster rep	60
GAP15_sc28369_LAMarc_SSU0386	Cluster rep	61
GAP16_sc29173_LAMarc_SSU1522	Cluster member	61
GAP16_sc29059_LEPcha_SSU1302	Cluster rep	62
GAP13_sc26302_LEPcha_SSU0038	Cluster rep	63
GAP15_sc28536_LEPcha_SSU0039	Cluster member	63
GAP16_sc29013_LEPcha_SSU1286	Cluster member	63
GAP16_sc29033_LEPcha_SSU1287	Cluster member	63
GAP16_sc29037_LEPcha_SSU1288	Cluster member	63
GAP16_sc29038_LEPcha_SSU1289	Cluster member	63
GAP16_sc29224_LEPcha_SSU1291	Cluster member	63
GAP16_sc29236_LEPcha_SSU1292	Cluster member	63
GAP16_sc29245_LEPcha_SSU1293	Cluster member	63
GAP16_sc29246_LEPcha_SSU1294	Cluster member	63
GAP16_sc29225_LEPcha_SSU1295	Cluster member	63
GAP16_sc29240_LEPcha_SSU1296	Cluster member	63
GAP16_sc29247_LEPcha_SSU1297	Cluster member	63
GAP16_sc29250_LEPcha_SSU1299	Cluster member	63
GAP16_sc29181_LEPcha_SSU1290	Cluster member	63
GAP16_sc29228_LEPcha_SSU1300	Cluster member	63
GAP16_sc29034_LEPcha_	Cluster member	63
GAP17_sc29701_DDYdub_	Cluster rep	64
GAP17_sc29702_DDYdub_	Cluster member	64
GAP17_sc29703_DDYdub_	Cluster member	64
GAP17_sc29704_DDYdub_	Cluster member	64
GAP17_sc29705_DDYdub_	Cluster member	64
GAP17_sc29706_DDYdub_	Cluster member	64
GAP13_sc26327_DDYdif_SSU0302	Cluster rep	65
GAP15_sc28352_DDYdif_SSU0304	Cluster member	65
GAP13_sc26332_DDYdif_SSU0303	Cluster member	65
GAP16_sc28962_DDYdif_SSU1523	Cluster member	65
GAP16_sc29044_DDYdif_SSU1524	Cluster member	65

Supplementary S5 data for: A four year survey reveals a coherent pattern between distribution of fruit bodies and soil amoebae populations for nivicolous myxomycetes. M. Borg Dahl, O. Shchepin, C. Schunk, A. Menzel, Y. K. Novozhilov and M. Schnittler

Fruit sequences		
**sequence**	**Type**	**cluster nr**
GAP16_sc29029_DDYdif_	Cluster member	65
GAP13_sc26445_DIDgloeur_SSU0055	Cluster rep	66
GAP15_sc28509_DIDgloeur_SSU0054	Cluster member	66
GAP15_sc28410_DIDgloeur_SSU0074	Cluster member	66
GAP15_sc28471_DIDgloeur_SSU0092	Cluster member	66
GAP16_sc29100_DIDgloeur_SSU1398	Cluster member	66
GAP16_sc29030_DIDgloeur_SSU1391	Cluster member	66
GAP16_sc29184_DIDgloeur_SSU1403	Cluster member	66
GAP16_sc29161_DIDgloeur_SSU1397	Cluster rep	67
GAP16_sc29107_DIDgloeur_SSU1402	Cluster member	67
GAP15_sc28460_DIDmic_SSU0236	Cluster rep	68
GAP15_sc28367_DIDmic_SSU0242	Cluster member	68
GAP16_sc29102_DIDmic_	Cluster member	68
GAP15_sc28381_MERcar_	Cluster rep	69
GAP16_sc29192_MERcar_SSU1601	Cluster member	69
GAP16_sc29198_MERcar_SSU1602	Cluster member	69
GAP16_sc29209_MERcar_SSU1603	Cluster member	69

Supplementarty S5 data for: A four year survey reveals a coherent pattern between distribution of fruit bodies and soil amoebae populations for nivicolous myxomycetes. M. Borg Dahl, O. Shchepin, C. Schunk, A. Menzel, Y. K. Novozhilov and M. Schnittler

Fruit match to soil OTU												
**Fruit Ribo (unique GAP)**	**size**	**Match (soil seq)**	**Db size**	**% ID**	**lenght**	**mismatche**	**gap opens**	**q. start**	**q. end**	**s. start**	**s. end**	**E-value**
GAP15_sc28404_MERagg_SSU0341	6	seq74	2296	87.273	330	29	12	1	329	20	337	1E-102
GAP13_sc26335_MERagg_SSU0338	1	seq74	2296	87.273	330	29	12	1	329	20	337	1E-102
GAP15_sc28401_MERagg_	1	seq74	2296	87.273	330	29	11	1	329	20	337	1E-102
GAP16_sc29097_MERagg_SSU1613	1	seq6587	10	87.463	335	24	14	1	334	17	334	2.2E-104
GAP15_sc28398_MERagg_	1	seq74	2296	87.576	330	28	11	1	329	20	337	2.2E-104
GAP16_sc29089_LAMpul_SSU1513	1	seq768	134	88.991	327	24	12	1	321	17	337	1.2E-111
GAP13_sc26326_LAMovo_SSU0445	1	seq768	134	93.189	323	20	2	1	323	17	337	1.6E-135
GAP13_sc26394_LAMovo_SSU0447	5	seq768	134	93.498	323	19	2	1	323	17	337	3.4E-137
GAP15_sc28394_LAMzon_SSU0443	3	seq768	134	94.081	321	17	2	1	319	17	337	2E-139
GAP15_sc28516_LAMalb_SSU0377	14	seq639	196	96.393	305	11	0	1	305	8	312	1.9E-144
GAP16_sc29172_PHYalb_SSU1527	1	seq3378	20	96.439	337	11	1	1	336	11	347	5.6E-160
GAP15_sc28449_LAMovo_SSU0468	35	seq155	1711	97.205	322	8	1	1	322	17	337	1.2E-156
GAP16_sc29163_MERech_SSU1610	1	seq6517	10	98.113	318	6	0	1	318	11	328	5.3E-160
GAP16_sc28989_LAMovo_SSU1443	1	seq768	134	98.131	321	6	0	1	321	17	337	1.2E-161
GAP13_sc26436_LAMsau_SSU0406	1	seq1272	64	98.418	316	5	0	1	316	11	326	1.5E-160
GAP13_sc26304_LAMaen_SSU0396	2	seq776	123	98.746	319	2	2	1	317	20	338	2.5E-163
GAP16_sc29108_DIDfal_SSU1309	4	seq6261	11	98.75	320	4	0	1	320	17	336	1.9E-164
GAP15_sc28493_DIDgloeur_SSU0075	2	seq151	1682	99.063	320	3	0	1	320	11	330	4.1E-166
GAP16_sc29202_LAMovo_SSU1440	2	seq768	134	99.065	321	3	0	1	321	17	337	1.2E-166
GAP13_sc26445_DIDgloeur_SSU0055	7	seq41	3217	99.375	320	2	0	1	320	20	339	8.8E-168
GAP16_sc29188_LAMovo_SSU1439	1	seq768	134	99.377	321	2	0	1	321	17	337	2.5E-168
GAP16_sc29000_LAMovo_SSU1444	1	seq768	134	99.377	321	2	0	1	321	17	337	2.5E-168
GAP15_sc28432_LAMrrs_SSU0387	2	seq706	146	99.383	324	0	2	1	324	8	329	1.9E-169
GAP13_sc26310_PHYver_SSU0311	9	seq195	1352	99.429	350	1	1	1	349	8	357	0
GAP15_sc28479_PHYver_SSU0316	1	seq195	1352	99.429	350	1	1	1	349	8	357	0
GAP15_sc28376_LAMarc_SSU0385	3	seq803	143	99.673	306	1	0	1	306	20	325	1.1E-161
GAP16_sc29185_PHYals_SSU1526	1	seq128	2067	99.682	314	1	0	1	314	11	324	4E-166
GAP15_sc28408_LAMsau_SSU0417	15	seq1272	64	99.684	316	1	0	1	316	11	326	3.1E-167
GAP13_sc26443_LAMsau_SSU0438	12	seq171	1868	99.684	316	1	0	1	316	8	323	3.1E-167
GAP17_sc29707_DDYdub_	2	seq296	806	99.685	317	1	0	1	317	11	327	8.8E-168
GAP16_sc29059_LEPcha_SSU1302	1	seq688	153	99.685	317	1	0	1	317	8	324	8.8E-168
GAP15_sc28442_LEPcha_SSU0025	1	seq688	153	99.685	317	1	0	1	317	8	324	2.4E-168
GAP13_sc26371_LAMovo_SSU0453	14	seq768	134	99.688	321	1	0	1	321	17	337	5.3E-170
GAP15_sc28491_DIDniv_SSU0142	9	seq23	5189	99.69	323	1	0	1	323	20	342	4.1E-171
GAP16_sc29257_DIDmey_SSU1366	1	seq2235	31	99.693	326	1	0	1	326	8	333	9E-173

Supplementarty S5 data for: A four year survey reveals a coherent pattern between distribution of fruit bodies and soil amoebae populations for nivicolous myxomycetes. M. Borg Dahl, O. Shchepin, C. Schunk, A. Menzel, Y. K. Novozhilov and M. Schnittler

**Fruit match to soil OTU**												
GAP16_sc29046_DIDmey_SSU1363	1	seq2235	31	99.693	326	1	0	1	326	8	333	2.5E-173
GAP15_sc28396_DIDmey_SSU0152	79	seq2235	31	100	326	0	0	1	326	8	333	1.9E-174
GAP15_sc28521_LAMsau_SSU0420	47	seq171	1868	100	316	0	0	1	316	8	323	6.7E-169
GAP13_sc26364_DIDalp_SSU0252	42	seq7558	9	100	316	0	0	1	316	11	326	6.7E-169
GAP13_sc26288_LAMesp_SSU0002	25	seq146	1344	100	360	0	0	1	360	11	370	0
GAP15_sc28467_DIDgloeur_SSU0053	24	seq41	3217	100	320	0	0	1	320	20	339	4.1E-171
GAP13_sc26302_LEPcha_SSU0038	17	seq1945	36	100	321	0	0	1	321	14	334	1.1E-171
GAP15_sc28358_LAMovo_SSU0488	13	seq155	1711	100	321	0	0	1	321	17	337	1.1E-171
GAP16_sc29035_LAMaen_SSU1500	11	seq1474	52	100	319	0	0	1	319	20	338	1.5E-170
GAP13_sc26390_DIDfal_SSU0015	11	seq6261	11	100	320	0	0	1	320	17	336	4.1E-171
GAP15_sc28476_PHYver_SSU0317	11	seq195	1352	100	350	0	0	1	350	8	357	0
GAP15_sc28489_LAMovo_SSU0460	10	seq768	134	100	321	0	0	1	321	17	337	1.1E-171
GAP15_sc28500_LEPcha_SSU0027	10	seq688	153	100	317	0	0	1	317	8	324	1.9E-169
GAP15_sc28456_LAMsau_SSU0407	8	seq1272	64	100	316	0	0	1	316	11	326	6.7E-169
GAP17_sc29701_DDYdub_	6	seq714	145	100	317	0	0	1	317	17	333	1.9E-169
GAP13_sc26327_DDYdif_SSU0302	6	seq481	328	100	317	0	0	1	317	8	324	1.9E-169
GAP13_sc26329_LAMaen_SSU0402	5	seq426	343	100	319	0	0	1	319	20	338	1.5E-170
GAP13_sc26415_LAMaen_SSU0398	5	seq776	123	100	319	0	0	1	319	20	338	1.5E-170
GAP13_sc26426_DDYdub_SSU0294	4	seq296	806	100	317	0	0	1	317	11	327	1.9E-169
GAP15_sc28354_MERcar_SSU0351	4	seq5	14109	100	316	0	0	1	316	17	332	6.7E-169
GAP15_sc28381_MERcar_	4	seq8412	8	100	316	0	0	1	316	8	323	6.7E-169
GAP16_sc28979_MERcar_SSU1606	3	seq237	886	100	316	0	0	1	316	8	323	6.7E-169
GAP15_sc28460_DIDmic_SSU0236	3	seq580	231	100	326	0	0	1	326	17	342	1.9E-174
GAP13_sc26407_LAMaen_SSU0405	3	seq374	527	100	319	0	0	1	319	20	338	1.5E-170
GAP15_sc28435_PHYals_SSU0364	2	seq128	2067	100	314	0	0	1	314	11	324	8.7E-168
GAP15_sc28369_LAMarc_SSU0386	2	seq117	2087	100	306	0	0	1	306	20	325	2.4E-163
GAP13_sc26344_MERcar_SSU0353	2	seq21	6561	100	316	0	0	1	316	14	329	6.7E-169
GAP13_sc26341_MERspi_SSU0347	2	seq6657	10	100	317	0	0	1	317	14	330	1.9E-169
GAP13_sc26358_DIDgloeur_SSU0078	2	seq151	1682	100	320	0	0	1	320	11	330	4.1E-171
GAP16_sc29161_DIDgloeur_SSU1397	2	seq6688	10	100	320	0	0	1	320	17	336	4.1E-171
GAP16_sc29043_MERcar_	2	seq4639	14	100	316	0	0	1	316	8	323	6.7E-169
GAP15_sc28440_LAMpir_SSU0500	1	seq62	2955	100	308	0	0	1	308	17	324	1.8E-164
GAP15_sc28421_PHYalb_SSU0328	1	seq3378	20	100	337	0	0	1	337	11	347	1.5E-180
GAP13_sc26409_LAMcrs_	1	seq3857	17	100	306	0	0	1	306	14	319	2.4E-163
GAP15_sc28470_DIDniv_SSU0096	1	seq23	5189	100	323	0	0	1	323	20	342	8.9E-173

Supplementary S5 data for: A four year survey reveals a coherent pattern between distribution of fruit bodies and soil amoebae populations for nivicolous myxomycetes. M. Borg Dahl, O. Shchepin, C. Schunk, A. Menzel, Y. K. Novozhilov and M. Schnittler

No match cluster members

Members of no match clusters	taxa	ribo nr	Sc	survey	specimens	ribotypes			
GAP16_sc29108_DIDfal_SSU1309	DIDfal	43	29108	GAP16	79	17	total		
GAP16_sc29162_DIDfal_SSU1314	DIDfal	43	29162	GAP16	21	8	GAP13		
GAP16_sc29165_DIDfal_SSU1315	DIDfal	43	29165	GAP16	24	7	GAP15		
GAP16_sc29174_DIDfal_SSU1316	DIDfal	43	29174	GAP16	34	10	GAP16		
GAP13_sc26304_LAMaen_SSU0396	LAMaen	39	26304	GAP13				57	% not GAP16
GAP15_sc28510_LAMaen_	LAMaen	39	28510	GAP15				60	% not GAP16
GAP13_sc26448_LAMalb_SSU0379	LAMalb	37	26448	GAP13					
GAP13_sc26333_LAMalb_SSU0381	LAMalb	37	26333	GAP13					
GAP13_sc26433_LAMalb_SSU0375	LAMalb	37	26433	GAP13					
GAP13_sc26450_LAMalb_SSU0378	LAMalb	37	26450	GAP13					
GAP13_sc26381_LAMalb_	LAMalb	37	26381	GAP13					
GAP15_sc28516_LAMalb_SSU0377	LAMalb	37	28516	GAP15					
GAP15_sc28447_LAMalb_SSU0382	LAMalb	37	28447	GAP15					
GAP16_sc28990_LAMalb_SSU1514	LAMalb	37	28990	GAP16					
GAP16_sc29155_LAMalb_SSU1515	LAMalb	37	29155	GAP16					
GAP16_sc29175_LAMalb_SSU1516	LAMalb	37	29175	GAP16					
GAP16_sc29210_LAMalb_SSU1517	LAMalb	37	29210	GAP16					
GAP16_sc29050_LAMalb_SSU1518	LAMalb	37	29050	GAP16					
GAP16_sc29118_LAMalb_SSU1519	LAMalb	37	29118	GAP16					
GAP16_sc29051_LAMalb_SSU1520	LAMalb	37	29051	GAP16					
GAP13_sc26394_LAMovo_SSU0447	LAMovo	2	26394	GAP13					
GAP13_sc26391_LAMovo_SSU0449	LAMovo	2	26391	GAP13					
GAP13_sc26395_LAMovo_SSU0446	LAMovo	2	26395	GAP13					
GAP13_sc26392_LAMovo_SSU0450	LAMovo	2	26392	GAP13					
GAP13_sc26338_LAMovocuc_SSU0448	LAMovo	2	26338	GAP13					
GAP13_sc26326_LAMovo_SSU0445	LAMovo	17	26326	GAP13					
GAP13_sc26297_LAMovo_SSU0472	LAMovo	20	26297	GAP13					
GAP13_sc26393_LAMovo_SSU0480	LAMovo	20	26393	GAP13					
GAP13_sc26416_LAMovo_SSU0483	LAMovo	20	26416	GAP13					
GAP13_sc26365_LAMovo_SSU0486	LAMovo	20	26365	GAP13					
GAP15_sc28449_LAMovo_SSU0468	LAMovo	20	28449	GAP15					
GAP15_sc28416_LAMovo_SSU0470	LAMovo	20	28416	GAP15					
GAP15_sc28422_LAMovo_SSU0474	LAMovo	20	28422	GAP15					
GAP15_sc28379_LAMovo_SSU0476	LAMovo	20	28379	GAP15					
GAP15_sc28368_LAMovo_SSU0478	LAMovo	20	28368	GAP15					
GAP15_sc28497_LAMovo_SSU0469	LAMovo	20	28497	GAP15					
GAP15_sc28383_LAMovo_SSU0471	LAMovo	20	28383	GAP15					
GAP15_sc28502_LAMovo_SSU0473	LAMovo	20	28502	GAP15					
GAP15_sc28373_LAMovo_SSU0475	LAMovo	20	28373	GAP15					
GAP15_sc28544_LAMovo_SSU0477	LAMovo	20	28544	GAP15					
GAP15_sc28366_LAMovo_SSU0479	LAMovo	20	28366	GAP15					
GAP15_sc28501_LAMovo_SSU0481	LAMovo	20	28501	GAP15					
GAP15_sc28386_LAMovo_	LAMovo	20	28386	GAP15					
GAP15_sc28409_LAMovo_	LAMovo	20	28409	GAP15					
GAP15_sc28359_LAMovo_	LAMovo	20	28359	GAP15					
GAP15_sc28451_LAMovo_	LAMovo	20	28451	GAP15					
GAP16_sc28951_LAMovo_SSU1414	LAMovo	20	28951	GAP16					
GAP16_sc28961_LAMovo_SSU1415	LAMovo	20	28961	GAP16					
GAP16_sc28975_LAMovo_SSU1416	LAMovo	20	28975	GAP16					
GAP16_sc29003_LAMovo_SSU1417	LAMovo	20	29003	GAP16					
GAP16_sc29169_LAMovo_SSU1418	LAMovo	20	29169	GAP16					
GAP16_sc28970_LAMovo_SSU1420	LAMovo	20	28970	GAP16					
GAP16_sc29092_LAMovo_SSU1419	LAMovo	20	29092	GAP16					
GAP16_sc29001_LAMovo_SSU1421	LAMovo	20	29001	GAP16					
GAP16_sc29020_LAMovo_SSU1422	LAMovo	20	29020	GAP16					
GAP16_sc29067_LAMovo_SSU1423	LAMovo	20	29067	GAP16					
GAP16_sc29064_LAMovo_SSU1424	LAMovo	20	29064	GAP16					
GAP16_sc29095_LAMovo_SSU1425	LAMovo	20	29095	GAP16					
GAP16_sc29167_LAMovo_SSU1427	LAMovo	20	29167	GAP16					
GAP16_sc28952_LAMovo_SSU1426	LAMovo	20	28952	GAP16					
GAP16_sc29040_LAMovo_SSU1428	LAMovo	20	29040	GAP16					
GAP16_sc28989_LAMovo_SSU1443	LAMovo	57	28989	GAP16					
GAP16_sc29089_LAMpul_SSU1513	LAMpul	60	29089	GAP16					

Supplementary S5 data for: A four year survey reveals a coherent pattern between distribution of fruit bodies and soil amoebae populations for nivicolous myxomycetes. M. Borg Dahl, O. Shchepin, C. Schunk, A. Menzel, Y. K. Novozhilov and M. Schnittler

No match cluster members				
GAP13_sc26436_LAMsau_SSU0406	LAMsau	59	26436	GAP13
GAP15_sc28394_LAMzon_SSU0443	LAMzon	5	28394	GAP15
GAP16_sc29078_LAMzon_SSU1447	LAMzon	5	29078	GAP16
GAP16_sc29079_LAMzon_SSU1448	LAMzon	5	29079	GAP16
GAP13_sc26335_MERagg_SSU0338	MERagg	12	26335	GAP13
GAP15_sc28401_MERagg_	MERagg	13	28401	GAP15
GAP13_sc26382_MERagg_SSU0345	MERagg	34	26382	GAP13
GAP13_sc26330_MERagg_SSU0342	MERagg	34	26330	GAP13
GAP13_sc26384_MERagg_	MERagg	34	26384	GAP13
GAP15_sc28404_MERagg_SSU0341	MERagg	34	28404	GAP15
GAP15_sc28397_MERagg_	MERagg	34	28397	GAP15
GAP16_sc29025_MERagg_SSU1612	MERagg	34	29025	GAP16
GAP16_sc29097_MERagg_SSU1613	MERagg	52	29097	GAP16
GAP15_sc28398_MERagg_	MERagg	55	28398	GAP15
GAP16_sc29163_MERech_SSU1610	MERech	54	29163	GAP16
GAP16_sc29172_PHYalb_SSU1527	PHYalb	51	29172	GAP16

S6 - Supplementary data for: A four year survey reveals a coherent pattern between occurrence of fruit bodies and soil amoebae populations for nivicolous myxomycetes. M. Borg Dahl, O. Shchepin, C. Schunk, A. Menzel, Y. K. Novozhilov and M. Schnittler

2015/16

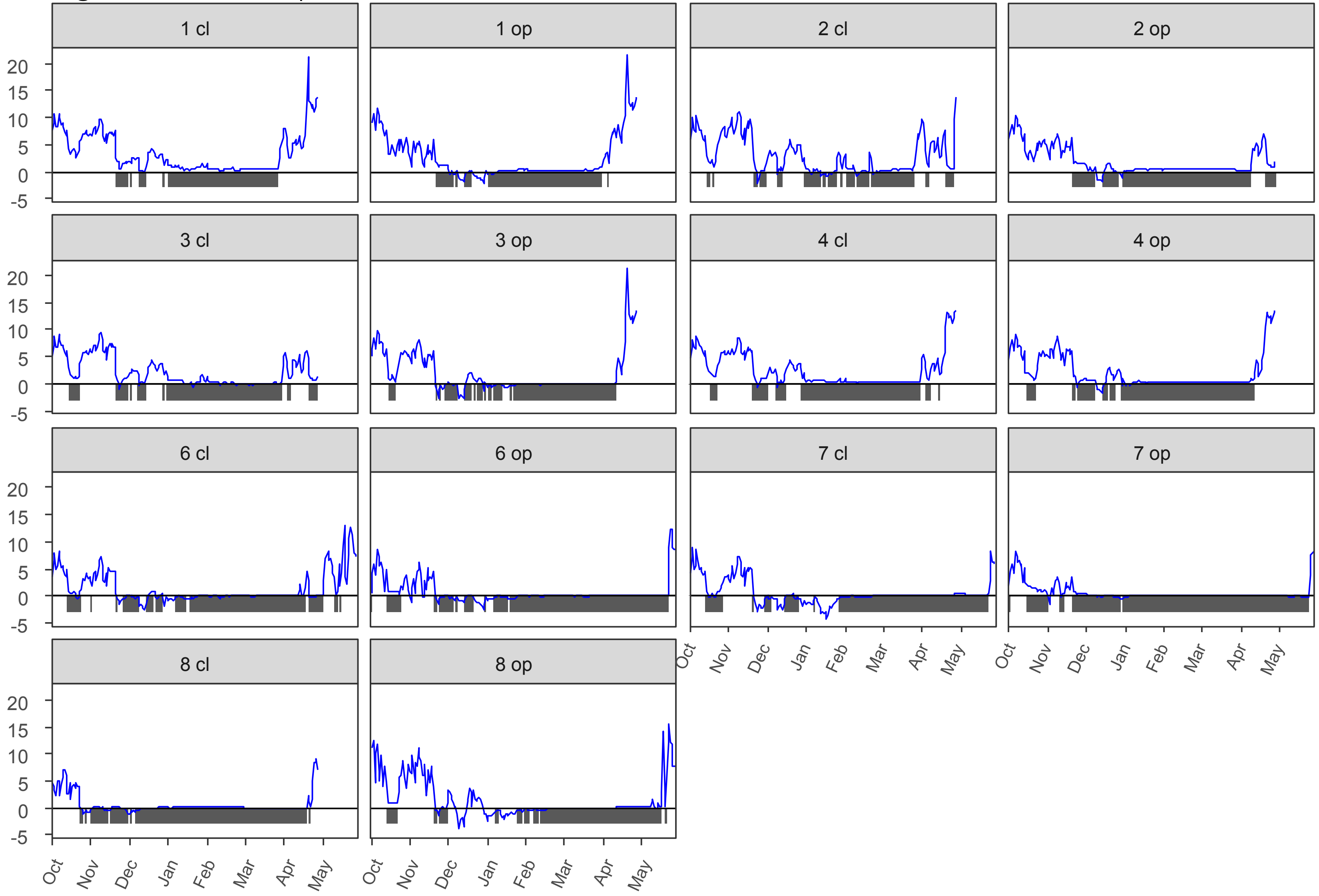

Temperature (blue line) plots from Winter 2015 from all sites. Grey bars represents days with suitable condition for amoebal growth (estimated as days with a daily average temperature between –0.5 and 2°C and fluctuations below 3°C, indicating the presence of an insulating layer of snow). Data from open and closed canopy sites at elevation level 5 (1,400m) were lost.

S6 - Supplementary data for: A four year survey reveals a coherent pattern between occurrence of fruit bodies and soil amoebae populations for nivicolous myxomycetes. M. Borg Dahl, O. Shchepin, C. Schunk, A. Menzel, Y. K. Novozhilov and M. Schnittler

2016/17

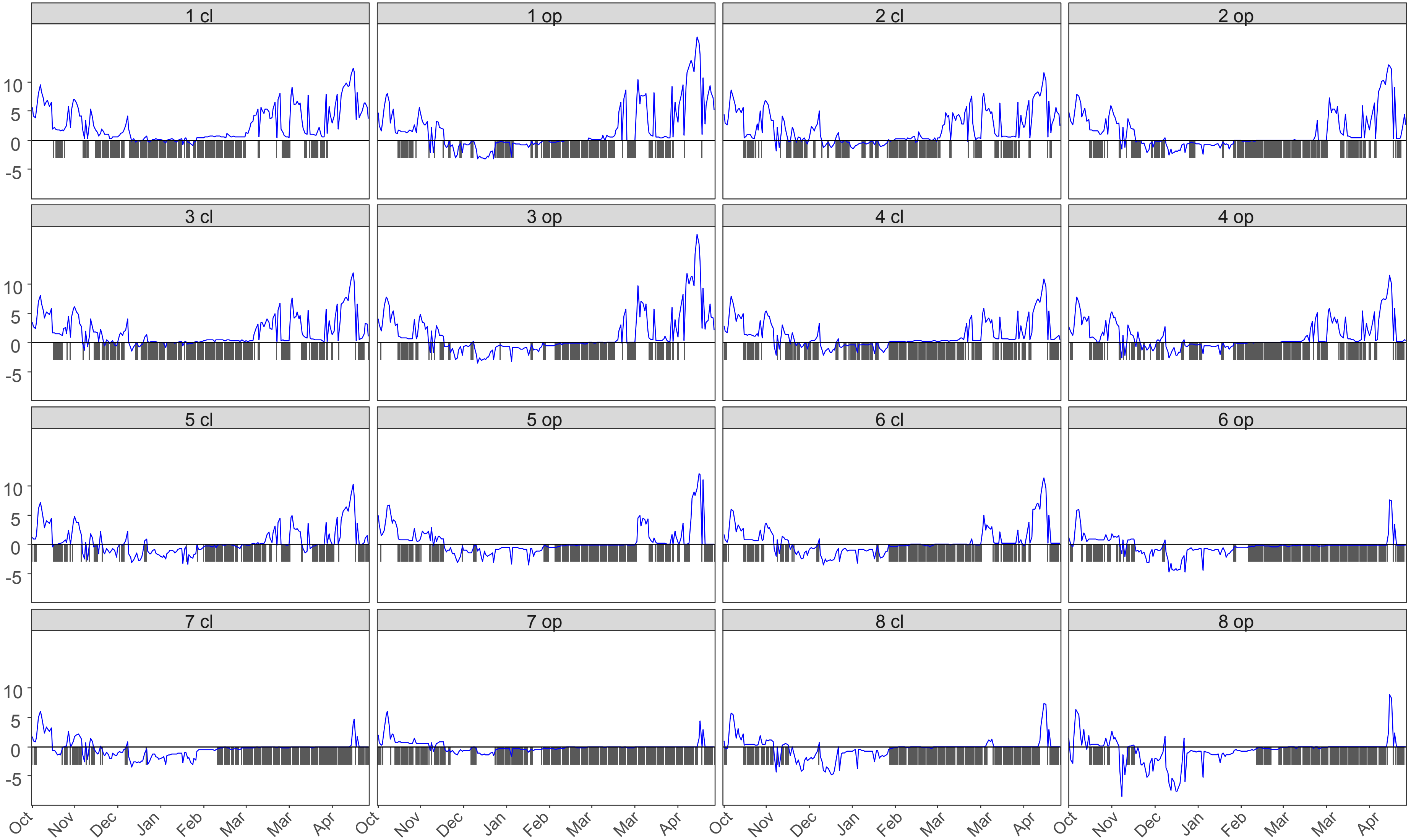

Temperature (blue line) plots from Winter 2016 from all sites. Grey bars represents days with suitable condition for amoebal growth (estimated as days with a daily average temperature between –0.5 and 2°C and fluctuations below 3°C, indicating the presence of an insulating layer of snow).

```
#### Supplementary S7 data for: A four year survey reveals a coherent pattern
between occurence of fruit bodies and soil amoebae populations for nivicolous
myxomycetes. M. Borg Dahl, O. Shchepin, C. Schunk, A. Menzel, Y. K. Novozhilov and
M. Schnittler
#### S7 - Analysis script


############ Fruit body seq
############################

#### Dereplicate to identify ribotypes

C:\Users\Botany>Usearch9 -derep_fulllength GAP13-17.fas -sizeout -fastaout
uniquesGAP.fa  -uc clustersize.txt
usearch v9.0.2132_win32, 2.0Gb RAM (4.1Gb total), 4 cores
(C) Copyright 2013-15 Robert C. Edgar, all rights reserved.
http://drive5.com/usearch

License: mathildeborg.dahl@uni-greifswald.de

00:00 4.6Mb   100.0% Reading GAP13-17.fas
00:00 4.6Mb   100.0% DF
00:00 4.7Mb  533 seqs, 70 uniques, 22 singletons (31.4%)
00:00 4.7Mb  Min size 1, median 3, max 79, avg 7.61
00:00 4.7Mb   100.0% Writing uniquesGAP.fa
00:00 4.7Mb   100.0% Writing clustersize.txt

### cluster unique ribotypes in 99.1% ribotype clusters

C:\Users\Botany>Usearch9 -cluster_otus uniquesGAP.fa  -otus OTUribo.fa -minsize 1
-otu_radius_pct 0.9 -uparseout oturibomember.txt
usearch v9.0.2132_win32, 2.0Gb RAM (4.1Gb total), 4 cores
(C) Copyright 2013-15 Robert C. Edgar, all rights reserved.
http://drive5.com/usearch

License: mathildeborg.dahl@uni-greifswald.de

00:00 7.5Mb   100.0% 33 OTUs, 0 chimeras

### The algorithm ignores the threshold (-otu_radius_pct 0.9), but the ribo-
clusters can be correctly identified from the output file 'oturibomember.txt'.

#### Build database consisting of the soil OTU sequences (denoted denoisedMYX.fa)
######
C:\Users\Botany>makeblastdb -in Myx_OTUs_09clust_209OTUs.fas -input_type fasta
-dbtype nucl -title Database_soilOTU_13072018 -parse_seqids -out
Database_soilOTU_13072018


Building a new DB, current time: 07/13/2018 11:19:16
New DB name:   C:\Users\Botany\Database_soilOTU_13072018
New DB title:  Database_soilOTU_13072018
Sequence type: Nucleotide
Keep MBits: T
Maximum file size: 1000000000B
Adding sequences from FASTA; added 208 sequences in 0.102851 seconds.


#### Blast fruit body ribotypes to the soil OTU ######
```

```
C:\Users\Botany>Desktop\blast\bin\blastn -query uniquesGAP.fa  -out
Matchsoil_to_fruit_new_2018.txt -db Database_soilOTU_13072018 -outfmt 6
-max_target_seqs 1 -best_hit_overhang 0.25


##### Diversity estimators - Chao 2014
########################################

SEE : https://cran.r-project.org/web/packages/iNEXT/vignettes/Introduction.html

all_out <- iNEXT(all, q= 0, datatype="incidence_freq")

From R 3.3.3.:
all_out

Compare 4 assemblages with Hill number order q = 0.
$class: iNEXT

$DataInfo: basic data information
         site      T      U S.obs     SC Q1 Q2 Q3 Q4 Q5 Q6 Q7 Q8 Q9 Q10
1        Ribo    532    533    45 0.9831  9  4  5  1  3  4  0  1  1   1
2      MOrpho    710    710    28 0.9958  3  2  4  0  0  1  0  1  1   0
3    OTU read 838553 838553   208 1.0000  3  6  2  0  0  2  2  0  0   0
4 soil morpho 340829 340727    33 1.0000  2  0  0  0  0  0  0  0  0   0

$iNextEst: diversity estimates with rarefied and extrapolated samples.
$Ribo
      t       method order     qD qD.LCL qD.UCL    SC SC.LCL SC.UCL
1     1 interpolated     0  1.002  0.902  1.101 0.062  0.051  0.072
10  266 interpolated     0 38.658 35.148 42.168 0.964  0.954  0.975
20  532     observed     0 45.000 39.883 50.117 0.983  0.975  0.992
30  784 extrapolated     0 48.476 42.114 54.838 0.989  0.979  0.999
40 1064 extrapolated     0 50.955 42.993 58.917 0.993  0.984  1.000

$MOrpho
      t       method order     qD qD.LCL qD.UCL    SC SC.LCL SC.UCL
1     1 interpolated     0  1.000  0.932  1.068 0.093  0.081  0.105
10  355 interpolated     0 25.482 23.410 27.553 0.988  0.984  0.992
20  710     observed     0 28.000 25.110 30.890 0.996  0.991  1.000
30 1046 extrapolated     0 29.052 24.971 33.132 0.998  0.993  1.000
40 1420 extrapolated     0 29.655 24.068 35.242 0.999  0.994  1.000

$`OTU read`
         t       method order      qD  qD.LCL  qD.UCL    SC SC.LCL SC.UCL
1        1 interpolated     0   1.000   0.998   1.002 0.027  0.027  0.028
10  419276 interpolated     0 204.703 202.507 206.898 1.000  1.000  1.000
20  838553     observed     0 208.000 205.068 210.932 1.000  1.000  1.000
30 1235762 extrapolated     0 208.637 204.985 212.289 1.000  1.000  1.000
40 1677106 extrapolated     0 208.736 204.266 213.206 1.000  1.000  1.000

$`soil morpho`
        t       method order     qD qD.LCL qD.UCL    SC SC.LCL SC.UCL
1       1 interpolated     0  1.000  0.996  1.003 0.054  0.054  0.055
10 170414 interpolated     0 32.000 31.464 32.536 1.000  1.000  1.000
20 340829     observed     0 33.000 32.406 33.594 1.000  1.000  1.000
30 502274 extrapolated     0 33.612 33.018 34.206 1.000  1.000  1.000
40 681658 extrapolated     0 33.865 33.271 34.459 1.000  1.000  1.000
```

```
$AsyEst: asymptotic diversity estimates along with related statistics.
          Site         Diversity Observed Estimator  s.e.     LCL     UCL
1         Ribo  Species richness   45.000    55.106 9.002  47.260  90.199
2         Ribo Shannon diversity   23.415    24.725 1.041  23.415  26.766
3         Ribo Simpson diversity   15.726    16.145 1.014  15.726  18.133
4       MOrpho  Species richness   28.000    30.247 3.391  28.265  47.015
5       MOrpho Shannon diversity   14.698    15.022 0.514  14.698  16.029
6       MOrpho Simpson diversity   10.627    10.760 0.484  10.627  11.708
7     OTU read  Species richness  208.000   208.750 1.262 208.077 215.274
8     OTU read Shannon diversity   60.165    60.172 0.081  60.165  60.330
9     OTU read Simpson diversity   36.515    36.516 0.069  36.515  36.651
10 soil morpho  Species richness   33.000    34.000 2.320  33.069  47.417
11 soil morpho Shannon diversity   21.814    21.815 0.025  21.814  21.865
12 soil morpho Simpson diversity   18.379    18.380 0.032  18.379  18.443

NOTE: Only show five estimates, call iNEXT.object$iNextEst. to show complete
output.


####### Correlation between shared sequences (soil and fruit) and number of
sequenced fruit bodies (sumF)
> cor.test(Ematch$sumF,Ematch$`shared seq`)

      Pearson's product-moment correlation

data:  Ematch$sumF and Ematch$`shared seq`
t = 6.6705, df = 6, p-value = 0.0005494
alternative hypothesis: true correlation is not equal to 0
95 percent confidence interval:
 0.6913635 0.9891059
sample estimates:
      cor
0.9387106


####### Correlation between unique fruit ribotypes and number of sequenced fruit
bodies (sumF)
> cor.test(Ematch$sumF,Ematch$`Unique Fruit ribo`)

      Pearson's product-moment correlation

data:  Ematch$sumF and Ematch$`Unique Fruit ribo`
t = 11.645, df = 6, p-value = 2.416e-05
alternative hypothesis: true correlation is not equal to 0
95 percent confidence interval:
 0.8823928 0.9962566
sample estimates:
      cor
0.9785839


### Correlation between number of growth (gro) days and elevation (e) - year
2015/16
> cor.test(gro, e)

      Pearson's product-moment correlation

data:  gro and e
t = 3.4695, df = 12, p-value = 0.004635
```

```
alternative hypothesis: true correlation is not equal to 0
95 percent confidence interval:
 0.2835399 0.9002297
sample estimates:
      cor
0.7076589

##### Correlation between number of frost (fro) days and elevation (e) - year
2015/16
> cor.test(fro, e)

	Pearson's product-moment correlation

data:  fro and e
t = 1.99, df = 12, p-value = 0.06987
alternative hypothesis: true correlation is not equal to 0
95 percent confidence interval:
 -0.04412712  0.81365403
sample estimates:
      cor
0.4981147

#### Correlation between number of growth (gro) days and elevation (e) - year
2016/17
> cor.test(gro1617, e2)

	Pearson's product-moment correlation

data:  gro1617 and e2
t = 1.2721, df = 14, p-value = 0.224
alternative hypothesis: true correlation is not equal to 0
95 percent confidence interval:
 -0.2068050  0.7050949
sample estimates:
      cor
0.3218997
##### Correlation between number of frost (fro) days and elevation (e) - year
2016/17

> cor.test(fro1617, e2)

	Pearson's product-moment correlation

data:  fro1617 and e2
t = 3.4832, df = 14, p-value = 0.003655
alternative hypothesis: true correlation is not equal to 0
95 percent confidence interval:
 0.2803742 0.8798901
sample estimates:
      cor
0.6813794

 ####### Shifted distribution Elevation

Elevation_ab_ngs <- ddply(NGS_df_reduc, c("name"), summarise,
                          mean = mean(Enr),
                          sd   = sd(Enr)
                                      )
```

```
Elevation_ab_fru <- ddply(Fru_spE_reduc, c("name"), summarise,
                      mean = mean(Ecat),
                      sd   = sd(Ecat)
                      )
All <- Elevation_ab_ngs
All <- rbind(All,Elevation_ab_fru)
All$ori <- c(rep("ngs",19),rep("fru",19))

fit <- aov(mean ~ ori + name, data=All)
aov1 <- aov(fit)
> summary(aov1)
            Df Sum Sq Mean Sq F value   Pr(>F)
ori          1  16.28  16.280  15.655 0.000925 ***
name        18  33.68   1.871   1.799 0.111198
Residuals   18  18.72   1.040
---
Signif. codes:  0 ‘***’ 0.001 ‘**’ 0.01 ‘*’ 0.05 ‘.’ 0.1 ‘ ’ 1

# Tukey Honestly Significant Differences
> group_test <- HSD.test(aov1, 'ori')
$statistics
      Mean       CV  MSerror       HSD
  5.061974 20.14548 1.039908 0.6950974

$parameters
  Df ntr StudentizedRange alpha  test name.t
  18   2         2.971152  0.05 Tukey    ori

$means
        mean       std  r    Min      Max
fru 5.716506 0.9011785 19 3.6875 7.333333
ngs 4.407442 1.4487071 19 1.0000 8.000000

$comparison
NULL

$groups
  trt    means M
1 fru 5.716506 a
2 ngs 4.407442 b
```
